# Supplementary material for: Promoting biomass electrooxidation via modulating proton and oxygen anion deintercalation in hydroxide
Source: Nat Commun. 2022 Jun 30;13:3777. doi: 10.1038/s41467-022-31484-0 (PMC9246976; doi:10.1038/s41467-022-31484-0)
Supplement: Supplementary file 1 — Supplementary information [file 41467_2022_31484_MOESM1_ESM.pdf]

Supplementary Information

Promoting biomass electrooxidation via modulating proton and oxygen anion (de)intercalation in hydroxide

Zuyun He[1,4], Jinwoo Hwang[2,4], Zhiheng Gong[1], Mengzhen Zhou[1], Nian Zhang[3], Xiongwu Kang[1], Jeong Woo Han[2*], Yan Chen[1*]

[1]School of Environment and Energy, State Key Laboratory of Pulp and Paper Engineering, South China University of Technology, Guangzhou, Guangdong, 510006, China

[2]Department of Chemical Engineering, Pohang University Science and Technology, Pohang, Gyeongbuk 37673, Republic of Korea

[3]State Key Laboratory of Functional Materials for Informatics, Shanghai Institute of Microsystem and Information Technology, Chinese Academy of Sciences, Shanghai, 200050, China

[4]These authors contributed equally: Zuyun He, Jinwoo Hwang

*Corresponding authors:

E-mail addresses: escheny@scut.edu.cn (Yan Chen), jwhan@postech.ac.kr (Jeong Woo Han)

Supplementary Figures

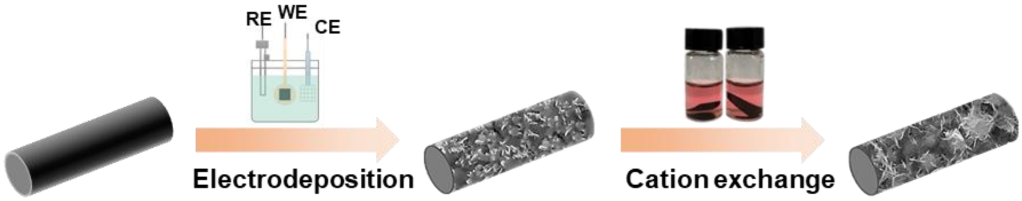

Supplementary Fig. 1. **Catalyst synthesis process.** Schematic illustration of the preparation process of NiCo hydroxide.

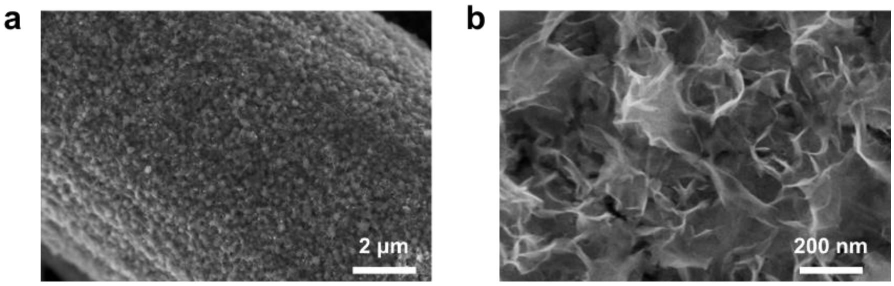

Supplementary Fig. 2. **Scanning electron microscopy (SEM) images of NiCo hydroxide. a**, low magnification. **b**, high magnification.

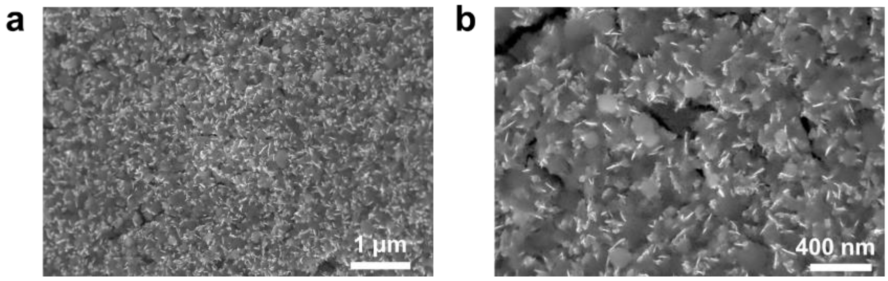

Supplementary Fig. 3. **SEM images of Ni hydroxide. a**, low magnification. **b**, high magnification.

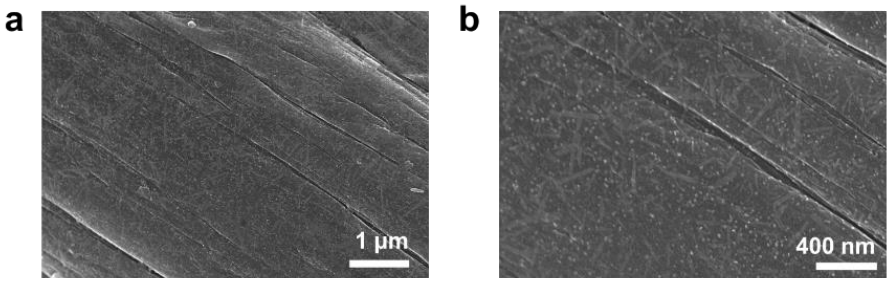

Supplementary Fig. 4. **SEM images of Co hydroxide**. **a**, low magnification. **b**, high magnification.

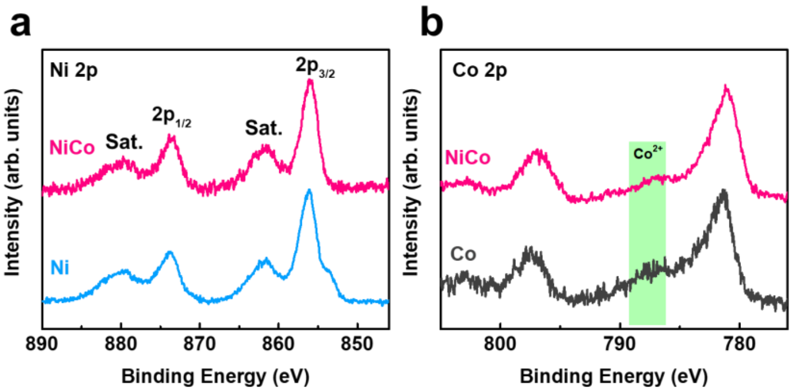

Supplementary Fig. 5. **X-ray photoelectron spectroscopy (XPS) spectra. a**, Ni 2p XPS spectra of Ni and NiCo hydroxide. **b,** Co 2p XPS spectra of Co and NiCo hydroxide.

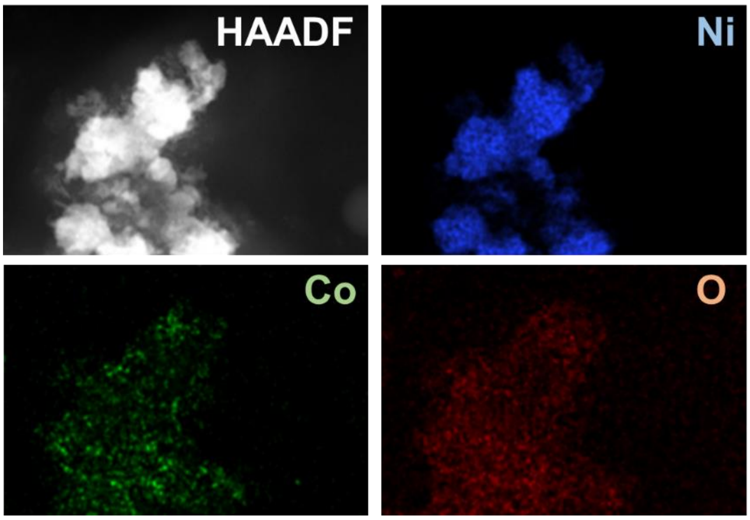

Supplementary Fig. 6. Element distribution in NiCo hydroxide. High-angle annular dark-field scanning transmission electron microscope (HAADF-STEM) image of NiCo hydroxide and the corresponding energy dispersive spectroscopy (EDS) mapping of Ni, Co and O.

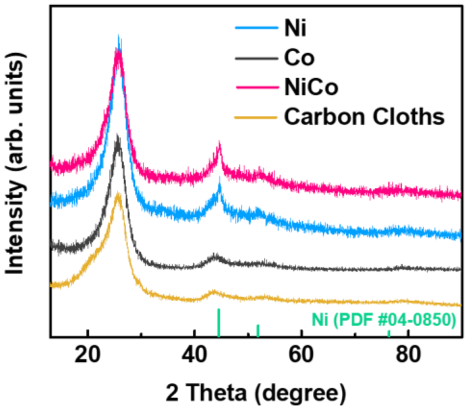

Supplementary Fig. 7. Crystal structure analysis. X-ray diffraction (XRD) patterns of Ni, Co, NiCo hydroxide and carbon cloths substrate.

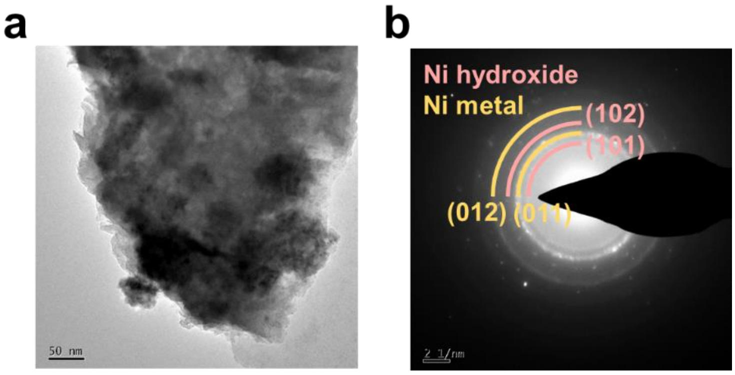

Supplementary Fig. 8. **Morphology and crystal structure characterization of Ni hydroxide. a**, Transmission electron microscopy (TEM) image. **b**, The corresponding selected area electron diffraction (SAED) pattern.

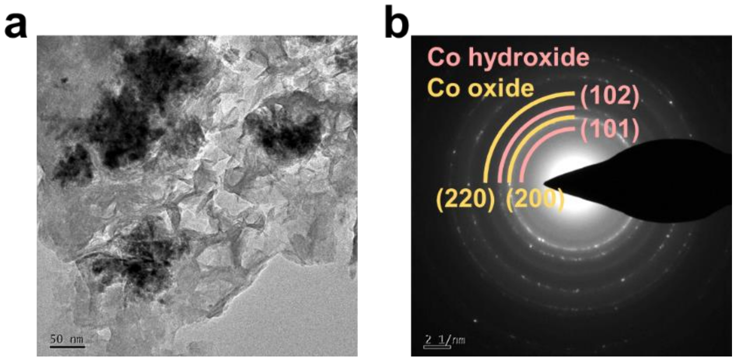

Supplementary Fig. 9. **Morphology and crystal structure characterization of Co hydroxide. a**, TEM image. **b**, SAED pattern.

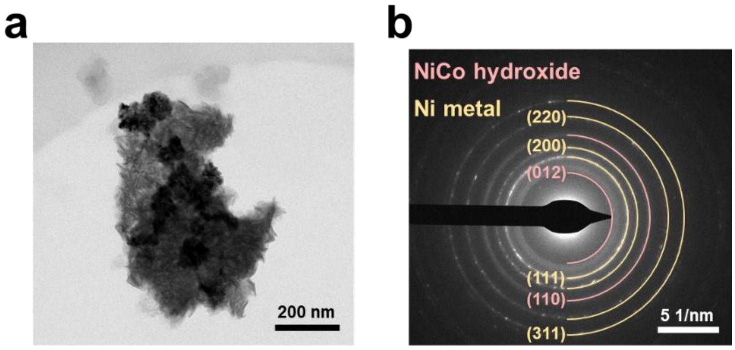

Supplementary Fig. 10. **Morphology and crystal structure characterization of NiCo hydroxide. a**, TEM image. **b**, SAED pattern.

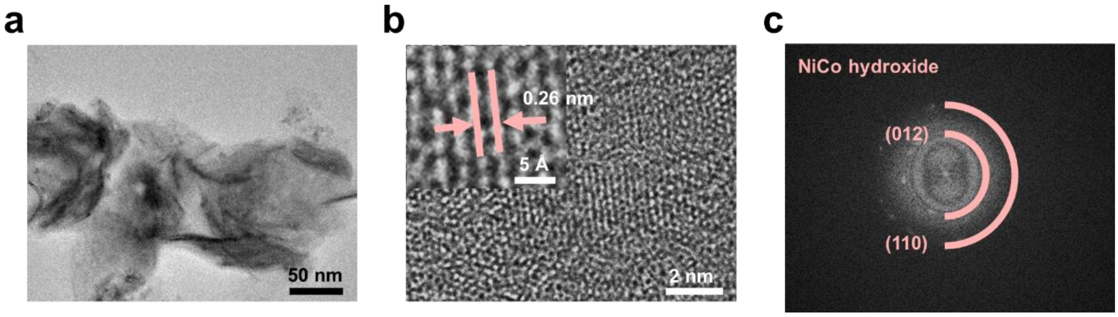

Supplementary Fig. 11. **Morphology and crystal structure characterization of NiCo hydroxide.** High-resolution transmission electron microscopy (HRTEM) image with **a**, low magnification and **b**, high magnification. **c**, The corresponding Fast Fourier transform (FFT) pattern of the selected region.

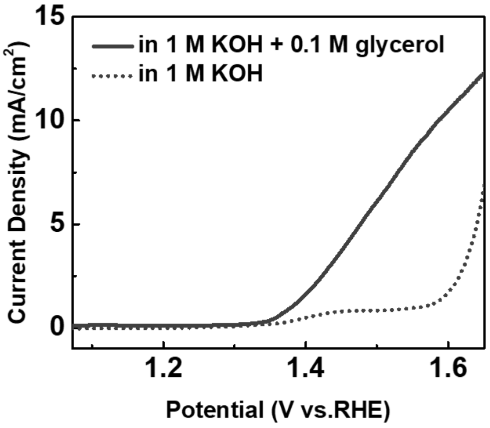

Supplementary Fig. 12. **GOR and OER performance comparison of Co hydroxide.** Cyclic voltammetry curves (forward) of Co hydroxide in 1 M KOH solution with (solid line) or without (dash line) 0.1 M glycerol.

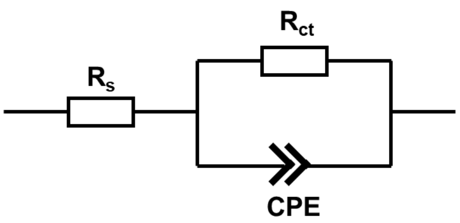

Supplementary Fig. 13. The equivalent circuit used for electrochemical impedance spectroscopies fitting. The R_s, CPE, and R_{ct} refer to Ohmic resistance, constant phase element, and charge-transfer resistance, respectively.

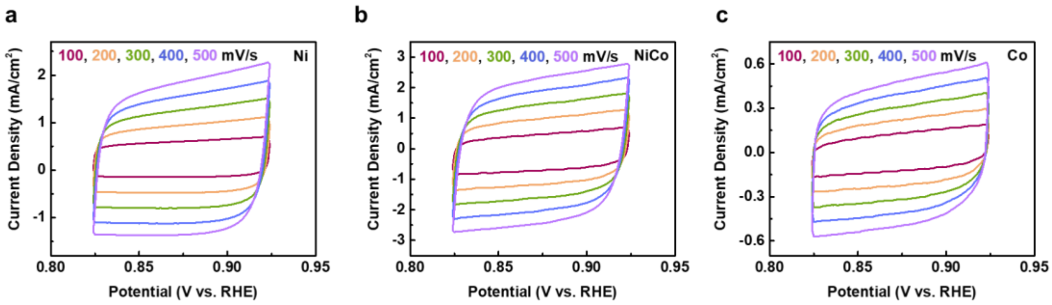

Supplementary Fig. 14. Cyclic voltammetry curves with different scan rate (100 mV/s - 500 mV/s) used for electrochemical surface area evaluation. a, Ni hydroxide. **b**, NiCo hydroxide. **c**, Co hydroxide.

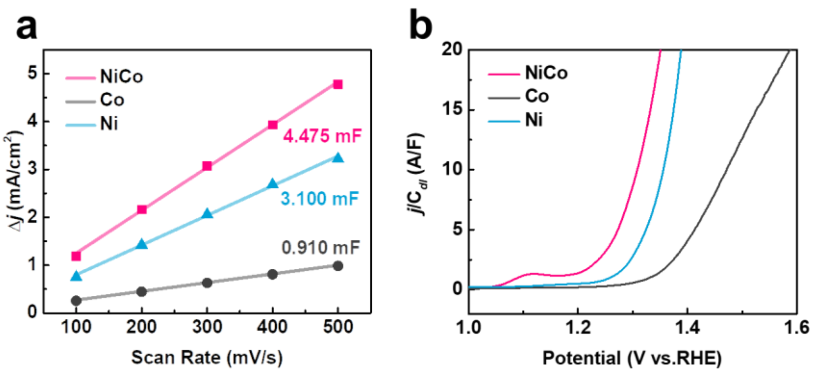

Supplementary Fig. 15. Specific activity of Ni, NiCo, and Co hydroxide. a, Double-layer capacitance. **b**, LSV curves normalized by double-layer capacitance.

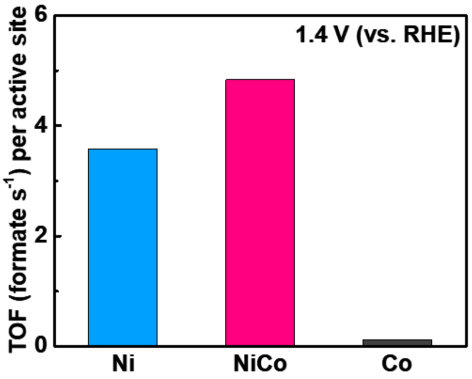

Supplementary Fig. 16. The turnover frequency (TOF) calculation results. TOF for GOR on Ni, NiCo, and Ni hydroxide at a potential of 1.4 V (vs. RHE).

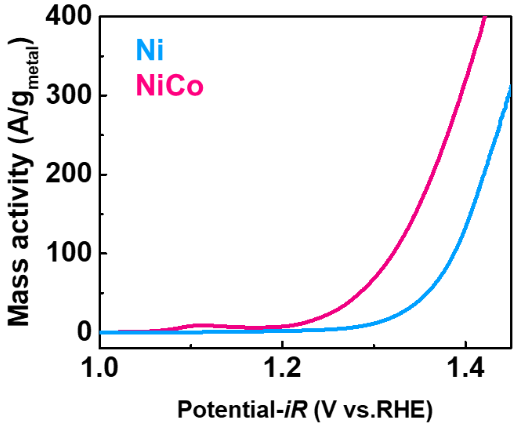

Supplementary Fig. 17. Mass activity of Ni and NiCo hydroxide. The Current density was normalized by loading mass obtained from inductively coupled plasma-optical emission spectrometry (ICP-OES) measurement.

8

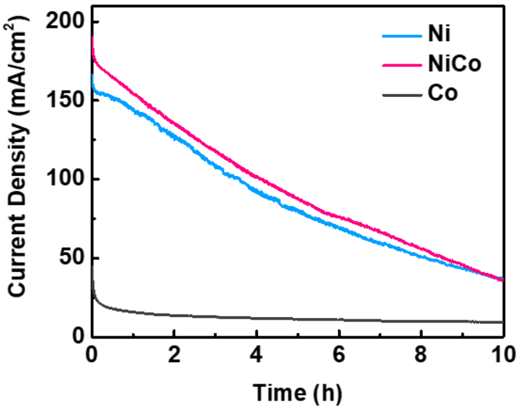

Supplementary Fig. 18. The i-t curves during long-time electrolysis for product analysis. The evolution of current density as a function of electrolysis time.

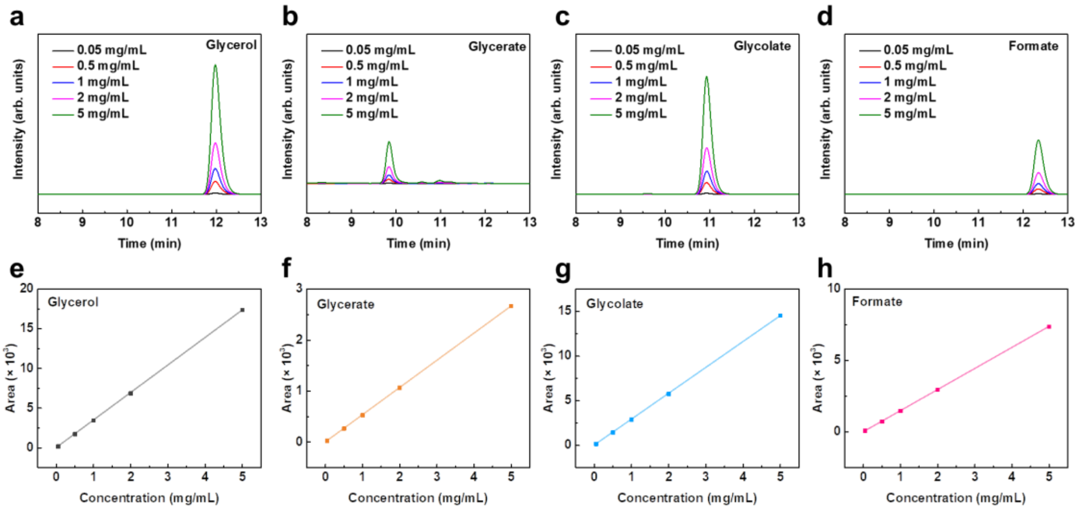

Supplementary Fig. 19. **Standard high-performance liquid chromatography (HPLC) chromatograms and the corresponding calibration curves. a**, **e**, glycerol. **b**, **f**, glycerate. **c**, **g**, glycolate. **d**, **h**, formate. The corresponding calibration curves were used to quantify the concentrations of the respective component in electrolytes after electrolyzing.

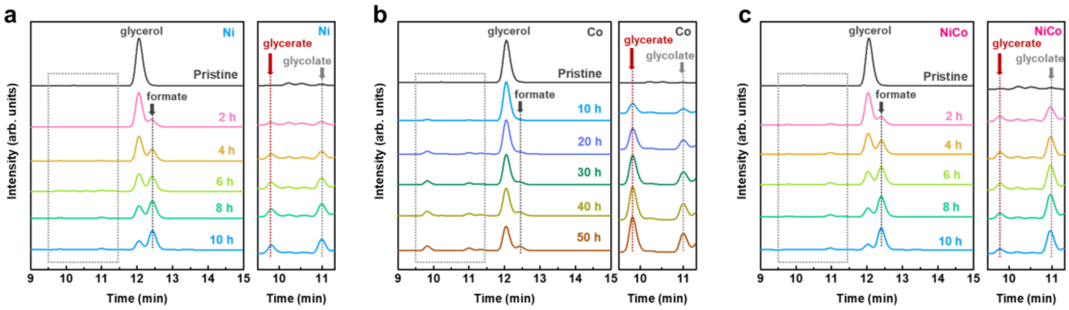

Supplementary Fig. 20. The evolution of HPLC chromatograms as a function of electrolyzing time. a, Ni hydroxide. **b**, Co hydroxide. **c**, NiCo hydroxide. The electrolysis experiments were carried out on a constant applied potential of 1.624 V (vs. RHE) in 50 mL electrolyte of 1 M KOH with 0.1 M glycerol. The right panels are the enlarged figures of the areas marked by the dash line.

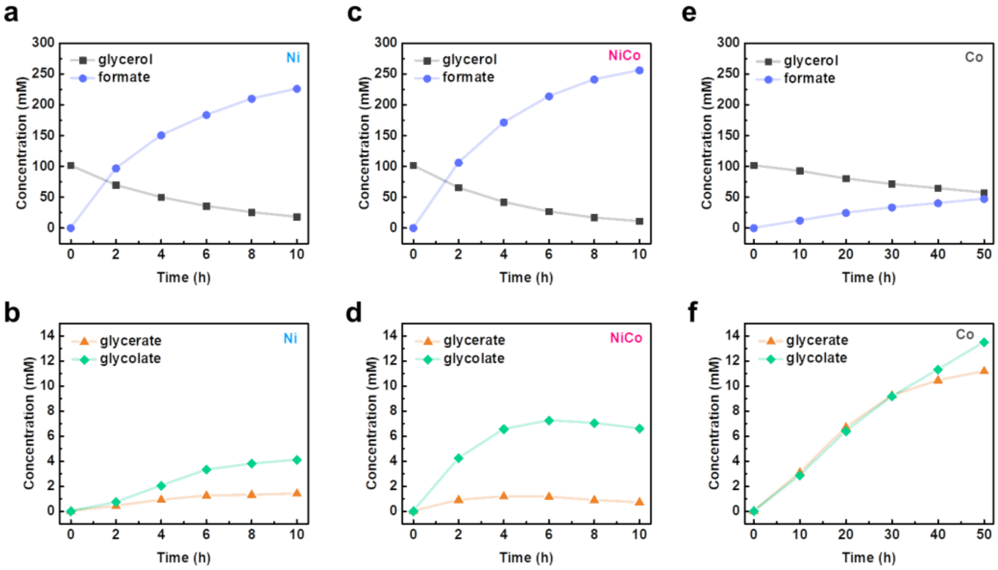

Supplementary Fig. 21. Concentration of glycerol and GOR products as a function of electrolysis time. a-b, Ni hydroxide. **c-d**, NiCo hydroxide. **e-f**, Co hydroxide.

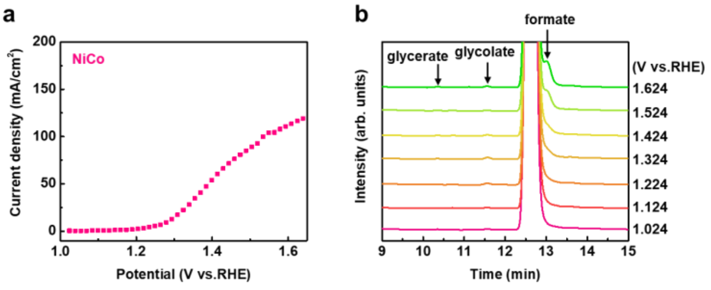

Supplementary Fig. 22. Reaction product analysis during LSV measurements. a, LSV curve of NiCo hydroxide during online collection of reaction products for HPLC measurement. The scan rate is 1 mV/s. **b**, The ex-situ HPLC chromatograms of reaction products collecting at different potential during LSV measurement on NiCo hydroxide. **c**, The evolution of product concentration as a function of the applied potential, in which the product concentrations were obtained based on the corresponding HPLC chromatograms.

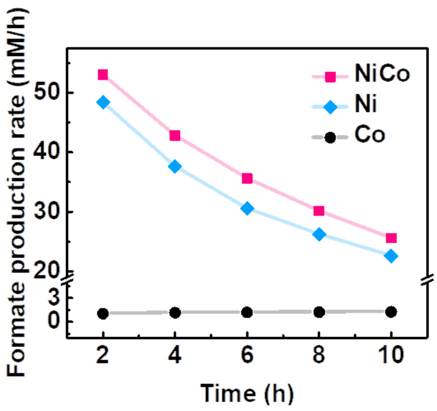

Supplementary Fig. 23. Formate production rate of Ni, NiCo, and Co hydroxide. The evolution of formate production rate as a function of electrolysis time.

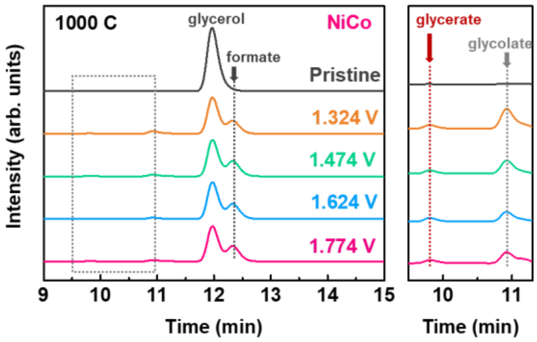

Supplementary Fig. 24. The HPLC chromatograms used for Faraday efficiency calculation of NiCo hydroxide. The HPLC chromatograms of electrolyte after GOR using NiCo hydroxide as electrocatalyst at different applied potentials (1.324, 1.474, 1.624, 1.774 V vs. RHE) with the same amount of total charge passed (1000 C). The right panels are the enlarged figures of the dash line areas.

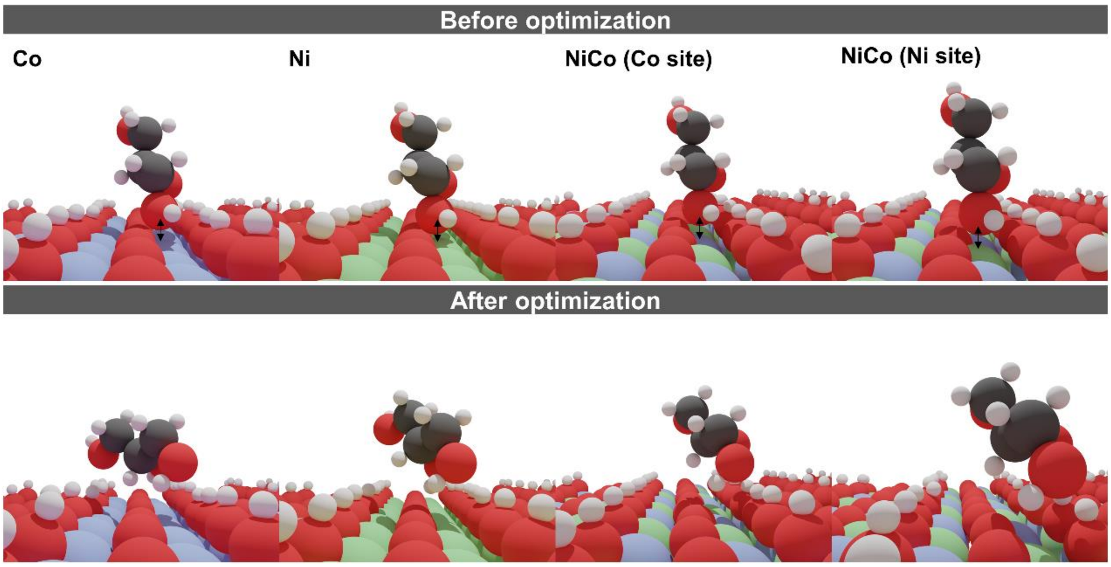

Supplementary Fig. 25. Glycerol adsorption sites calculations. Adsorption structures of glycerol on metal site before and after optimization.

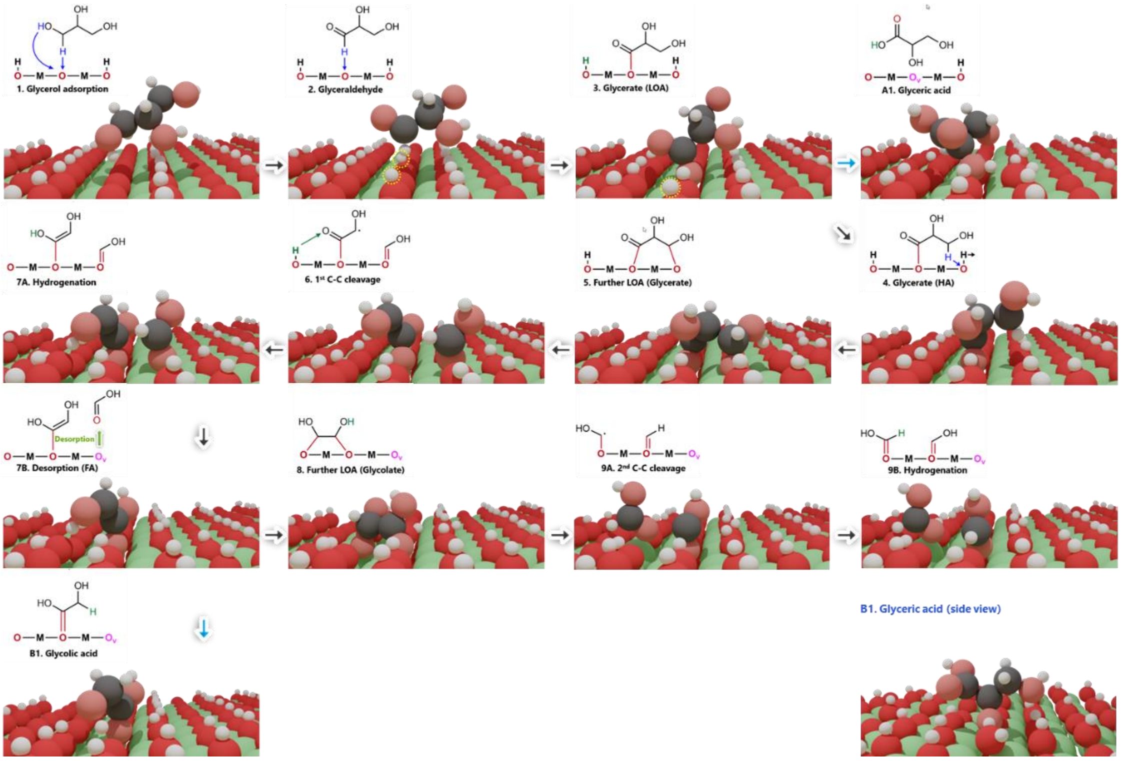

Supplementary Fig. 26. The configuration of the adsorbed intermediates on Ni hydroxide. The H atom in dash circle is from the surface reaction intermediate during the dehydrogenation reaction.

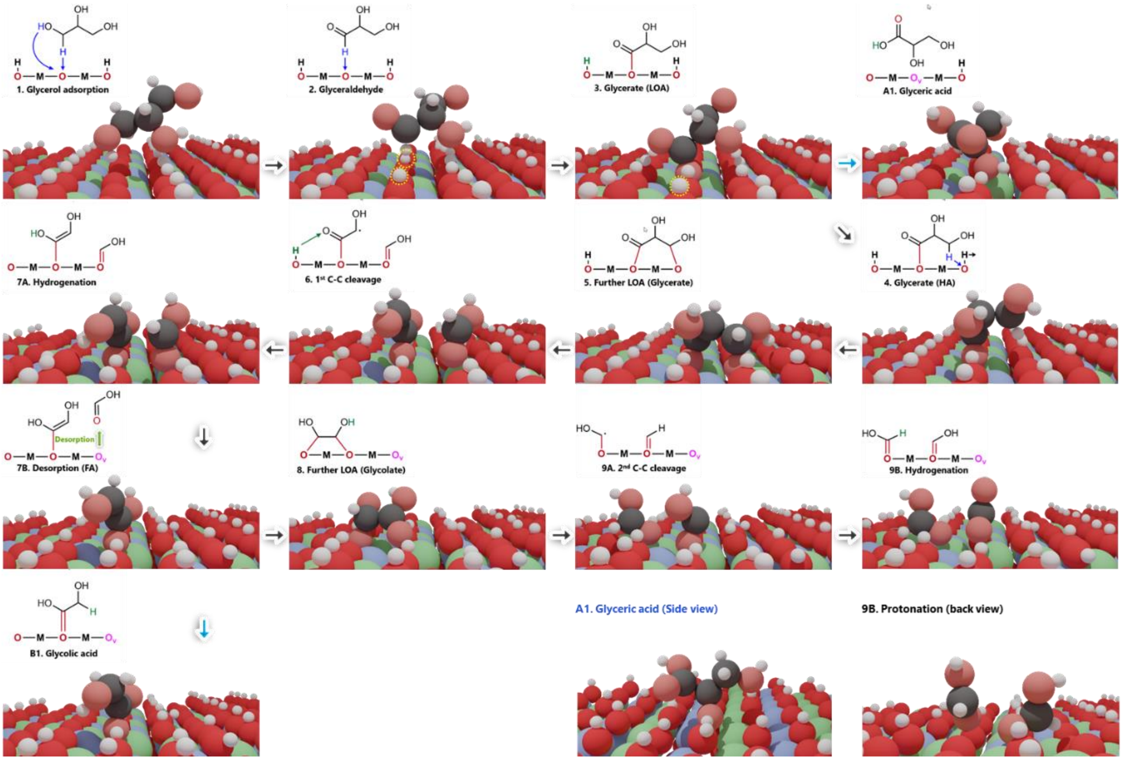

Supplementary Fig. 27. The configuration of the adsorbed intermediates on NiCo hydroxide. The H atom in dash circle is from the surface reaction intermediate during the dehydrogenation reaction.

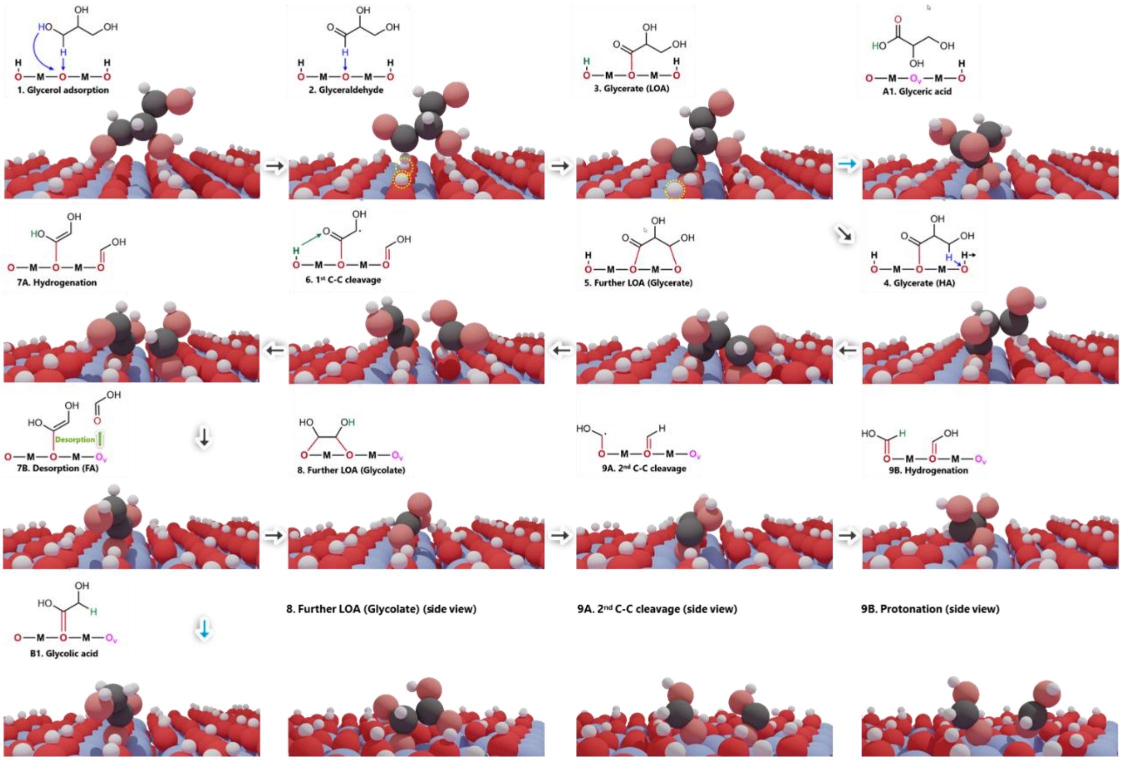

Supplementary Fig. 28. The configuration of the adsorbed intermediates on Co hydroxide. The H atom in dash circle is from the surface reaction intermediate during the dehydrogenation reaction.

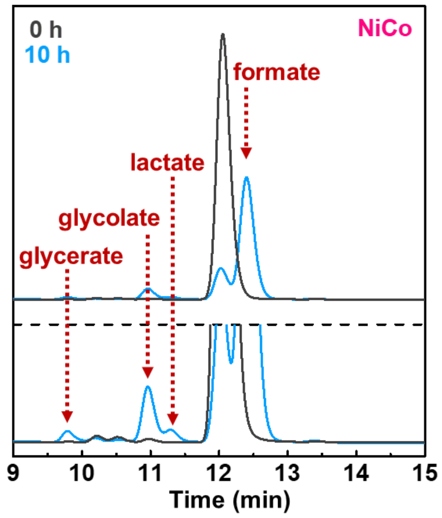

Supplementary Fig. 29. The HPLC chromatograms analysis of GOR products for NiCo hydroxide. The electrolysis experiment was carried out at a constant applied potential of 1.624 V (vs. RHE) for 10 h in 50 mL electrolyte of 1.0 M KOH with 0.1 M glycerol on NiCo hydroxide. The bottom panel is the enlarged figure.

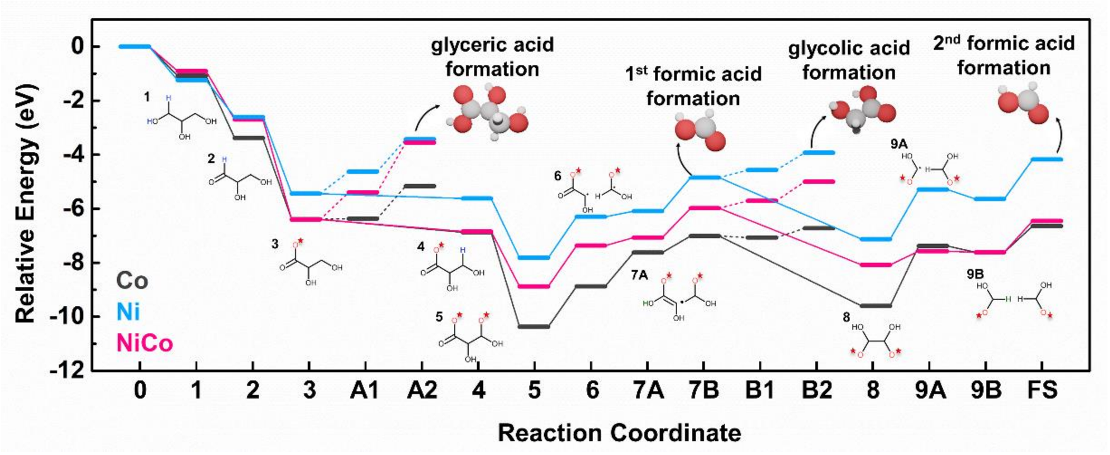

Supplementary Fig. 30. **Reaction energy calculations.** The calculated reaction energy profiles of glycerol oxidation reaction on Ni, NiCo and Co hydroxide. The numerical data are shown in **Supplementary Table 3**.

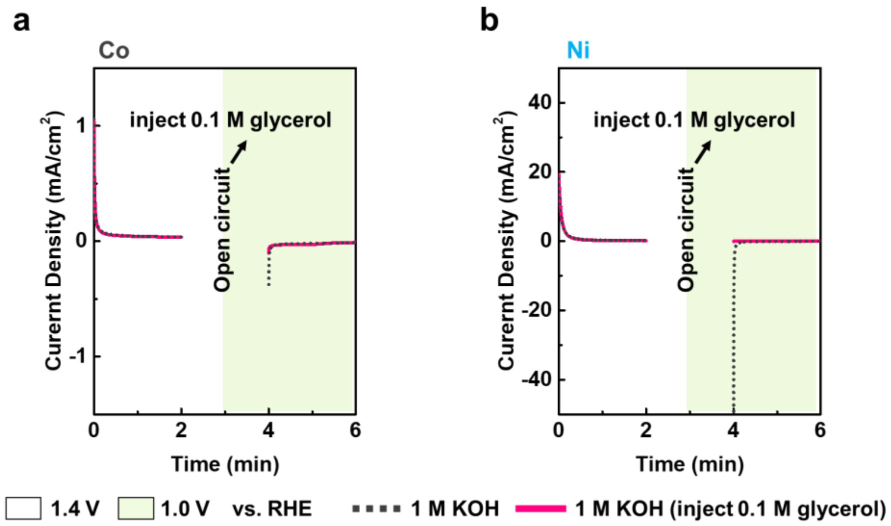

Supplementary Fig. 31. **Muti-potential step curves. a**, Co hydroxide. **b**, Ni hydroxide.

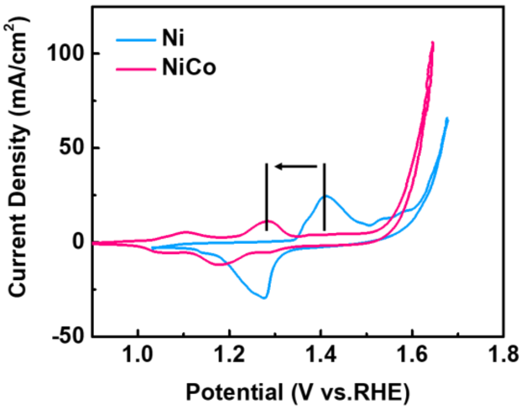

Supplementary Fig. 32. **The redox peak of Ni and NiCo hydroxide.** The CV curves of Ni and NiCo hydroxide.

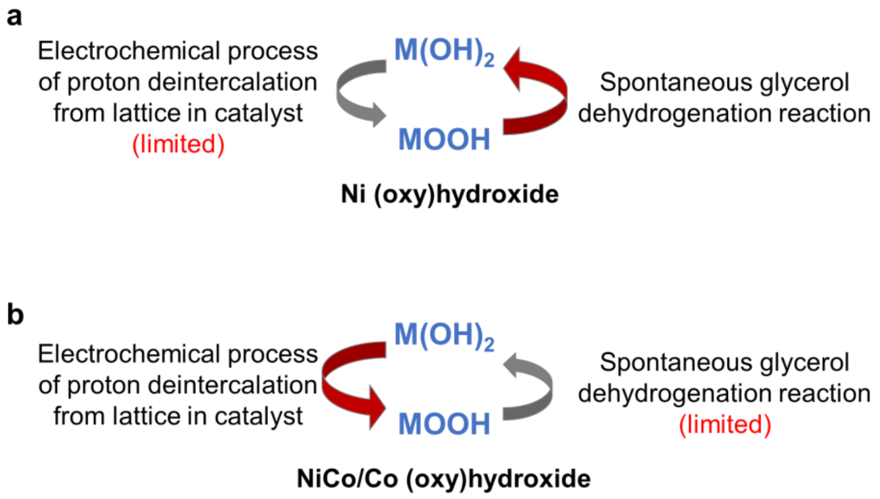

Supplementary Fig. 33. **Schematic representation of the catalyst transformation among $M(OH)_2$ and MOOH at applied potential during glycerol dehydrogenation step. a**, Ni hydroxide. **b**, NiCo and Co hydroxide.

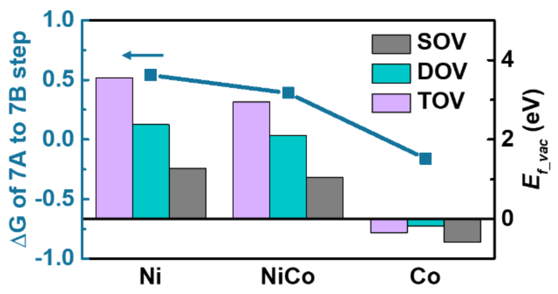

Supplementary Fig. 34. **Correlation between oxygen vacancy formation energy and product desorption free energy barrier.** The free energy barrier of the desorption step (7A→7B) and the energy for oxygen anion deintercalation to form single oxygen vacancy (SOV), double oxygen vacancies (DOV), and triple oxygen vacancies (TOV) in Co, NiCo, and Ni hydroxide.

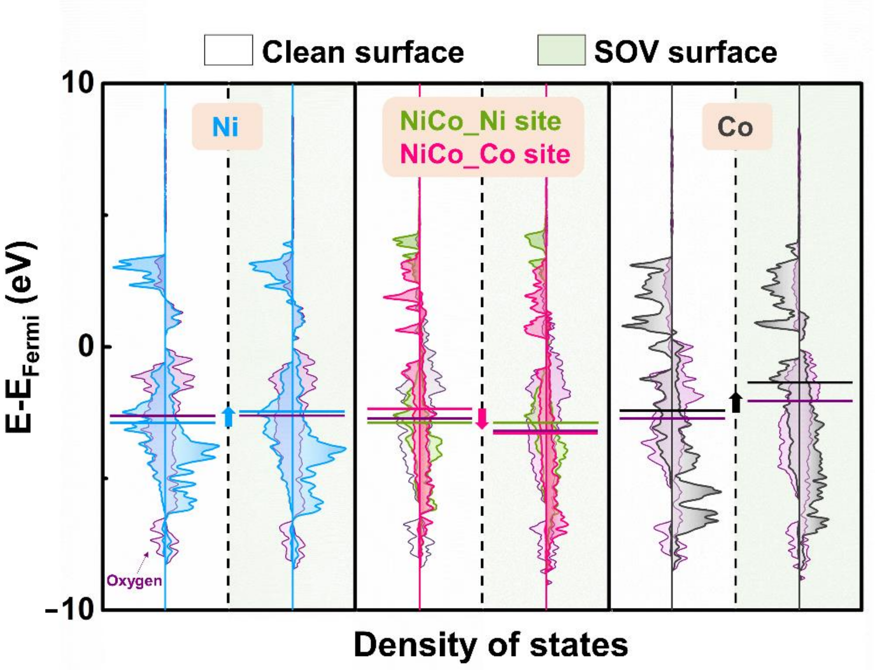

Supplementary Fig. 35. **Electronic structure calculations.** The density of state (DOS) of metal d band and oxygen p band of Ni, NiCo, and Co hydroxide before (white area) and after (green area) oxygen anion deintercalation. The lines indicate the corresponding band center.

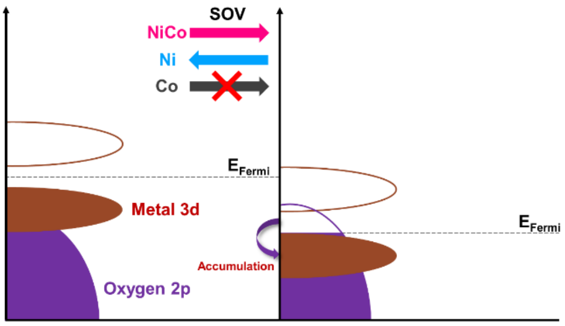

Supplementary Fig. 36. **Charge redistribution analysis.** The charge redistribution scheme owing to oxygen anion deintercalation from lattice in Ni, NiCo and Co hydroxide. The arrows indicate the change of band states when oxygen vacancy is formed.

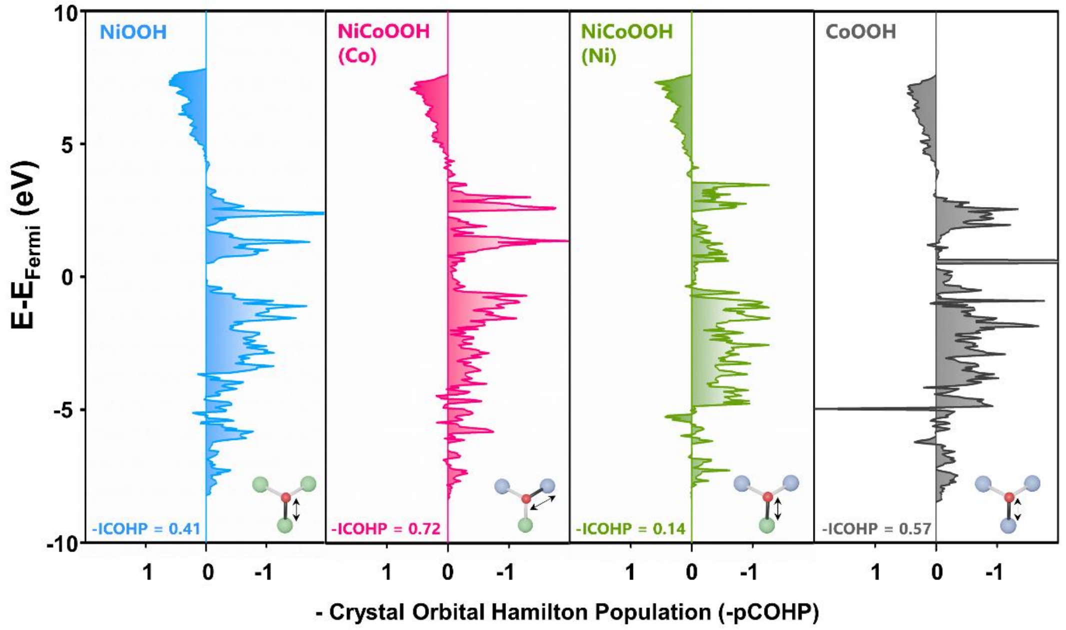

Supplementary Fig. 37. **Crystal orbital Hamiltonian population (COHP) calculations.** The - COHP and -integrated COHP (ICOHP) of metal-oxygen bond on clean surface.

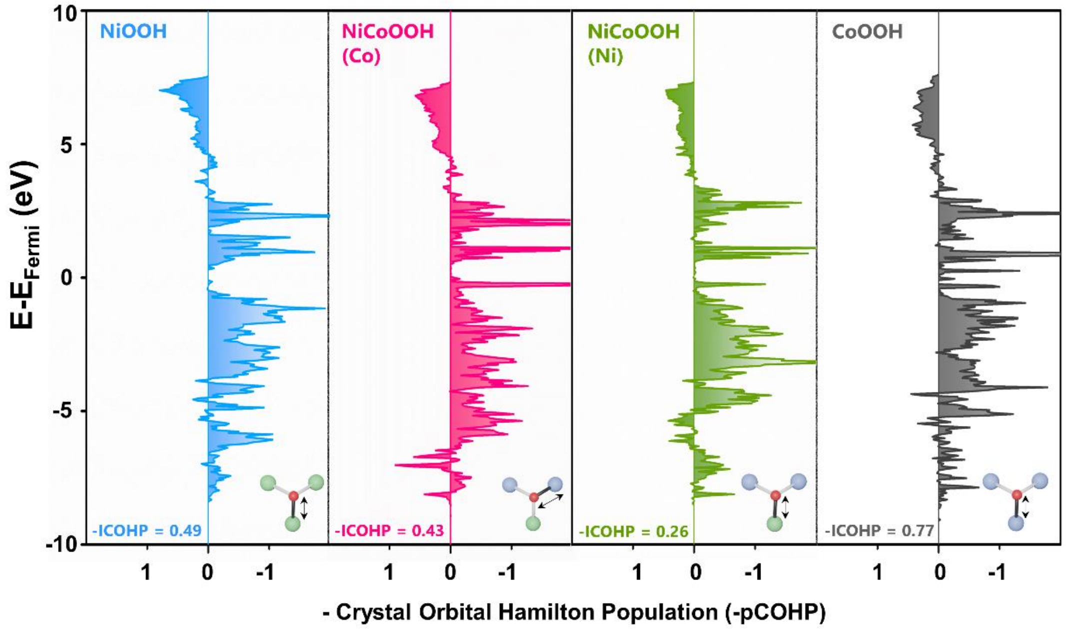

Supplementary Fig. 38. **Crystal orbital Hamiltonian population (COHP) calculations.** The -COHP and -integrated COHP (ICOHP) of metal-oxygen bond on SOV surface.

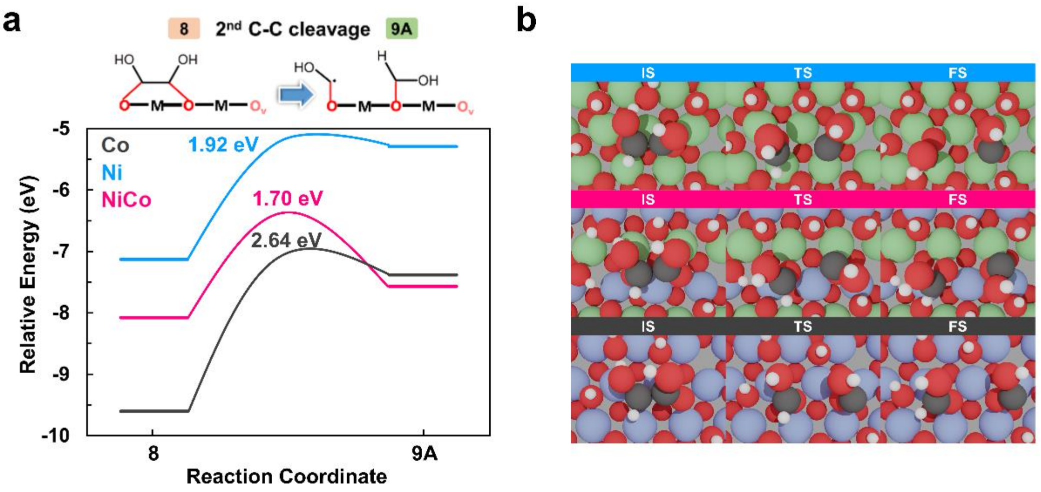

Supplementary Fig. 39. The calculation of activation energies of 2nd C-C cleavage step for Ni, NiCo and Co (oxy)hydroxides by transition state searching. a, The energy profile of 2nd C-C cleavage step including corresponding activation energies. **b**, Images of initial states (IS), transition states (TS), and final states (FS) of Ni, NiCo, and Co (oxy)hydroxides for 2nd C-C cleavage step.

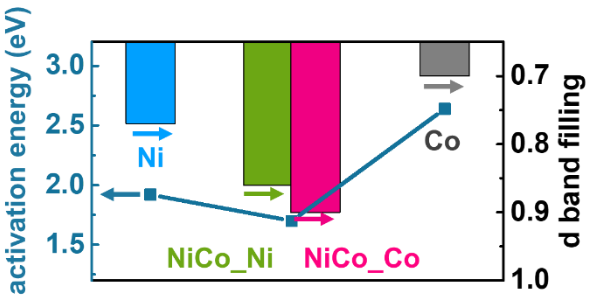

Supplementary Fig. 40. The correlation between activation energy and *d*-band filling. The activation energy of the 2nd C-C bond cleavage step (8→9A) and the *d*-band filling before 2nd C-C bond cleavage of Ni, NiCo, and Co hydroxide. NiCo_Ni and NiCo_Co represent the Ni sites and Co sites on NiCo hydroxide, respectively.

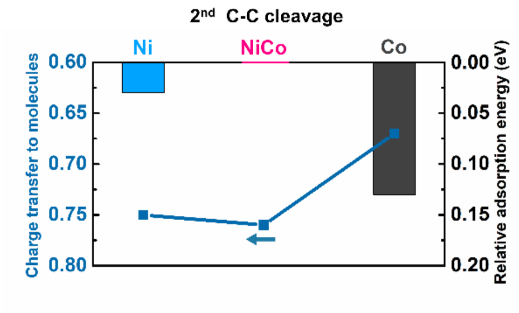

Supplementary Fig. 41. **The correlation between charge transfer and the adsorption energy.** The relative adsorption energy and bader charge analysis of cleaved molecules on Ni, NiCo and Co hydroxide after the 2nd C-C bond cleavages.

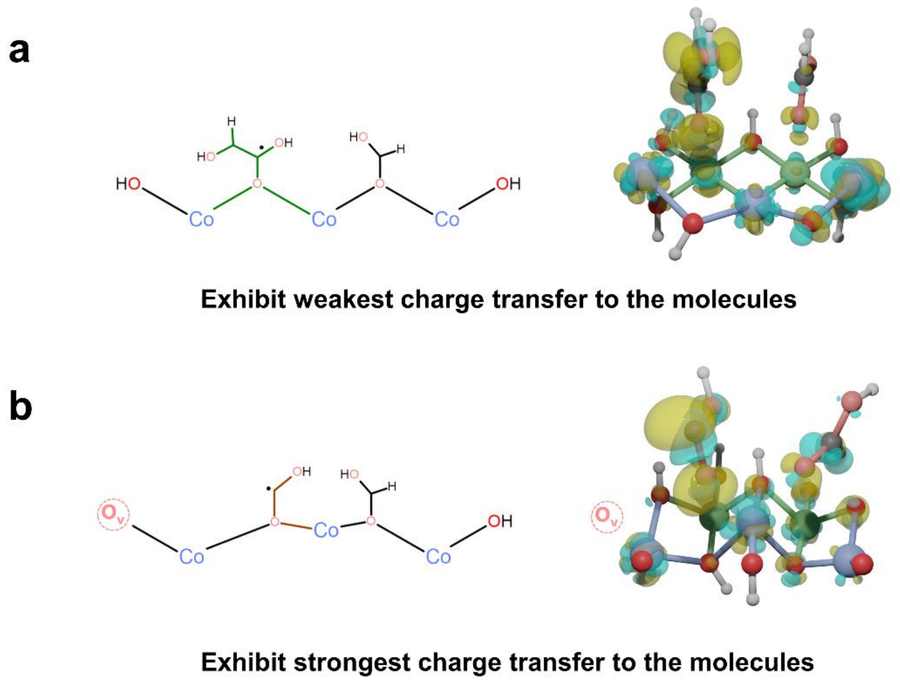

Supplementary Fig. 42. **The bader charge analysis of NiCo hydroxide. a**, The 1st C-C cleavage steps (without oxygen vacancy). **b**, The 2nd C-C cleavage step (with oxygen vacancy). The yellow area indicates the charge accumulation while cyan area indicates the charge depletion. The strength of charge transfer is defined by comparing other catalysts.

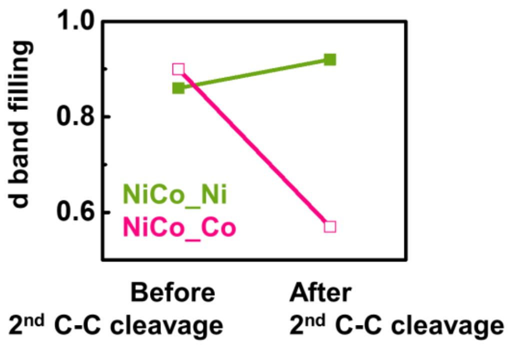

Supplementary Fig. 43. ***d*-band filling calculation.** The evolution of the *d*-band filling of Ni site and Co site on NiCo hydroxide before and after the 2nd C-C bond cleavage.

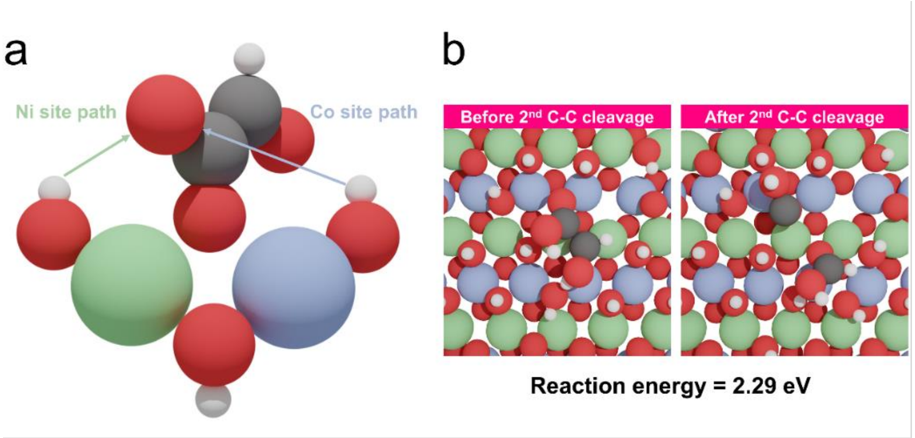

Supplementary Fig. 44. The reaction scheme of Ni site-based 2nd C-C cleavage step in NiCo (oxy)hydroxide and its corresponding reaction energy when 2nd C-C cleavage takes place in Ni site on NiCo (oxy)hydroxide. a, Possible hydrogenation schemes for the hydrogenation of glycolate. b, Structures of before and after 2nd C-C cleavage on Ni sites with the corresponding reaction energy.

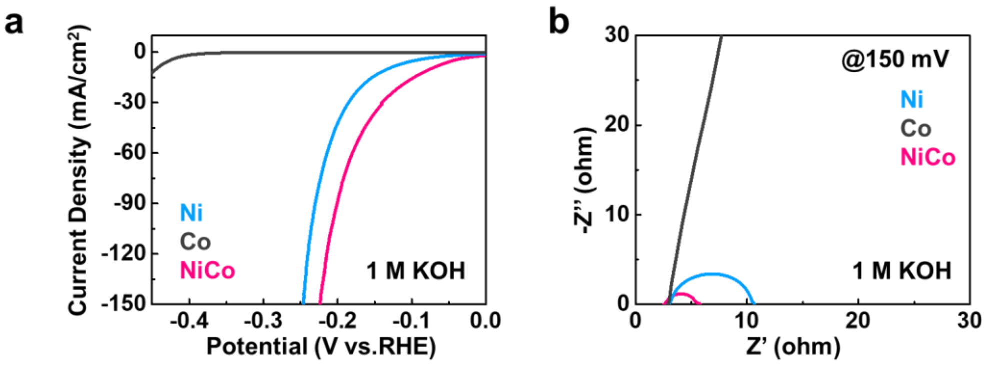

Supplementary Fig. 45. **HER performance of Ni, NiCo and Co hydroxide. a**, Linear sweep voltammetry curves. **b**, Electrochemical impedance spectroscopies.

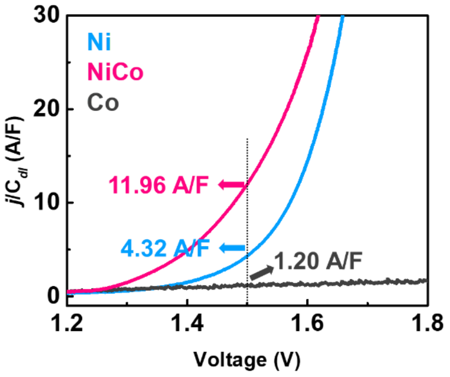

Supplementary Fig. 46. **Specific activity for overall electrolysis coupling HER and GOR.** LSV polarization curves normalized by double-layer capacitance.

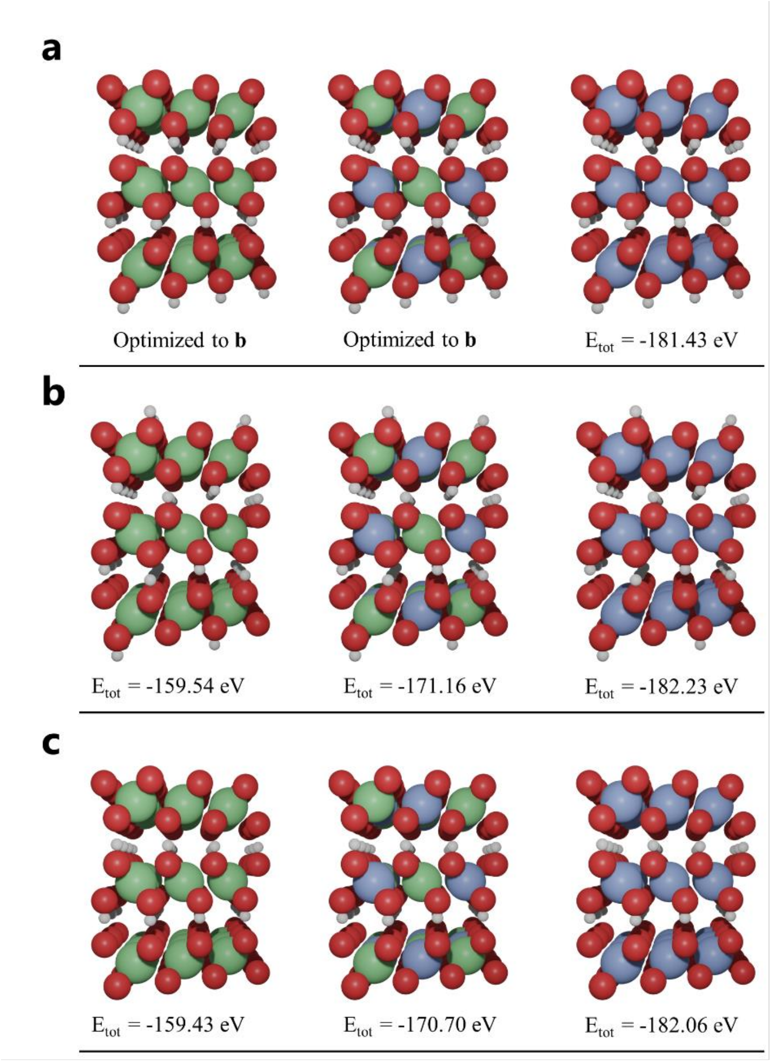

Supplementary Fig. 47. Configuration of bulk structure and corresponding total energies of Ni, NiCo, and Co hydroxide. a, Hydrogen atoms located at each bottom layers of oxygen. **b**, location of hydrogen atoms with chain-saw-like placement. **c**, hydrogen atoms located on only at the middle layer of bulk structure. Different bulk structures were composed of same number of atoms (metal: 8, oxygen :16, hydrogen: 8).

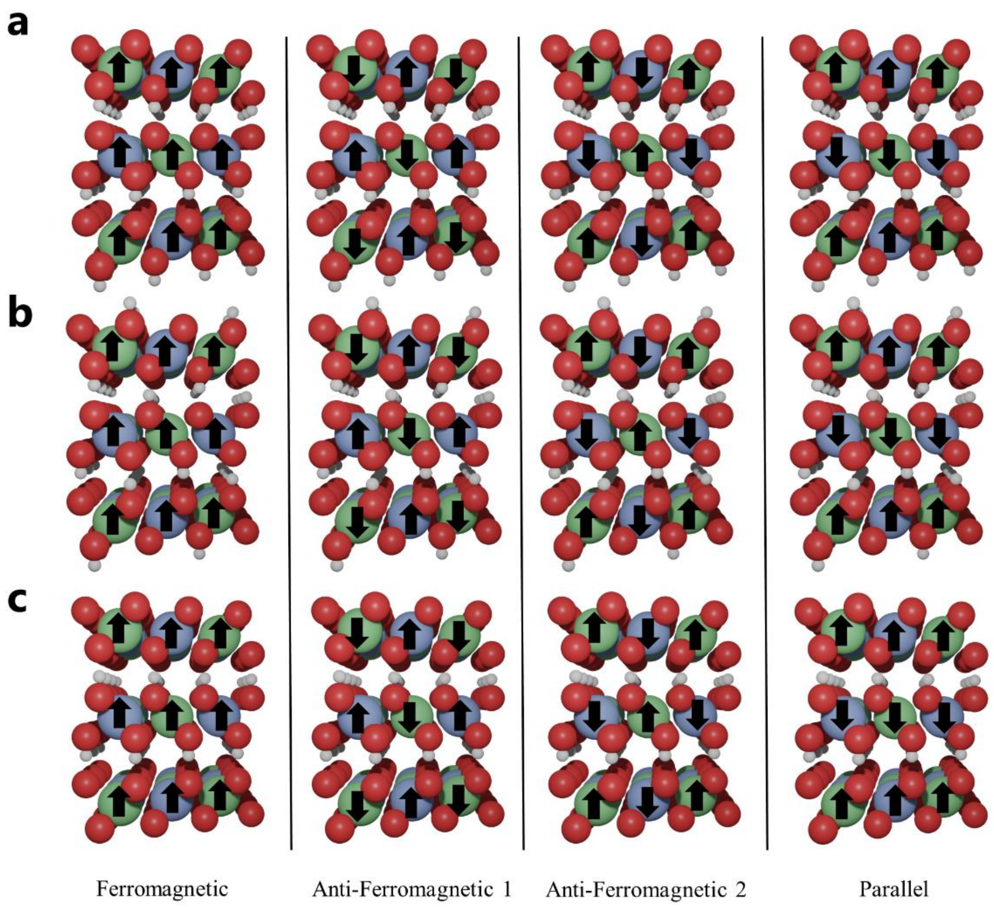

Supplementary Fig. 48. Possible initial spin configuration of bulk structures: a, Hydrogen atoms located at each bottom layers of oxygen, **b**, location of hydrogen atoms with chain-saw-like placement, and **c**, hydrogen atoms located at the middle layer of bulk structure.

Supplementary Tables

Supplementary Table 1. The loading mass of Ni and NiCo hydroxide on carbon cloths obtained from the inductively coupled plasma-optical emission spectrometry measurement.

loading mass (mg/cm^2)	Ni atom	Co atom	total
Ni hydroxide	0.848211	0.000148	0.848359
NiCo hydroxide	0.566325	0.275305	0.84163

Supplementary Table 2. The Gibbs free energy of each step in glycerol oxidation reaction.

Step	Reaction	Co (eV)	NiCo (eV)	Ni (eV)
0 → 1	Glycerol adsorption	- 0.37	- 0.04	- 0.46
1 → 2	Dehydrogenation (Glyceraldehyde)	- 2.92	- 2.56	- 2.00
2 → 3	Dehydrogenation + 1st Lattice Oxygen Attack (Glycerate)	- 3.14	- 3.93	- 3.00
3 → A1	Hydrogenation and O_v formation (Glyceric acid)	+ 0.23	+ 1.34	+ 1.03
A1 → A2	Desorption (Glyceric acid)	+ 0.54	+ 1.14	+ 0.50
3 → 4	Change configuration	- 0.42	- 0.32	- 0.22
4 → 5	Dehydration + 2nd Lattice Oxygen Attack	- 3.80	- 2.25	- 2.37
5 → 6	1st C-C cleavage	+ 1.32	+ 1.21	+ 1.36
6 → 7A	Hydrogenation	+ 1.69	+ 0.74	+ 0.54
7A → 7B	Desorption (**Formic acid**)	- 0.16	+ 0.39	+ 0.54
7B → B1	Hydrogenation (Glycolic acid)	+ 0.21	+ 0.65	+ 0.66
B1 → B2	Desorption (Glycolic acid)	- 0.23	+ 0.01	- 0.09
7B→ 8	3rd Lattice Oxygen Attack	- 2.38	- 1.91	- 2.07
8 → 9A	2nd C-C cleavage	+ 2.02	+ 0.37	+ 1.58
9A → 9B	Hydrogenation	+ 0.19	+ 0.21	+ 0.09
9B → FS	Desorption (**Formic acid**)	- 0.39	- 0.09	+ 0.10

Supplementary Table 3. The reaction energy of each step in glycerol oxidation reaction.

Step	Reaction	Co (eV)	NiCo (eV)	Ni (eV)
0 → 1	Glycerol adsorption	- 1.08	- 0.90	- 1.24
1 → 2	Dehydrogenation (Glyceraldehyde)	- 2.30	- 1.80	- 1.36
2 → 3	Dehydrogenation + 1st Lattice Oxygen Attack (Glycerate)	- 3.01	- 3.70	- 2.84
3 → A1	Hydrogenation and O_v formation (Glyceric acid)	+ 0.02	+ 1.00	+ 0.81
A1 → A2	Desorption (Glyceric acid)	+ 1.21	+ 1.85	+ 1.20
3 → 4	Change configuration	- 0.48	- 0.44	- 0.18
4 → 5	Dehydration + 2nd Lattice Oxygen Attack	- 3.50	- 2.04	- 2.20
5 → 6	1st C-C cleavage	+ 1.50	+ 1.52	+ 1.52
6 → 7A	Hydrogenation	+ 1.25	+ 0.29	+ 0.21
7A → 7B	Desorption (**Formic acid**)	+ 0.61	+ 1.09	+ 1.24
7B → B1	Hydrogenation (Glycolic acid)	- 0.06	+ 0.27	+ 0.28
B1 → B2	Desorption (Glycolic acid)	+ 0.35	+ 0.71	+ 0.64
7B→ 8	3rd Lattice Oxygen Attack	- 2.59	- 2.10	- 2.28
8 → 9A	2nd C-C cleavage	+ 2.22	+ 0.51	+ 1.84
9A → 9B	Hydrogenation	- 0.23	- 0.04	- 0.35
9B → FS	Desorption (**Formic acid**)	+ 0.97	+ 1.16	+ 1.46

Supplementary Table 4. The comparison of overall electrolysis performance coupling organic oxidation reactions with HER in aqueous media for NiCo hydroxide and other noble-metal-free electrocatalysts in recent reported literature.

catalyst	organics	product	Voltage for 10 mA/cm^2	Voltage for 100 mA/cm^2	stability	reference
NiCo hydroxide	0.1 M glycerol	formate	1.33 V	1.58 V	100 mA/cm^2, 110 h	This work
Ni-Mo-N/CFC	0.1 M glycerol	formate	1.36 V	-	10 mA/cm^2, 11 h	Nat. Commun., 2019, 10(1): 1-12. (*Ref*[1])
$Ni(OH)_2$/NF	0.5 M MeOH	formate	1.52 V	-	20 mA/cm^2, 15 h	Appl. Catal. B-Environ., 2021, 281: 119510. (*Ref*[2])
Ni_3N@C	10 mM HMF	FDCA	1.46 V	1.55 V (50 mA/cm^2)	-	Angew. Chem. Int. Ed., 2019, 131, 16042-16050. (*Ref*[3])
MoO_2-FeP@C	10 mM HMF	FDCA	1.486 V	1.7 V	-	Adv. Mater., 2020, 2000455. (*Ref*[4])
hp-Ni	10 mM benzyl alcohol	benzoic acid	1.50 V	1.66 V	-	ACS Catal., 2017, 7: 4564-4570. (*Ref*[5])
NiSe@NiO_x	10 mM HMF	FDCA	1.50 V	-	-	Appl. Catal. B-Environ., 2020, 261: 118235. (*Ref*[6])
Co-P/CF	50 mM HMF	FDCA	~1.4 V	-	-	ACS Energy Lett., 2016, 1: 386-390. (*Ref*[7])
Ni_3S_2/NF	10 mM HMF	FDCA	1.46 V	1.64 V	-	J. Am. Chem. Soc., 2016, 138: 13639-13646. (*Ref*[8])
Ni_2P NPA/NF	10 mM HMF	FDCA	1.44 V	1.58 V (50 mA/cm^2)	-	Angew. Chem. Int. Ed., 2016, 55: 9913-9917.

						(*Ref* [9])
Ni@C-250	0.5 M urea	CO_2, N_2	1.46 V	1.46 V	-	Catal. Sci. Technol., 2021, 11: 2480-2490. (*Ref* [10])
CNTs@Co/CoP	0.5 M glucose	gluconic acid, glucaric acid	1.42 V	-	10 mA/cm^2, 50 h	J. Mater. Chem. A, 2021, 9: 10893-10908. (*Ref* [11])
NF-G-Mn/CoP_x-NF	0.5 M urea	CO_2, N_2	1.41 V	-	1.4 V, 20h	Angew. Chem. Int. Ed., 2016, 128: 3868 -3872. (*Ref* [12])
Co_3O_4 NWs/CC ‖ CoP NWs/CC	40 mg/L triclosan	phenol, 1,2-dihydroxybenzene, and 2-phenoxyphenol	1.63 V	-	-	Mater. Chem. Front., 2018, 2: 323-330. (*Ref* [13])
Fe-Ni_3S_2/Ni foam	0.33 M urea	CO_2, N_2	1.46 V	~1.78 V	10 mA/cm^2, 22 h	J. Mater. Chem. A, 2018, 6: 4346-4353. (*Ref* [14])
$Ni(OH)_2$/CC	0.33 M urea	CO_2, N_2	1.44 V	-	70 mA/cm^2, 8 h	Inorg. Chem. Front., 2017, 4: 1120-1124. (*Ref* [15])
NC@$CuCo_2N_x$/CF	15 mM benzyl alcohol	benzaldehyde	1.55 V	-	10 mA/cm^2, 60 h	Adv. Funct. Mater., 2017: 1704169. (*Ref* [16])
$Co_{0.26}$-$Ni(OH)_2$ NPs/CF	0.5 M urea	CO_2, N_2	1.37 V	1.79 V	10 mA/cm^2, 38 h	J. Catal., 2020, 381: 454-46. (*Ref* [17])
CoMn/$CoMn_2O_4$	0.5 M urea	CO_2, N_2	1.51 V	1.68 V	100 mA/cm^2, 60000 s	Adv. Funct. Mater., 2020: 2000556. (*Ref* [18])
$Co(OH)_2$@HOS/CP	3 M methanol	formate	1.497 V	1.66 V (70 mA/cm^2)	10 mA/cm^2, 20 h	Adv. Funct. Mater., 2020: 1909610. (*Ref* [19])
CoOOH/Ni	10 mM BHMF	FDCA	1.481 V	~1.88 V	-	Appl. Catal. B-Environ., 2021,

						297: 120396 (*Ref*[20])
E-CoAl-LDH-NSA	10 mM HMF	FDCA	~1.50 V	1.74 V (50 mA/cm^2)	-	Appl. Catal. B-Environ., 2021, 299: 120669. (*Ref*[21])
O-NiMoP/NF	0.5 M urea	CO_2, N_2	1.36 V	1.55 V (50 mA/cm^2)	-	Adv. Funct. Mater., 2021, 31(43): 2104951. (*Ref*[22])
$Ni_2Fe(CN)_6$	0.33 M urea	CO_2, N_2	1.38 V	1.50 V	-	Nat. Energy, 2021, 6(9): 904-912. (*Ref*[23])
P-$CoNi_2S_4$	0.5 M urea	CO_2, N_2	1.402 V	~1.52 V	10 mA/cm^2, 100 h	Angew. Chem. In. Ed., 2021, 60(42): 22885-22891. (*Ref*[24])
Anode: $Ni(OH)_2$<br>Cathode: $NiCrO_x$/Ni	0.1 M glycerol	formate	~1.42 V	-	-	Angew. Chem. In. Ed., 2021. (*Ref*[25])
N-CoO_x	1.0 M glycerol	formate	1.59 V	-	-	J. Mater. Chem. A, 2021, 9(35): 19975-19983. (*Ref*[26])
V-Ni_3N/NF	0.5 M urea	CO_2, N_2	1.416 V	1.543 V	10 mA/cm^2, 200 h	J. Mater. Chem. A, 2021, 9(7): 4159-4166. (*Ref*[27])

Supplementary Table 5. Total energy of bulk structure with different initial spin configurations.

	**FM**	**AFM1**	**AFM2**	**PS**
Co (oxy)hydroxide structure (a)	Optimized to (b) structure	- 181.43 eV	-	- 181.38 eV
NiCo (oxy)hydroxide structure (a)	Optimized to (b) structure	Optimized to (b) structure	Optimized to (b) structure	Optimized to (b) structure
Ni (oxy)hydroxide structure (a)	Optimized to (b) structure	Optimized to (b) structure	-	Optimized to (b) structure
Co (oxy)hydroxide structure (b)	- 182.06 eV	- 182.23 eV	-	- 181.73 eV
NiCo (oxy)hydroxide structure (b)	- 170.73 eV	- 170.88 eV	- 170.88 eV	- 171.16 eV
Ni (oxy)hydroxide structure (b)	- 159.54 eV	- 159.37 eV	-	- 159.39 eV
Co (oxy)hydroxide structure (c)	- 182.06 eV	Optimized to (b) structure	-	- 181.87 eV
NiCo (oxy)hydroxide structure (c)	Optimized to (b) structure	Optimized to (b) structure	Optimized to (b) structure	- 170.70 eV
Ni (oxy)hydroxide structure (c)	- 159.43 eV	- 159.41 eV	-	- 159.40 eV

Supplementary Table 6. The calculated band-gap energies of bulk and surface models of Ni, NiCo, and Co hydroxide.

	**Bulk model**	**Surface model**
Cobalt	1.67 eV (direct band-gap)	Metallic
Nickel/Cobalt	2.66 eV (direct band-gap)	0.01 eV (direct band-gap)
Nickel	3.33 eV (direct band-gap)	0.61 eV (direct band-gap)

Supplementary Notes

Supplementary note 1: Materials synthesis and characterization

Ni hydroxide was prepared on carbon cloths by electrodeposition, and then Co was doped in Ni hydroxide to obtain NiCo hydroxide via cation exchange reaction (**Supplementary Fig. 1**). Co hydroxide was also deposited on carbon cloths by chemical bath for comparison. Both Ni hydroxide and NiCo hydroxide are with a nanosheet structure (**Supplementary Fig. 2-3**), while Co hydroxide is a uniform film with nanoparticles on the surface coating on carbon cloths (**Supplementary Fig. 4**). High-resolution transmission electron microscopy (HRTEM), selected-area electron diffraction (SAED), energy-dispersive X-ray spectroscopy (EDS), X-ray diffraction (XRD) pattern and X-ray photoelectron spectroscopy (XPS) were measured to identify the detailed chemical composition and crystal structure of the obtained samples.

The Ni 2p XPS spectra of NiCo hydroxide is same as that of Ni hydroxide, which are consist of a spin-orbit doublet of Ni $2p_{1/2}$ and Ni $2p_{3/2}$ and two satellite peaks (denoted as "Sat.") (**Supplementary Fig. 5a**). The doublet of Ni $2p_{1/2}$ and Ni $2p_{3/2}$ at 873.9 eV and 856.3 eV can be assigned to Ni hydroxide[28-30]. For the Co 2p XPS spectra, the satellite peak assigning to Co^{2+} for NiCo hydroxide show lower intensity compared to that of Co hydroxide (**Supplementary Fig. 5b**), which indicates that cobalt in NiCo hydroxide is with higher valence.[31] In addition, the ratio of Ni to Co in NiCo hydroxide was calculated to be 1.26 based on the area of Ni 2p and Co 2p XPS spectra. The EDS mapping revealed the uniform distribution of Ni, Co and O elements in NiCo hydroxide (**Supplementary Fig. 6**). It is noted that only the characteristic diffraction peaks of

carbon cloths substrate and nickel framework were observed in XRD patterns of all samples due to the low crystallization of hydroxides on the surface (**Supplementary Fig. 7**).

For Ni and Co hydroxide, diffraction rings of (102) and (101) plane of hydroxide can be identified in the SAED patterns (**Supplementary Fig. 8-9**). For NiCo hydroxide, diffraction rings of (012) and (110) plane of layered double-metal hydroxide can be identified in the SAED pattern (**Supplementary Fig. 10**). The HRTEM images of NiCo hydroxide showed the lattice fringes with the spacing between two adjacent lattice planes of 0.26 nm, assigning to the (012) plane of double-metal hydroxide, which is consistent with previous works[32-34] (**Supplementary Fig. 11**). Consistently, the corresponding fast Fourier transform (FFT) pattern exhibited the clear diffraction rings of (012) and (110) of NiCo-based double-metal hydroxide (**Supplementary Fig. 11c**). To quantify the loading mass of catalysts on carbon cloths, the inductively coupled plasma-optical emission spectrometry (ICP-OES) measurement was carried out. The ICP-OES results demonstrate that the total loading mass of Ni hydroxide and NiCo hydroxide is similar (~0.85 mg/cm^2) (**Supplementary Table 1**), further proving that Co was doped in $Ni(OH)_2$ by cation exchange.

Supplementary note 2: turnover frequency (TOF) calculation

The TOF of formate production on Ni, NiCo and Co hydroxide was calculated by the following equation:

$$TOF = \frac{j}{\alpha NF} \times FE_{formate} \quad (1)$$

where j is the current density at a given potential obtained from electrochemical measurement; α is the number of electron transfer to form one formate molecule; N is the number of active sites; F is the Faraday constant, equaling to 96485 C/mol; $FE_{formate}$ is the Faradaic efficiency toward formate. According to the equation (27), the $FE_{formate}$ for Ni, NiCo, and Co hydroxide were calculated to be 100%, 100%, and 57.7%, respectively.

The number of active sites per unit area was estimated by the follow equation:

$$N = \left(\frac{number\ of\ active\ atom/unit\ cell}{volumn\ of\ unit\ cell}\right)^{\frac{2}{3}} \times A_{ECSA} \tag{2}$$

where A_{ECSA} is the electrochemical surface area, which estimated by the double-layer capacitance (C_{dl}):

$$A_{ECSA}(NiOOH) = \frac{3.100\ mF\ cm^{-2}}{40\ \mu F\ cm^{-2}\ per\ cm^2_{ECSA}} = 77.5\ cm^2_{ECSA} \tag{3}$$

$$A_{ECSA}(NiCoOOH) = \frac{4.475\ mF\ cm^{-2}}{40\ \mu F\ cm^{-2}\ per\ cm^2_{ECSA}} = 111.875\ cm^2_{ECSA} \tag{4}$$

$$A_{ECSA}(CoOOH) = \frac{0.910\ mF\ cm^{-2}}{40\ \mu F\ cm^{-2}\ per\ cm^2_{ECSA}} = 22.75\ cm^2_{ECSA} \tag{5}$$

In our work, the computed values of optimized lattice parameters of (oxy)hydroxide models with chainsaw-like arrangement of hydrogen were as follow: CoOOH (a = b = 6.091 Å; c = 9.124 Å), NiCoOOH (a = b = 5.948 Å; c = 9.022 Å), and NiOOH (a = b = 5.868 Å; c = 9.063 Å), which contain 8 metal atoms, 16 oxygen atoms (half of them are exposed), and 8 hydrogen atoms. The exposed oxygen atoms act as the active sites for glycerol oxidation. Therefore, the number of active sites of Ni, NiCo, and Co (oxy)hydroxide were calculated to be:

$$N(NiOOH) = (\frac{8\ atoms/unit\ cell}{5.868\ \text{Å} \times 5.868\ \text{Å} \times 9.063\ \text{Å}})^{\frac{2}{3}} \times 77.5\ cm_{ECSA}^{2} = 6.74 \times 10^{16} \quad (6)$$

$$N(NiCoOOH) = (\frac{8\ atoms/unit\ cell}{5.948\ \text{Å} \times 5.948\ \text{Å} \times 9.022\ \text{Å}})^{\frac{2}{3}} \times 111.875\ cm_{ECSA}^{2} = 9.58 \times 10^{16} \quad (7)$$

$$N(CoOOH) = (\frac{8\ atoms/unit\ cell}{6.091\ \text{Å} \times 6.091\ \text{Å} \times 9.124\ \text{Å}})^{\frac{2}{3}} \times 22.75\ cm_{ECSA}^{2} = 1.87 \times 10^{16} \quad (8)$$

Therefore, the formula for calculating TOF can be written in the following format:

$$TOF(NiOOH) = \frac{j(A\ cm^{-2}) \times A(cm^{2}) \times 6.02 \times 10^{23}(mol^{-1}) \times 100\%}{\frac{8}{3} \times 6.74 \times 10^{16} \times 96485\ (A\ s\ mol^{-1})} \quad (9)$$

$$TOF(NiCoOOH) = \frac{j(A\ cm^{-2}) \times A(cm^{2}) \times 6.02 \times 10^{23}(mol^{-1}) \times 100\%}{\frac{8}{3} \times 9.58 \times 10^{16} \times 96485(A\ s\ mol^{-1})} \quad (10)$$

$$TOF(CoOOH) = \frac{j(A\ cm^{-2}) \times A(cm^{2}) \times 6.02 \times 10^{23}(mol^{-1}) \times 57.7\%}{\frac{8}{3} \times 1.87 \times 10^{16} \times 96485(A\ s\ mol^{-1})} \quad (11)$$

Specifically, the TOF of all samples at 1.4 V (vs. RHE) can be calculated to be:

$$TOF(NiOOH) = \frac{1.031 \times 10^{-1} \times 6.02 \times 10^{23} \times 100\%}{\frac{8}{3} \times 6.74 \times 10^{16} \times 96485} = 3.579\ s^{-1} \quad (12)$$

$$TOF(NiCoOOH) = \frac{1.981 \times 10^{-1} \times 6.02 \times 10^{23} \times 100\%}{\frac{8}{3} \times 9.58 \times 10^{16} \times 96485} = 4.838\ s^{-1} \quad (13)$$

$$TOF(CoOOH) = \frac{1.606 \times 10^{-3} \times 6.02 \times 10^{23} \times 57.7\%}{\frac{8}{3} \times 1.87 \times 10^{16} \times 96485} = 0.116\ s^{-1} \quad (14)$$

Supplementary note 3: Product analysis.

The glycerol oxidation products were determined by high-performance liquid chromatography (HPLC, Agilent 1260), which was equipped with a Xtimate Sugar-H column and a differential refractive index detector. Long-term electrolysis reactions of

glycerol oxidation were carried out at a constant potential of 1.624 V (vs. RHE) by chronoamperometry in 50 mL electrolyte of 1 M KOH with 0.1 M glycerol. 1.0 mL electrolyte was extracted every 2 h for Ni hydroxide and NiCo hydroxide and every 10 h for Co hydroxide, and then was diluted with 1.0 mL 0.51 M H_2SO_4 solution to adjust the pH below 7.0. 20 μL diluted solution was injected into the column. 0.27 mL H_2SO_4 diluted in 1000 mL H_2O was used as eluent with a constant flow rate of 0.6 mL/min. All the electrolysis experiments were carried out at room temperature and all the HPLC measurements were performed with a column temperature of 65 ºC.

The reaction products were also collected online during the LSV measurement, and their compositions were quantified by ex-situ HPLC measurement[35, 36]. The LSV measurement was carried out with a scan rate of 1 mV/s in a solution of 1.0 M KOH with 0.1 M glycerol. Aliquots of 0.3 mL electrolyte were collected with a flow rate of 3 μL/s. Thus, each collected sample represents the products of GOR generated in an interval of 100 mV.

The composition of electrolyte after glycerol oxidation was identified based on the retention times of HPLC elution peaks of the individual standard sample. The product concentration was determined by the calibration curves of standard solutions with given concentrations. The glycerol conversion ($\eta_{glycerol}$), product yields ($Y_{glycerate}$, $Y_{glycolate}$, and $Y_{formate}$, respectively), and product selectivity ($S_{glycerate}$, $S_{glycolate}$, and $S_{formate}$, respectively) based on carbon balance were calculated by the following equations:

$$\eta_{glycerol} = \frac{C_{0,glycerol} - C_{glycerol}}{C_{0,glycerol}} \times 100\% \tag{15}$$

$$Y_{glycerate} = \frac{C_{glycerate}}{C_{0,glycerol}} \times 100\% \tag{16}$$

$$Y_{glycolate} = \frac{C_{glycolate} \times \frac{2}{3}}{C_{0,glycerol}} \times 100\% \tag{17}$$

$$Y_{formate} = \frac{C_{formate} \times \frac{1}{3}}{C_{0,glycerol}} \times 100\% \tag{18}$$

$$S_{glycerate} = \frac{C_{glycerate}}{C_{0,glycerol} - C_{glycerol}} \times 100\% \tag{19}$$

$$S_{glycolate} = \frac{C_{glycolate} \times \frac{2}{3}}{C_{0,glycerol} - C_{glycerol}} \times 100\% \tag{20}$$

$$S_{formate} = \frac{C_{formate} \times \frac{1}{3}}{C_{0,glycerol} - C_{glycerol}} \times 100\% \tag{21}$$

where the $C_{0,glycerol}$ and $C_{glycerol}$ are the initial and final concentration of glycerol, respectively; $C_{glycerate}$, $C_{glycolate}$, and $C_{formate}$ are the final concentrations of glycerate, glycolate, and formate, respectively.

The Faradaic efficiency calculations of the glycerol oxidation production are based on the following balance half-reactions:

$C_3H_8O_3$ (glycerol) + 5 OH^- → $C_3H_5O_4^-$ (glycerate) + 4 H_2O + 4 e^- (22)

$C_3H_8O_3$ (glycerol) + $\frac{13}{2}$ OH^- →$\frac{3}{2}$ $C_2H_3O_3^-$ (glycolate) + 5 H2O + 5 e^- (23)

$C_3H_8O_3$ (glycerol) + 11 OH^- → 3 $HCOO^-$ (formate) + 8 H_2O + 8 e^- (24)

The corresponding Faradaic efficiencies (FE) toward glycerate, glycolate, and formate are calculated based on the following equations:

$$FE_{glycerate} = \frac{C_{glycerate} \times 4}{Q_{total}} \times V \times F \times 100\% \tag{25}$$

$$FE_{glycolate} = \frac{C_{glycolate} \times \frac{2}{3} \times 5}{Q_{total}} \times V \times F \times 100\% \tag{26}$$

$$FE_{formate} = \frac{C_{formate} \times \frac{1}{3} \times 8}{Q_{total}} \times V \times F \times 100\% \tag{27}$$

where $C_{glycerate}$, $C_{glycolate}$, and $C_{formate}$ are the final concentrations of glycerate, glycolate, and formate, respectively; *V* is the volume of the electrolyte solution; *F* is the Faraday's constant (96485 C/mol); Q_{total} is the total charge passed.

Supplementary note 4: Reaction pathway

Prior to the GOR, the hydroxide went through an electrochemical process of proton deintercalation from lattice to form oxyhydroxide with exposed lattice oxygen sites (IS→0). These exposed lattice oxygen site then act as the active sites for glycerol adsorption (0→1) (**Supplementary note 5**). The protons of hydroxyl on the adsorbed glycerol molecule transfer to the catalyst surface to form glyceraldehyde (1→2). It is reported that there is an equilibrium between glyceraldehyde and dihydroxyacetone (DHA), which can convert to lactate in alkaline media by Cannizzaro reaction[37]. In our work, the content of lactate in products in rather small compared to the main product (formate) (**Supplementary Fig. 29**), which is negligible. Therefore, we proposed the pathway that glyceraldehyde undergoes the further dehydrogenation and the 1st lattice oxygen attack (2→3) to form glycerate intermediate (*OOC-CHOH-CH_2OH) for further oxidation.

The glycerate intermediate (*OOC-CHOH-CH_2OH) then goes two different reaction pathways to either glyceric acid production (3→A1→A2) or further oxidization (3→4→5). For glyceric acid production, the glycerate intermediate accepts

protons and desorbs from catalyst surface with an oxygen vacancy formation (3→A1→A2). For further oxidation pathway, the glycerate intermediate changes its adsorbed configuration with proton transfer to the neighboring lattice oxygen site, and then go through dehydrogenation process and the 2nd lattice oxygen attack (3→4→5) to form active C3 intermediate (*OOC-CH_2OH-COHO*). The active C3 intermediate then goes through the 1st C-C bond cleavage to form C2 (*OOC-*CHOH) and C1 (*O*CHOH) intermediates (5→6). The C2 intermediate accepts the proton from catalyst surface, while the C1 intermediate desorbs from lattice to form formate and leaves an oxygen vacancy on the catalyst surface (6→7A→7B).

The C2 intermediate then can goes towards two different reaction pathways for either glycolic acid production (7B→B1→B2) or further oxidation (7B→8). For glycolic acid production, the C2 intermediate accepts protons and desorbed from catalyst surface with an oxygen vacancy formation (7B→B1→B2). For further oxidation, the C2 intermediate accepts proton and undergoes the 3rd lattice oxygen attack (7B→8). Subsequently, the glycolate intermediate subjects to the 2nd C-C cleavage to form two C1 intermediates (8→9A), which then accept protons and desorb from catalyst surface to form two formic acid molecules with two oxygen vacancy formation on the catalyst surface(9A→9B→FS). Finally, the oxygen vacancies formed during product desorption are refilled by OH^- to refresh the surface (FS→IS).

Supplementary note 5: active site of glycerol adsorption

To identify the active site of glycerol adsorption, we have conducted the

calculations of the glycerol adsorption directly to the metallic site through OH for Ni, NiCo and Co hydroxides. As shown in **Supplementary Fig. 25**, the calculated results indicate that OH of glycerol is unstable in its attack on the metallic site of the surface. The unoptimized initial configurations that were modelled to attack the metallic site were spontaneously optimized to have configurations with OH to oxygen sites on the surface rather than the OH to the metal sites. This clearly shows that the glycerol is more favorable to be adsorbed on the surface through oxygen sites rather than the metal sites.

Supplementary note 6: Gibbs free energy calculation

Detailed information about the computational method used for determining Gibbs free energy and the environmental effects that were considered were described as the follows.

The Gibbs free energy (△G) of glycerol oxidation on Ni, NiCo, and Co hydroxides was calculated by correcting the obtained total energy with zero-point energy and entropy. The calculation of Gibbs free energy was modeled using computational equation (28):

$$\Delta G = \Delta E + \Delta ZPE - T\Delta S + eU_{NHE} + \Delta G_{pH} \quad (28)$$

where ΔE is the total energy difference, ΔZPE is the difference of zero-point energy, ΔS is the change of entropy, and T is the environment temperature. The eU_{NHE} denotes the contribution of electrode potential versus normal hydrogen electrode (NHE) with elementary charge e and G_{pH} term denotes the contribution of pH. By introducing

the U_{RHE} term, which is the potential versus reversible hydrogen electrode (RHE), the equation (28) can further be converted to equation (29), where pH contribution included in the U_{RHE} term reflects the pH dependence.

$$\Delta G = \Delta E + \Delta ZPE - T\Delta S + eU_{RHE} \tag{29}$$

In our calculation, the calculation of Gibbs free energy was performed to compare the catalytic activity of Ni, NiCo, and Co hydroxides for the glycerol oxidation by using equation (29) with T = 298K and U_{RHE} = 0V. Moreover, calculated Gibbs free energies were referenced to glycerol, glyceric acid, glycolic acid, and formic acid at 1 atm of fugacity, and the entropy and vibrational frequency were calculated using harmonic approximation. As our study is focused on comparing intrinsic activity of glycerol oxidation over Ni, NiCo, and Co hydroxides with identical surface structures, reaction intermediates and products, the solvation effect was neglected from Gibbs free energy calculation to avoid complexity. The Gibbs free energy agrees with the calculated reaction energy in terms of catalytic activity trend. The **Figure 2b** and **Supplementary Table 2** show the Gibbs free energy of each reaction step."

Supplementary note 7: Oxygen vacancies and redistribution of electrons

The redistribution of electrons induced by oxygen anion deintercalation from lattice is further confirmed by density of state (DOS) and crystal orbital Hamilton population (COHP) calculations on surface with and without oxygen vacancy. The band centers of metal *d* band and oxygen *p* band of Ni, NiCo and Co hydroxide were denoted on density of states plot (**Supplementary Fig. 35**). On clean surface without oxygen

vacancy, the d band center of Ni hydroxide was positioned below its oxygen p band center while the d band centers of NiCo and Co hydroxides were positioned above their oxygen p band centers. However, the presence of an oxygen vacancy of the surface can cause these band centers to shift and give different orders. The position of d band center on Ni hydroxide was up-shifted to be placed above the oxygen p band center with presence of an oxygen vacancy and the order of band centers was changed from fermi level → oxygen p band center → metal d band center to fermi level → metal d band center → oxygen p band center. On Co hydroxide, the formation of oxygen vacancy did not change the order of band centers since both of metal d band and oxygen p band centers were upshifted simultaneously towards the fermi level. However, NiCo hydroxide shows opposite trend to Ni hydroxide. The d band center of Co site on NiCo hydroxide was significantly downshifted when oxygen vacancy formed, resulting in the d band center of Co being located below the oxygen p band center. According to lattice oxygen activation scheme (**Supplementary Fig. 36**), entering of metal 3*d* band to the oxygen p band indicates the enhancement of charge transfer towards the metal sites.[38] As a result, the obtained band centers of Ni, NiCo, and Co hydroxide on surface with and without oxygen vacancy suggest that when an oxygen vacancy is formed, Ni hydroxide loses its tendency to accumulate the charges towards the metal site, whereas the presence of an oxygen vacancy leads to an increase in charge accumulation on Co site on NiCo hydroxide. Furthermore, there are no significant changes in the order of band centers in Co hydroxide, indicating that there is no major alteration in the direction of charge transfer between metal and oxygen.

In addition, COHP exhibits the same trends as the density of states results. As an extension of density of state study, COHP calculations have been performed to investigate the bonding and anti-bonding states of the model system. The negative and positive -COHP below the fermi level represent the occupied anti-bonding and bonding state, respectively. (**Supplementary Fig. 37-38**). Moreover, integrated COHP (ICOHP) value was obtained by integral of COHP from negative infinity to fermi level. The -ICOHP values were then used to quantify the bond strength of chosen metal-oxygen bonds.[39] The higher the value of -ICOHP, the stronger bond strength of corresponding bond. Among the number of different metal-oxygen bond on surfaces, the bonds with the highest value of -ICOHP were taken for Ni hydroxide and Co hydroxide. Interestingly, the position of the bonds where -ICOHP values are greatest was determined to be on the exact same site for both Ni hydroxide and Co hydroxide. Since the reaction was mainly proceeded on the Co site oxygens, the -ICOHP values were computed on the metal site to the oxygen atom that is next to two Co atoms in NiCo hydroxide. The bond with the highest bond strength was chosen for comparison out of the two available Co-oxygen bonds and the position of the bond was also found to be on same position as Ni and Co hydroxide. As a consequence, after oxygen vacancy formation, the -ICOHP value of Co-O bond on NiCo hydroxide was largely decreased while that of Ni hydroxide, Co hydroxide, and Ni-O bond on NiCo hydroxide increased when oxygen vacancy was formed. This implies that the bond strength of Co-O on NiCo hydroxide has become weaker after forming oxygen vacancy, owing to the accumulation of electrons on Co site[40].

Supplementary note 8: transition states on 2nd C-C cleavage step

For 2nd C-C cleavage step, which is a rate determining step, the identification of transition state and the calculation of activation energy were performed by climbing image nudged elastic band method (CI-NEB)[41]. According to the obtained activation energy on **Supplementary Fig. 39**, NiCo (oxy)hydroxide shows the lowest energy barrier with activation energy of 1.70 eV while Co (oxy)hydroxide shows the highest energy barrier with 2.64 eV on 2nd C-C cleavage step. This result is consistent with the obtained reaction free energies. The activation energy also shows strong correlation with the d-band filling of metal site on hydroxide (**Supplementary Fig. 40**), similar to that of the free energy barrier shown in Fig. 4e of the manuscript.

Supplementary note 9: Charge transfer and relative adsorption energy.

The relative adsorption energies and amount of charge transferred to the cleaved molecules were calculated for the second cleavage steps to validate the devotedness of electrons during C-C bond cleavage towards the ability of stabilizing the cleaved molecules. The relative adsorption energy was calculated by using equations of (30) and (31).

$$E_{relative\_ads}(X) = E_{mol/surface}(X) - E_{Surface}(X) - E(NiCo) \quad (30)$$

$$E(NiCo) = E_{mol/surface}(NiCo) - E_{Surface}(NiCo) \quad (31)$$

where $E_{relative\_ads}(X)$ is the relative adsorption energy of catalyst X (X = Ni, NiCo, Co) at the second C-C bond cleavage step, $E_{mol/surface}(X)$ is the total energy of

surface and cleaved molecules on its surface, and $E_{Surface}(X)$is the total energy of surface without cleaved molecules. In general, the calculated relative adsorption energies represent the ability to stabilize the cleaved molecules in comparison to the case of NiCo hydroxide.

As shown in **Supplementary Fig. 41**, the obtained relative adsorption energies are with a trend of NiCo < Ni < Co hydroxide. Furthermore, results of bader charge analysis of cleaved molecules were also found to be well correlated to the relative adsorption energies. The relative adsorption energies were found to be stronger when more charges were transferred to the cleaved molecules owing to the redistribution of electrons on the metal sites. (**Supplementary Fig. 42**).

Supplementary note 10: active site for 2nd C-C cleavage on NiCo hydroxide

We also considered alternative reaction pathways other than the one chosen in the manuscript for the second C-C cleavage step of NiCo (oxy)hydroxide. We compared the reaction energy of the second C-C cleavage step occurring on nickel sites to that of the preferred pathway involving Co sites. In addition to the second C-C cleavage step, we also considered the impact of reaction site for the hydrogenation step prior to the second C-C cleavage. As shown in **Supplementary Fig. 44a**, two different hydrogenation schemes were investigated. If the hydrogenation takes place from oxygen on Ni site, the glycolate would attack the Ni site oxygen to perform the second C-C cleavage while the opposite case allows second C-C cleavage of glycolate on Co sites. Our calculation results indicate that a step before the second C-C cleavage with

Co site hydrogenation has 0.01 eV lower total energy compared to that of Ni site hydrogenation. Along with the aforementioned prediction, the reaction energy of second C-C cleavage on Ni sites reveals much higher energy (2.29 eV) compared to the case with second C-C cleavage on Co sites (0.51 eV). Therefore, we concluded that second C-C cleavage step on Co sites is energetically favorable than the Ni sites. The **Supplementary Fig. 44b** depicts the images of second C-C cleavage step that involves Ni sites and its corresponding reaction energy.

Supplementary note 11: Chemicals and materials.

Glycerol ($C_2H_8O_3$, 99%) was purchased from Guangzhou Chemical Reagent Factory. Glycolic acid ($C_2H_4O_3$, 98%), 2,3-Dihydroxypropanoic acid (C_3H6O4, 95%, 20% in water), formic acid (CH_2O_2, 99%), nickel acetate tetrahydrate ($NiC_4H_6O_4 \cdot 4H_2O$, 99.9%) were purchased from Shanghai Macklin Biochemical Co., Ltd. Cobalt acetate tetrahydrate ($CoC_4H_6O_4 \cdot 4H_2O$, 99.5%) was purchased from Kermel chemical reagent Co., Ltd. Potassium hydroxide (KOH, 85%) was purchased from Damao chemical reagent factory. All reagents were used as-received without further purification. Carbon cloths (WOS1009) was purchased from Ce-Tech Co., Ltd. Proton exchange membrane (Nafion 117) was purchased from Dupont China Holding Co., Ltd.

Supplementary note 12: DFT calculation.

Spin-polarized DFT simulations were performed using Vienna ab initio Simulation package (VASP, version 5.4.4).[42, 43] The projector augmented-wave (PAW) potential[44]

and the Perdew-Burke-Ernzerhof (PBE) functional based generalized gradient approximation (GGA)[45] were employed. The DFT-D3 method with Becke-Jonson damping was adopted to correct the weak van der waals' interactions for layer structures.[46, 47] We considered the subsequent explicit electrons for each element: Ni (3p, 3d, 4s); Co (3p, 3d, 4s); O (2s, 2p); C (2s, 2p); and H (1s). To treat the strong onsite coulomb interactions, our calculations were computed by the DFT+U method of Dudarev's scheme[48] and values of 5.5 eV and 3.3 eV were chosen for U_{eff} (U-J correction) of Ni and Co, respectively. These values were taken from Li et al[49] and Wang et al[50]. For bulk models of β-$M(OH)_2$, and β-M(OOH) (M= Ni, NiCo, or Co), an energy cut-off of 750 eV and 6 x 6 x 4 of Γ-point-centered Monkhorst-Pack k-point mesh were set to optimize the bulk structures. The further details of bulk modellings including the location of hydrogen atoms on M(OOH) models are shown in **Supplementary Note 13**. The surface models were established with a single layer of hydrogen-terminated (001) plane of 5 x 5 supercells, and energy cut-off of 500 eV[51, 52] with a 3 x 3 x 1 Γ-point-centered Monkhorst-Pack k-point mesh was employed. For each surface model, 15 Å of vacuum layer was introduced to prevent the vertical interactions of periodic images. All structures including the bulk structures were allowed to be relaxed until the force and energy convergence threshold of 0.02 eV/ Å and 10^{-5} eV, respectively. The atomic ratios of $NiCo(OH)_2$ and NiCo(OOH) were set to be 1 and each metal atom was arranged to have different element on neighboring metal atoms. The calculated density of state (DOS) was employed to perform the calculation of crystal orbital hamiltonian population (COHP) by LOBSTER program.[53]

The surface energy (γ) was calculated by equation of (32).

$$\gamma = \frac{(E_{surface\_total} - 2E_{bulk\_total})}{2A} \quad (32)$$

where $E_{surface\_total}$ is the total energy of surface models with 16 metal, 32 oxygen and 16 hydrogen atoms. E_{bulk_total} is the total energy of bulk models with 8 metal, 16 oxygen, and 8 hydrogen atoms while A indicates the surface area of slab model.

The adsorption energy (E_{ads}) was obtained by following equation of (33).

$$E_{ads} = E_{mol/surface} - E_{surface} - E_{mol} \quad (33)$$

where $E_{mol/surface}$ is the total energy of surface models with adsorbed molecules on its surface, $E_{surface}$ is the total energy of bare surface models, and E_{mol} is gas-phase energy of adsorbates.

The oxygen vacancy formation energy E_{f_vac} and deprotonation energy of surface models were calculated by equation (34) and (35), respectively.

$$E_{f\_vac} = E_{surface-nO_v} + n\frac{1}{2}E_{O_2} - E_{surface} \quad (34)$$

$$E_{deprotonation} = E_{surface-1H} + \frac{1}{2}E_{H_2} - E_{surface} \quad (35)$$

where $E_{Surface-nO_v}$ indicates that the total energy of surface model with n number of oxygen vacancies and $E_{Surface-1H}$ indicates the total energy of surface model with 1 defect hydrogen.

Supplementary note 13: Bulk and surface structures optimization of oxyhydroxide

The bulk models of oxyhydroxide were prepared by removing some hydrogen atoms from bulk structures of hydroxide models. Three possible bulk structures of oxyhydroxide models were considered to obtain slab models for reaction mechanism

study (**Supplementary Fig. 47**). Each case of oxyhydroxide bulk structure was modelled to have different location of hydrogen atoms and re-optimized to give newly obtained total energy and lattice parameters. Since the number of atoms and stoichiometric were same for all cases of oxyhydroxide structures, the total energies are directly comparable to define which structure is the most stable for oxyhydroxide. Among the three bulk structures of oxyhydroxide, structures with the location of hydrogen atoms with chainsaw-like placement were found to have the lowest total energy (**Supplementary Fig. 47b**) and all of Ni, NiCo, and Co oxyhydroxide showed the consistent result. The computed values of optimized lattice parameters of hydroxide and (oxy)hydroxide models with chainsaw-like arrangement of hydrogen were as follow: $Co(OH)_2$ (a = b = 6.409 Å ; c = 9.101 Å), $NiCo(OH)_2$ (a = b = 6.399 Å ; c = 9.032 Å), $Ni(OH)_2$ (a = b = 6.295 Å ; c = 9.052 Å), CoOOH (a = b = 6.091 Å ; c = 9.124 Å), NiCoOOH (a = b = 5.948 Å ; c = 9.022 Å), and NiOOH (a = b = 5.868 Å ; c = 9.063 Å). As the hydroxide model is transformed to the oxyhydroxide model, the lattice parameters of a and b dimensions is shrunk while that of on c dimension does not have significant changes. Moreover, the surface energy calculation was performed by using equation (33). The computed surface energies of hydrogen atoms with chainsaw-like placement of Co, NiCo, and Ni (oxy)hydroxides were 0.66, 0.53, and 0.58 J/m^2, respectively. We also conducted bulk calculation with different initial spin configurations for each bulk structure. As a result of calculation (**Supplementary Fig. 48 and Supplementary Table 5**), we concluded that structures with chain-saw like placement of hydrogen were still the most stable compared to other structures. In this

study, this particular bulk structure of oxyhydroxide was used to develop the slab models for reaction mechanism study.

The band gap calculations were conducted on both bulk and surface models. As shown in **Supplementary Table 6**, the band gap energies computed for Co, NiCo, and Ni hydroxides are 1.67, 2.66, and 3.33 eV, respectively, indicating semiconductor characteristics. However, the surface models exhibit almost metallic properties with band gap of 0.01 to 0.61 eV. Such results about the small band gap of hydroxide surface are consistent with the previous reports[54, 55].

References

1. Li, Y., Wei, X., Chen, L., Shi, J., He, M. Nickel-molybdenum nitride nanoplate electrocatalysts for concurrent electrolytic hydrogen and formate productions. *Nat. Commun.* **10**, 5335 (2019).
2. Hao, J., *et al.* In situ facile fabrication of $Ni(OH)_2$ nanosheet arrays for electrocatalytic co-production of formate and hydrogen from methanol in alkaline solution. *Appl. Catal. B-Environ.* **281**, 119510 (2021).
3. Zhang, N., *et al.* Electrochemical oxidation of 5-hydroxymethylfurfural on nickel nitride/carbon nanosheets: Reaction pathway determined by in situ sum frequency generation vibrational spectroscopy. *Angew. Chem. Int. Ed. Engl.* **58**, 15895-15903 (2019).
4. Gao, L., *et al.* Oxygen Vacancy-induced Electron Density Tuning of Fe_3O_4 for Enhanced Oxygen Evolution Catalysis. *Energy & Environmental Materials* **4**, 392-398 (2020).
5. You, B., Liu, X., Liu, X., Sun, Y. Efficient H_2 evolution coupled with oxidative refining of alcohols via a hierarchically porous nickel bifunctional electrocatalyst. *ACS Catal.* **7**, 4564-4570 (2017).
6. Gao, L., *et al.* NiSe@NiO_x core-shell nanowires as a non-precious electrocatalyst for upgrading 5-hydroxymethylfurfural into 2,5-furandicarboxylic acid. *Appl. Catal. B-Environ.* **261**, 118235 (2020).
7. Jiang, N., You, B., Boonstra, R., Terrero Rodriguez, I. M., Sun, Y. Integrating electrocatalytic 5-hydroxymethylfurfural oxidation and hydrogen production via Co-P-derived electrocatalysts. *ACS Energy Lett.* **1**, 386-390 (2016).
8. You, B., Liu, X., Jiang, N., Sun, Y. A general strategy for decoupled hydrogen production from water splitting by integrating oxidative biomass valorization. *J. Am. Chem. Soc.* **138**, 13639-13646 (2016).
9. You, B., Jiang, N., Liu, X., Sun, Y. Simultaneous H_2 generation and biomass upgrading in water by an efficient noble-metal-free bifunctional electrocatalyst. *Angew. Chem. Int. Ed. Engl.* **55**,

9913-9917 (2016).
10. Wang, J., *et al.* Ni/NiO heterostructures encapsulated in oxygen-doped graphene as multifunctional electrocatalysts for the HER, UOR and HMF oxidation reaction. *Catal. Sci. Technol.* **11**, 2480-2490 (2021).
11. Zhang, Y., *et al.* Core-corona Co/CoP clusters strung on carbon nanotubes as a Schottky catalyst for glucose oxidation assisted H_2 production. *J. Mater. Chem. A* **9**, 10893-10908 (2021).
12. Sheng Chen, J. D., Anthony Vasileff, Shi Zhang Qiao. Size fractionation of two-dimensional sub-nanometer thin manganese dioxide crystals towards superior urea electrocatalytic conversion. *Angew. Chem.* **128**, 3868-3872 (2016).
13. Lyu, C., Zheng, J., Zhang, R., Zou, R., Liu, B., Zhou, W. Homologous Co_3O_4‖CoP nanowires grown on carbon cloth as a high-performance electrode pair for triclosan degradation and hydrogen evolution. *Mater. Chem. Front.* **2**, 323-330 (2018).
14. Gao, D., *et al.* Activation of the $MoSe_2$ basal plane and Se-edge by B doping for enhanced hydrogen evolution. *Journal of Materials Chemistry A* **6**, 510-515 (2018).
15. Liu, Q., *et al.* A porous Ni_3N nanosheet array as a high-performance non-noble-metal catalyst for urea-assisted electrochemical hydrogen production. *Inorg. Chem. Front.* **4**, 1120-1124 (2017).
16. Zheng, J., *et al.* Hierarchical porous NC@CuCo nitride nanosheet networks: Highly efficient bifunctional electrocatalyst for overall water splitting and selective electrooxidation of benzyl alcohol. *Adv. Funct. Mater.* **27**, 1704169 (2017).
17. Sun, C. B., Guo, M. W., Siwal, S. S., Zhang, Q. B. Efficient hydrogen production via urea electrolysis with cobalt doped nickel hydroxide-riched hybrid films: Cobalt doping effect and mechanism aspect. *J. Catal.* **381**, 454-461 (2020).
18. Wang, C., Lu, H., Mao, Z., Yan, C., Shen, G., Wang, X. Bimetal schottky heterojunction boosting energy-saving hydrogen production from alkaline water via urea electrocatalysis. *Adv. Funct. Mater.* **30**, 2000556 (2020).
19. Xiang, K., *et al.* Boosting H_2 generation coupled with selective oxidation of methanol into value-added chemical over cobalt hydroxide@hydroxysulfide nanosheets electrocatalysts. *Adv. Funct. Mater.* **30**, 1909610 (2020).
20. Jong Ju Lee, M. Y. O., Kee Suk Nahm. Effect of Ball Milling on Electrocatalytic Activity of Perovskite $La_{0.6}Sr_{0.4}CoO_{3-\delta}$ Applied for Lithium Air Battery. *Journal of The Electrochemical Society* **163**, A244-A250 (2016).
21. Song, Y., *et al.* Ultrathin layered double hydroxides nanosheets array towards efficient electrooxidation of 5-hydroxymethylfurfural coupled with hydrogen generation. *Appl. Catal. B-Environ.* **299**, 120669 (2021).
22. Jiang, H., Sun, M., Wu, S., Huang, B., Lee, C. S., Zhang, W. Oxygen-incorporated NiMoP nanotube arrays as efficient bifunctional electrocatalysts for urea-assisted energy-saving hydrogen production in alkaline electrolyte. *Adv. Funct. Mater.* **31**, 2104951 (2021).
23. Geng, S.-K., *et al.* Nickel ferrocyanide as a high-performance urea oxidation electrocatalyst. *Nat. Energy* **6**, 904-912 (2021).
24. Lu, X. F., Zhang, S. L., Sim, W. L., Gao, S., Lou, X. W. Phosphorized $CoNi_2S_4$ yolk-shell spheres for highly efficient hydrogen production via water and urea electrolysis. *Angew. Chem. Int. Edit.* **60**, 22885-22891 (2021).
25. Wu, J., *et al.* Steering the glycerol electro-reforming selectivity via cation-intermediate

interactions. *Angew. Chem. Int. Ed. Engl.*, (2021).

26. Ke, Z., *et al.* Solar-assisted co-electrolysis of glycerol and water for concurrent production of formic acid and hydrogen. *J. Mater. Chem. A* **9**, 19975-19983 (2021).

27. Li, R.-Q., *et al.* 3D self-supported porous vanadium-doped nickel nitride nanosheet arrays as efficient bifunctional electrocatalysts for urea electrolysis. *J. Mater. Chem. A* **9**, 4159-4166 (2021).

28. Zhang, B., *et al.* Interface engineering: The $Ni(OH)_2/MoS_2$ heterostructure for highly efficient alkaline hydrogen evolution. *Nano Energy* **37**, 74-80 (2017).

29. Liu, S.-Q., *et al.* Amorphous $Ni(OH)_2$ encounter with crystalline CuS in hollow spheres: A mesoporous nano-shelled heterostructure for hydrogen evolution electrocatalysis. *Nano Energy* **44**, 7-14 (2018).

30. Liang, C., *et al.* Exceptional performance of hierarchical Ni-Fe oxyhydroxide@NiFe alloy nanowire array electrocatalysts for large current density water splitting. *Energy Environ. Sci.* **13**, 86-95 (2020).

31. Liu, X., *et al.* Uncovering the effect of lattice strain and oxygen deficiency on electrocatalytic activity of perovskite cobaltite thin films. *Adv. Sci.* **6**, 1801898 (2019).

32. Chen, G., *et al.* Accelerated hydrogen evolution kinetics on NiFe-layered double hydroxide electrocatalysts by tailoring water dissociation active sites. *Adv. Mater.* **30**, (2018).

33. Cai, Z., *et al.* Simple and cost effective fabrication of 3D porous core-shell Ni nanochains@NiFe layered double hydroxide nanosheet bifunctional electrocatalysts for overall water splitting. *J. Mater. Chem. A* **7**, 21722-21729 (2019).

34. Yang, J., Yu, C., Fan, X., Qiu, J. 3D architecture materials made of NiCoAl-LDH nanoplates coupled with NiCo-carbonate hydroxide nanowires grown on flexible graphite paper for asymmetric supercapacitors. *Adv. Energy Mater.* **4**, 1400761 (2014).

35. Youngkook Kwon, M. T. M. K. Combining Voltammetry with HPLC: Application to Electro-Oxidation of Glycerol. *Analytical Chemistry* **82**, 5420–5424 (2010).

36. Santiago, P. V. B., Lima, C. C., Bott-Neto, J. L., Fernández, P. S., Angelucci, C. A., Souza-Garcia, J. Perovskite oxides as electrocatalyst for glycerol oxidation. *Journal of Electroanalytical Chemistry* **896**, 115198 (2021).

37. Lima, C. C., *et al.* Highly active Ag/C nanoparticles containing ultra-low quantities of sub-surface Pt for the electrooxidation of glycerol in alkaline media. *Applied Catalysis B: Environmental* **279**, 119369 (2020).

38. Yang, J., *et al.* Surface-confined fabrication of ultrathin nickel cobalt-layered double hydroxide nanosheets for high-performance supercapacitors. *Adv. Funct. Mater.* **28**, 1803272 (2018).

39. Wang, Z., *et al.* Contemporaneous inverse manipulation of the valence configuration to preferred Co^{2+} and Ni^{3+} for enhanced overall water electrocatalysis. *Appl. Catal. B* **284**, 119725 (2021).

40. Fung, V., Wu, Z., Jiang, D.-e. New bonding model of radical adsorbate on lattice oxygen of perovskites. *The journal of physical chemistry letters* **9**, 6321-6325 (2018).

41. Henkelman G, U. B. P., Jónsson H. A climbing image nudged elastic band method for finding saddle points and minimum energy paths. *J Chem Phys* **113**, 9901-9904 (2000).

42. Sholl, D., Steckel, J. A. *Density functional theory: a practical introduction*. John Wiley & Sons (2011).

43. Kresse, G., Furthmüller, J. Efficient iterative schemes for ab initio total-energy calculations

using a plane-wave basis set. *Physical review B* **54**, 11169 (1996).

44. Blöchl, P. E. Projector augmented-wave method. *Physical review B* **50**, 17953 (1994).

45. Perdew, J. P., Burke, K., Ernzerhof, M. Generalized gradient approximation made simple. *Physical review letters* **77**, 3865 (1996).

46. Grimme, S., Ehrlich, S., Goerigk, L. Effect of the damping function in dispersion corrected density functional theory. *Journal of computational chemistry* **32**, 1456-1465 (2011).

47. Grimme, S., Antony, J., Ehrlich, S., Krieg, H. A consistent and accurate ab initio parametrization of density functional dispersion correction (DFT-D) for the 94 elements H-Pu. *The Journal of chemical physics* **132**, 154104 (2010).

48. Dudarev, S., Botton, G., Savrasov, S., Humphreys, C., Sutton, A. Electron-energy-loss spectra and the structural stability of nickel oxide: An LSDA+ U study. *Physical Review B* **57**, 1505 (1998).

49. Li, Y.-F., Selloni, A. Mechanism and activity of water oxidation on selected surfaces of pure and Fe-doped NiO x. *Acs Catalysis* **4**, 1148-1153 (2014).

50. Wang, L., Maxisch, T., Ceder, G. Oxidation energies of transition metal oxides within the GGA+ U framework. *Physical Review B* **73**, 195107 (2006).

51. Mefford, J. T., Zhao, Z., Bajdich, M., Chueh, W. C. Interpreting Tafel behavior of consecutive electrochemical reactions through combined thermodynamic and steady state microkinetic approaches. *Energy & Environmental Science* **13**, 622-634 (2020).

52. Zhang, N., *et al.* Lattice oxygen activation enabled by high-valence metal sites for enhanced water oxidation. *Nat. Commun.* **11**, 4066 (2020).

53. Maintz, S., Deringer, V. L., Tchougréeff, A. L., Dronskowski, R. LOBSTER: A tool to extract chemical bonding from plane-wave based DFT.). Wiley Online Library (2016).

54. Zhang, S., *et al.* The latest development of CoOOH two-dimensional materials used as OER catalysts. *Chem Commun (Camb)* **56**, 15387-15405 (2020).

55. Zhu, X., *et al.* Metallic Nickel Hydroxide Nanosheets Give Superior Electrocatalytic Oxidation of Urea for Fuel Cells. *Angew Chem Int Ed Engl* **55**, 12465-12469 (2016).
