# Supplementary material for: Promoting biomass electrooxidation via modulating proton and oxygen anion deintercalation in hydroxide
Source: Nat Commun. 2022 Jun 30;13:3777. doi: 10.1038/s41467-022-31484-0 (PMC9246976; doi:10.1038/s41467-022-31484-0)
Supplement: Supplementary file 3 — Description of Additional Supplementary Files [file 41467_2022_31484_MOESM3_ESM.pdf]

Description of Additional Supplementary Files

Supplementary Dataset Legends:

Supplementary Dataset 1. The coordinates of the cobalt surface. The energy of the cobalt surface was calculated to be -353.88 eV.

Supplementary Dataset 2. The coordinates of the nickel/cobalt surface. The energy of the nickel/cobalt surface was calculated to be -334.25 eV.

Supplementary Dataset 3. The coordinates of the nickel surface. The energy of the nickel surface was calculated to be -310.36 eV.

Supplementary Dataset 4. The coordinates of the glycerol adsorption step on cobalt surface. The energy of the glycerol adsorption step on cobalt surface was calculated to be -431.31 eV.

Supplementary Dataset 5. The coordinates of the glycerol adsorption step on nickel/cobalt surface. The energy of the glycerol adsorption step on nickel/cobalt surface was calculated to be -411.50 eV

Supplementary Dataset 6. The coordinates of the glycerol adsorption step on nickel surface. The energy of the glycerol adsorption step on nickel surface was calculated to be -387.95 eV.

Supplementary Dataset 7. The coordinates of the glyceraldehyde formation step on cobalt surface. The energy of the glyceraldehyde formation step on cobalt surface was calculated to be -433.61 eV.

Supplementary Dataset 8. The coordinates of the glyceraldehyde formation step on nickel/cobalt surface. The energy of the glyceraldehyde formation step on nickel/cobalt surface was calculated to be -413.30 eV.

Supplementary Dataset 9. The coordinates of the glyceraldehyde formation step on nickel surface. The energy of the glyceraldehyde formation step on nickel surface was calculated to be -389.31 eV

Supplementary Dataset 10. The coordinates of the glycerate : lattice oxygen attack step on cobalt surface. The energy of the glycerate : lattice oxygen attack step on cobalt surface was calculated to be -436.62 eV.

Supplementary Dataset 11. The coordinates of the glycerate : lattice oxygen attack step on nickel/cobalt surface. The energy of the glycerate : lattice oxygen attack step on nickel/cobalt surface was calculated to be -417.00 eV

Supplementary Dataset 12. The coordinates of the glycerate : lattice oxygen attack step on nickel surface. The energy of the glycerate : lattice oxygen attack step on nickel surface was calculated to be -392.15 eV.

Supplementary Dataset 13. The coordinates of the glyceric acid formation step on cobalt surface. The energy of the glyceric acid formation step on cobalt surface was calculated to be -436.60 eV.

Supplementary Dataset 14. The coordinates of the glyceric acid formation step on nickel/cobalt surface. The energy of the glyceric acid formation step on nickel/cobalt surface was calculated to be -416.00 eV

Supplementary Dataset 15. The coordinates of the glyceric acid formation step on nickel surface. The energy of the glyceric acid formation step on nickel surface was calculated to be -391.34 eV.

Supplementary Dataset 16. The coordinates of the glyceric acid desorption step on cobalt surface. The energy of the glyceric acid desorption step on cobalt surface was calculated to be -435.39 eV [-359.19 eV – 76.20 eV (glyceric acid)].

Supplementary Dataset 17. The coordinates of the glyceric acid desorption step on nickel/cobalt surface. The energy of the glyceric acid desorption step on nickel/cobalt surface was calculated to be -414.15 eV [-337.95 eV – 76.20 eV (glyceric acid)].

Supplementary Dataset 18. The coordinates of the glyceric acid desorption step on nickel surface. The energy of the glyceric acid desorption step on nickel surface was calculated to be -390.14 eV [-313.94 eV – 76.20 eV (glyceric acid)].

Supplementary Dataset 19. The coordinates of the glycerate : configuration changed step on cobalt surface. The energy of the glycerate : configuration changed step on cobalt surface was calculated to be -437.10 eV.

Supplementary Dataset 20. The coordinates of the glycerate : configuration changed step on nickel/cobalt surface. The energy of the glycerate : configuration changed step on nickel/cobalt surface was calculated to be -417.44 eV.

Supplementary Dataset 21. The coordinates of the glycerate : configuration changed step on nickel surface. The energy of the glycerate : configuration changed step on nickel surface was calculated to be -392.33 eV

Supplementary Dataset 22. The coordinates of the glycerate : 2 nd lattice oxygen attack step on cobalt surface. The energy of the glycerate : 2nd lattice oxygen attack step on cobalt surface was calculated to be -440.60 eV

Supplementary Dataset 23. The coordinates of the glycerate : 2 nd lattice oxygen attack step on nickel/cobalt surface. The energy of the glycerate : 2nd lattice oxygen attack step on nickel/cobalt surface was calculated to be -419.48 eV

Supplementary Dataset 24. The coordinates of the glycerate : 2 nd lattice oxygen attack step on nickel surface. The energy of the glycerate : 2nd lattice oxygen attack step on nickel surface was calculated to be -394.53 eV.

Supplementary Dataset 25. The coordinates of the 1 st C-C cleavage step on cobalt surface. The energy of the 1st C-C cleavage step on cobalt surface was calculated to be -439.10 eV.

Supplementary Dataset 26. The coordinates of the 1 st C-C cleavage step on nickel/cobalt surface. The energy of the 1st C-C cleavage step on nickel/cobalt surface was calculated to be -417.96 eV.

Supplementary Dataset 27. The coordinates of the 1 st C-C cleavage step on nickel surface. The energy of the 1st C-C cleavage step on nickel surface was calculated to be -393.01 eV.

Supplementary Dataset 28. The coordinates of the 7A. hydrogenation step on cobalt surface. The energy of the 7A. hydrogenation step on cobalt surface was calculated to be -437.85 eV

Supplementary Dataset 29. The coordinates of the 7A. hydrogenation step on nickel/cobalt surface. The energy of the 7A. hydrogenation step on nickel/cobalt surface was calculated to be -417.67 eV.
Supplementary Dataset 30. The coordinates of the 7A. hydrogenation step on nickel surface. The energy of the 7A. hydrogenation step on nickel surface was calculated to be -392.80 eV.

Supplementary Dataset 31. The coordinates of the 1 st formic acid desorption step on cobalt surface. The energy of the 1st formic acid desorption step on cobalt surface was calculated to be -437.24 eV [-407.50 eV - 29.74 eV (formic acid)].

Supplementary Dataset 32. The coordinates of the 1 st formic acid desorption step on nickel/cobalt surface. The energy of the 1st formic acid desorption step on nickel/cobalt surface was calculated to be -416.58 eV [-386.84 eV - 29.74 eV (formic acid)].

Supplementary Dataset 33. The coordinates of the 1 st formic acid desorption step on nickel surface. The energy of the 1st formic acid desorption step on nickel surface was calculated to be -391.56 eV [-361.82 eV - 29.74 eV (formic acid)].

Supplementary Dataset 34. The coordinates of the glycolic acid formation step on cobalt surface. The energy of the glycolic acid formation step on cobalt surface was calculated to be -437.30 eV [-407.56 eV - 29.74 eV (formic acid)].

Supplementary Dataset 35. The coordinates of the glycolic acid formation step on nickel/cobalt surface. The energy of the glycolic acid formation step on nickel/cobalt surface was calculated to be -416.31 eV [-386.57 eV - 29.74 eV (formic acid)].

Supplementary Dataset 36. The coordinates of the glycolic acid formation step on nickel surface. The energy of the glycolic acid formation step on nickel surface was calculated to be -391.28 eV [-361.54 eV - 29.74 eV (formic acid)].

Supplementary Dataset 37. The coordinates of the glycolic acid desorption step on cobalt surface. The energy of the glycolic acid desorption step on cobalt surface was calculated to be -436.95 eV [-354.19 eV - 82.76 eV (formic acid + glycolic acid)].

Supplementary Dataset 38. The coordinates of the glycolic acid desorption step on nickel/cobalt surface. The energy of the glycolic acid desorption step on nickel/cobalt surface was calculated to be -415.60 eV [-332.84 eV - 82.76 eV (formic acid + glycolic acid)]

Supplementary Dataset 39. The coordinates of the glycolic acid desorption step on nickel surface. The energy of the glycolic acid desorption step on nickel surface was calculated to be -390.64 eV [-307.88 eV - 82.76 eV (formic acid + glycolic acid)].

Supplementary Dataset 40. The coordinates of the glycolate : 3rd lattice oxygen attack step on cobalt surface. The energy of the glycolate : 3rd lattice oxygen attack step on cobalt surface was calculated to be -439.83 eV [-410.09 eV -29.74 eV (formic acid)].

Supplementary Dataset 41. The coordinates of the glycolate : 3rd lattice oxygen attack step on nickel/cobalt surface. The energy of the glycolate : 3rd lattice oxygen attack step on nickel/cobalt surface was calculated to be -418.68 eV [-388.94 eV -29.74 eV (formic acid)].

Supplementary Dataset 42. The coordinates of the glycolate : 3rd lattice oxygen attack step on nickel surface. The energy of the glycolate : 3rd lattice oxygen attack step on nickel surface was calculated to be -393.84 eV [-364.10 eV -29.74 eV (formic acid)].

Supplementary Dataset 43. The coordinates of the 2 nd C-C cleavage step on cobalt surface. The energy of the 2 nd C-C cleavage step on cobalt surface was calculated to be -437.61 eV [-407.87 eV -29.74 eV (formic acid)].

Supplementary Dataset 44. The coordinates of the 2 nd C-C cleavage step on nickel/cobalt surface. The energy of the 2 nd C-C cleavage step on nickel/cobalt surface was calculated to be -418.17 eV [-388.43 eV -29.74 eV (formic acid)].

Supplementary Dataset 45. The coordinates of the 2 nd C-C cleavage step on nickel surface. The energy of the 2nd C-C cleavage step on nickel surface was calculated to be -392.00 eV [-362.26 eV -29.74 eV (formic acid)].

Supplementary Dataset 46. The coordinates of the 9B. hydrogenation step on cobalt surface. The energy of the 9B. hydrogenation step on cobalt surface was calculated to be -437.84 eV [-408.10 eV -29.74 eV (formic acid)].

Supplementary Dataset 47. The coordinates of the 9B. hydrogenation step on nickel/cobalt surface. The energy of the 9B. hydrogenation step on nickel/cobalt surface was calculated to be -418.21 eV [-388.47 eV -29.74 eV (formic acid)].

Supplementary Dataset 48. The coordinates of the 9B. hydrogenation step on nickel surface. The energy of the 9B. hydrogenation step on nickel surface was calculated to be -392.35 eV [-362.61 eV -29.74 eV (formic acid)]

Supplementary Dataset 49. The coordinates of the 2 nd formic acid desorption step on cobalt surface. The energy of the 2nd formic acid desorption step on cobalt surface was calculated to be -436.87 eV [-347.65 eV –3*(29.74 eV) (formic acid)]

Supplementary Dataset 50. The coordinates of the 2 nd formic acid desorption step on nickel/cobalt surface. The energy of the 2nd formic acid desorption step on nickel/cobalt surface was calculated to be -417.05 eV [-327.83 eV –3*(29.74 eV) (formic acid)].

Supplementary Dataset 51. The coordinates of the 2nd formic acid desorption step on nickel surface. The energy of the 2nd formic acid desorption step on nickel surface was calculated to be -390.89 eV [-301.67 eV –3*(29.74 eV) (formic acid)].
