# Supplementary material for: Promoting biomass electrooxidation via modulating proton and oxygen anion deintercalation in hydroxide
Source: Nat Commun. 2022 Jun 30;13:3777. doi: 10.1038/s41467-022-31484-0 (PMC9246976; doi:10.1038/s41467-022-31484-0)
Supplement: Supplementary file 4 — Supplementary dataset [file 41467_2022_31484_MOESM4_ESM.pdf]

Supplementary Dataset

Supplementary Dataset 1. The coordinates of the cobalt surface. The energy of the cobalt surface was calculated to be -353.88 eV.

Co O H

1.00000000000000

12.1820001601999994	0.0000000000000000	0.0000000000000000
-6.0910000800999997	10.5499216077000000	0.0000000000000000
0.0000000000000000	0.0000000000000000	21.1058006286999991

Co O H

16 32 16

Direct

0.9493683570009536	0.1511177051666533	0.0943610114059847
0.9535302122126942	0.6518031022016822	0.0954824528559536
0.4510768921353153	0.1491875948107638	0.0953776882960514
0.4492252849811981	0.6471245587525292	0.0928016982831698
0.9497472486559982	0.8988597597817469	0.0916497453480071
0.9491028639260598	0.4034132842791834	0.0958060681855692
0.4537106833891746	0.8973748387305817	0.0900068189828307
0.4471884707055879	0.3989668676484159	0.0954496409438597
0.7048083328775033	0.1549126718088223	0.0929344937551150
0.6973699965633974	0.6537592719514077	0.0897100081402589

0.1993119103991172	0.1481881068993335	0.0948236705063304
0.1997636132826488	0.6520205382954712	0.0947394431789913
0.6982799108598863	0.9006333111132677	0.0935209306678191
0.7048451067288735	0.4083982954864571	0.0996031499455396
0.2015447999598719	0.9015864399036864	0.0916902941627687
0.1995623417507704	0.4000598313331812	0.0945566290491553
0.0286074875889127	0.3284802058791101	0.1448439579943891
0.0367690604786072	0.8187037521573123	0.1459377477017171
0.5355166893352091	0.3178433171880950	0.1479352328977039
0.5384641004573104	0.8273483130972270	0.1469379434726557
0.0358788830471068	0.0599477843438518	0.1343201095030885
0.0294654964368401	0.5635639223937533	0.1381474940930756
0.5362952410365427	0.0654516162092521	0.1342474997179520
0.5209047918197566	0.5576294631048225	0.1350006147300359
0.8020117285955782	0.3363382273031509	0.1483819944896550
0.7877620122918665	0.8206219671120215	0.1446346919801177
0.2848406016048484	0.3240727678742593	0.1489763392531573
0.2894304138089900	0.8225037076674918	0.1451811964964996
0.7824894904618850	0.0821901217315665	0.1361571876537116
0.7763677417737543	0.5636611222228298	0.1383503101875106
0.2739107978120318	0.0585472215160088	0.1356794887993243
0.2829605472082761	0.5634657413627624	0.1328472636861416

0.1191694050289648	0.2373232360947684	0.0548120196965183
0.1253012631973230	0.7380928620880804	0.0503113298135978
0.6338009875763465	0.2425987801063553	0.0552816141597810
0.6086816449861491	0.7258349461354860	0.0509664241056723
0.1115436256496158	0.9716098105968617	0.0376865557332248
0.1006059529845689	0.4741166517018141	0.0450127538022249
0.6171236211410438	0.9851947724215052	0.0380641600588724
0.6143039724912623	0.4764164148337343	0.0424342865782997
0.8680512507126000	0.2476768986620469	0.0517558453059151
0.8725250869913461	0.7361115492727025	0.0502644577738384
0.3659731742074518	0.2334378561730605	0.0547491743223309
0.3772582528196670	0.7383419334800311	0.0511024251823189
0.8663804868444571	0.9856721729418704	0.0419389358178355
0.8706833977397622	0.4776854954211913	0.0459354243379138
0.3691563110291928	0.9840182165600737	0.0400449646028613
0.3568159076544214	0.4667189718035952	0.0390884453598612
0.1042755194483588	0.9562177834103220	-0.0076056327906176
0.0892486311045573	0.4631656043940157	-0.0005810227669748
0.6134386863189094	0.9826114341179518	-0.0078200632459881
0.6133899321977171	0.4688855941356695	-0.0033551753799536
0.8694686793692207	0.9896383971276432	-0.0039766231459333
0.8641592451547750	0.4585428230961741	0.0009583066973900

0.3693248308607518 0.9881316104807937 -0.0058534835517131

0.3481987164234421 0.4492047691525592 -0.0060175947850328

0.0298616118160560 0.3392836700564479 0.1905016164225811

0.0390794965940862 0.8259698772439776 0.1917428166303071

0.5320602480542942 0.3166921135932290 0.1938747629459774

0.5402810031742665 0.8439582713143087 0.1919517383992903

0.8202164116173294 0.3559477401727725 0.1930975241081110

0.7891680430002129 0.8129980354388532 0.1903311518894246

0.2931645062709464 0.3388121292812414 0.1943215392402473

0.2905847655294025 0.8259449519319626 0.1911091457482572

Supplementary Dataset 2. The coordinates of the nickel/cobalt surface. The energy of the nickel/cobalt surface was calculated to be -334.25 eV.

Ni Co O H

1.00000000000000

11.8928003311000001 0.0000000000000000 0.0000000000000000

-5.9265986588999997 10.3212535711999998 0.0000000000000000

0.0000000000000000 0.0000000000000000 21.1420993804999995

Ni Co O H

8 8 32 16

Direct

0.0020894205744569 0.2548601597725503 0.0984272776448391

0.0020394958921642	0.7548199253108132	0.0984318005577628
0.5020531197406445	0.2548449821955910	0.0984372190427320
0.5020895106122676	0.7548734813822611	0.0984303466348794
0.2521959657822271	0.0045357495914881	0.0985022081683113
0.2522367398080568	0.5045678965552017	0.0984988937953776
0.7522490677265770	0.0045724036204668	0.0985119120424660
0.7522042550556708	0.5045461999681176	0.0984971104995648
0.0021027057229265	0.0046184799689256	0.0988348803275680
0.0021394575987586	0.5046132511499614	0.0988036885480549
0.5021016979397062	0.0046298134814588	0.0988287927636716
0.5021074812289770	0.5046344589114712	0.0988396072677553
0.2522635165361863	0.2545279489519659	0.0988603114628413
0.2522480354736018	0.7544987736424950	0.0988395098580996
0.7522501840546908	0.2545002586764614	0.0988388855017443
0.7522522152263301	0.7545080263490034	0.0988526945364610
0.0874814869880343	0.1764437662259839	0.0448041230083043
0.0874435892830046	0.6764562322780733	0.0447865165073270
0.5874331362022112	0.1764128826291835	0.0447819900544791
0.5874625433846498	0.6764553837049857	0.0447825988531084
0.0859475520175319	0.4333394279585039	0.0568155773380962
0.0858553366572520	0.9333360901062436	0.0568657951067842
0.5858824033492804	0.4333736197959114	0.0568516754164200

0.5858950139858101	0.9333589187197460	0.0568695250218035
0.3358163366075559	0.1796498975780891	0.0473742182293926
0.3358303264993781	0.6796544387870331	0.0473642724150496
0.8358299622231086	0.1796046293559470	0.0473583293244591
0.8358020944685731	0.6795943372739670	0.0473795956360715
0.3334158591786031	0.4142763128112146	0.0564273293051055
0.3334251881773120	0.9142670612809320	0.0564127315062239
0.8333929133147288	0.4142355559120997	0.0564085796426776
0.8334372201850947	0.9142804428115420	0.0564134857586431
0.1712473032524822	0.0943864012005212	0.1409958172382853
0.1712100545214781	0.5943442679426609	0.1409741847922782
0.6712345797896598	0.0943912639452945	0.1409854619284966
0.6712226353574926	0.5943698572086913	0.1409952912995460
0.1678795323972771	0.3293201815876905	0.1497818313545825
0.1678018805507935	0.8292415310144949	0.1497692507487354
0.6678152646312955	0.3292496470066768	0.1497725259505431
0.6678594906438540	0.8293164226246935	0.1497845752737766
0.4185226741492058	0.0767103364213137	0.1402448433641262
0.4185553241393287	0.5767388969977458	0.1402599988813683
0.9186242650806168	0.0767615559449933	0.1402537065112383
0.9185551099424355	0.5767241181542287	0.1402362425704526
0.4169942453782783	0.3329518273151812	0.1529230747347081

0.4169993324675837	0.8329916344344878	0.1529114635644604
0.9170204282128736	0.3329570890001656	0.1528908265891631
0.9169842287131538	0.8329573350078879	0.1529249156024482
0.0897021417613129	0.1772253290116830	-0.0010245962991542
0.0897141363753804	0.6772672989086412	-0.0010558382016733
0.5896638258022223	0.1772192955637339	-0.0010566285049994
0.5897020005253423	0.6772477192698968	-0.0010357277546353
0.3383591088759600	0.1947186317722840	0.0021865915784704
0.3383921877255243	0.6947033434014707	0.0021737091098428
0.8383567407917208	0.1946560422096862	0.0021702815260555
0.8383126569150657	0.6946409367723305	0.0021948566806050
0.1641582019237156	0.3140023770023608	0.1949832751980206
0.1641307054557563	0.8139832105795967	0.1949755660360848
0.6641345204576675	0.3139393821221829	0.1949711288639066
0.6641563515175566	0.8140001969921276	0.1949880696275479
0.4146755073744872	0.3312123795591836	0.1987438189749415
0.4146175074853246	0.8312038252385663	0.1987326223832672
0.9146834265103880	0.3312384446464539	0.1987202263303102
0.9146596935088939	0.8312125527326386	0.1987576974761889

Supplementary Dataset 3. The coordinates of the nickel surface. The energy of the nickel surface was calculated to be -310.36 eV.

Ni O H

1.00000000000000

11.7038002014000000	0.0000000000000000	0.0000000000000000
-5.8513176253000001	10.2171014480999993	0.0000000000000000
0.0000000000000000	0.0000000000000000	21.1695003509999999

Ni O H

16 32 16

Direct

0.4878463888018887	0.4781900120348994	0.0985064257611583
0.4878557075601025	0.9782011251135619	0.0984619284972471
0.9878430056118993	0.4781791766496397	0.0984671234646436
0.9878971076928210	0.9782053371783233	0.0984639406616589
0.4881660653713368	0.2283263419941683	0.0983856026912230
0.4881423247781095	0.7282932107097136	0.0983716907680829
0.9881465355505785	0.2282921699426879	0.0983722771914860
0.9881534360273053	0.7282971128533459	0.0983538488355091
0.2378191036367313	0.4781705054704504	0.0984939954124392
0.2378330548526298	0.9781962628647434	0.0984740132998843
0.7378411618574902	0.4782044730415342	0.0984857043161936
0.7378229664020904	0.9781803578813620	0.0984768888616312
0.2381832289914735	0.2283358018406289	0.0983810077287135
0.2381663765000271	0.7283288017729819	0.0983686558300125

0.7381669508949631	0.2283252565205354	0.0983695247524360
0.7381934108377868	0.7283465436707287	0.0983675295198296
0.1450595943973926	0.0512514600870599	0.0456210892682964
0.1450104816319899	0.5511936453681627	0.0456488279872198
0.6450265655796305	0.0512207324716818	0.0456330447782780
0.6450617458348653	0.5512363815245359	0.0456556444212719
0.1577035529730824	0.3163151070436410	0.0576607379840032
0.1577362796137404	0.8163242014863851	0.0576385628455851
0.6577176533673761	0.3163340594849161	0.0576641562158627
0.6576904197989974	0.8162870974123327	0.0576510499991206
0.3950186683377306	0.0511947260988741	0.0456179888844416
0.3950086862892688	0.5511232249813489	0.0456479116073910
0.8950229202185767	0.0511665102154295	0.0456343700815925
0.8950275019258821	0.5511752781401991	0.0456244197524045
0.4078007990562035	0.3163585564793431	0.0576923037142366
0.4077667781167872	0.8162867966496403	0.0576456207523129
0.9077509172939519	0.3162856186097639	0.0576547223141165
0.9077862872702729	0.8163275779741013	0.0576466170371620
0.0684065315813115	0.1401950308594773	0.1390557218526026
0.0683517978480459	0.6401952813073566	0.1390565966806669
0.5683718868004330	0.1401960976526044	0.1390671413667144
0.5683868595134632	0.6402080769263288	0.1390697494045199

0.0803133204431785	0.4051884560426730	0.1513767162514296
0.0802598473685432	0.9050745314598559	0.1513809930224894
0.5803590116272646	0.4052350761773071	0.1514068539895249
0.5803519141640555	0.9051884018794853	0.1513727404353595
0.3184050212546687	0.1401618827136494	0.1390695731871532
0.3183869258816074	0.6401776587121484	0.1390712114937388
0.8183844159972490	0.1401675711687158	0.1390672500751688
0.8184150828776597	0.6401873899027999	0.1390641561253069
0.3302402659878132	0.4050771448227087	0.1513911092012769
0.3303860850742050	0.9051823415076937	0.1513408697212193
0.8303646960972518	0.4051703503284265	0.1513519404992207
0.8304135607195759	0.9051978737856857	0.1513375321468527
0.0971395674686136	0.4287746070757296	0.1957024466043781
0.0972156836156879	0.9288037493568758	0.1956900518314325
0.5971906455223354	0.4287966212338996	0.1957325472023264
0.5971448821907783	0.9287743619249319	0.1957009037179218
0.3471517570907853	0.4287253698946075	0.1957131669445738
0.3471448433161601	0.9287009616913445	0.1956761713605886
0.8471421331854654	0.4287134400264154	0.1956811441941994
0.8471594484994470	0.9287037954485737	0.1956741878835150
0.1288627880313839	0.0265020878450953	0.0013957028810662
0.1288816099890493	0.5265019222042092	0.0014122976045146

0.6288708900932373	0.0265008610297049	0.0014000584393859
0.6288763604859937	0.5265068652303805	0.0014364257829719
0.3788484414711028	0.0264772031553639	0.0013874408026427
0.3788146063980301	0.5264349888024715	0.0014113251382885
0.8788021194095954	0.0264456671827008	0.0014057652069951
0.8788174997369071	0.5264427558439121	0.0013967360054262

Supplementary Dataset 4. The coordinates of the glycerol adsorption step on cobalt surface. The energy of the glycerol adsorption step on cobalt surface was calculated to be -431.31 eV.

Co O C H

1.00000000000000

12.1820001601999994	0.0000000000000000	0.0000000000000000
-6.0910000800999997	10.5499216077000000	0.0000000000000000
0.0000000000000000	0.0000000000000000	21.1058006286999991

Co	O	C	H
16	35	3	24

Direct

0.9503461536430058	0.1519215721425552	0.0922984950537353
0.9549943768890132	0.6522521836743390	0.0977034971426609
0.4522806577788442	0.1490335165197944	0.0985437155607171
0.4522453890888914	0.6530069215220782	0.0880008911475717

0.9494369261299301	0.8969948891892419	0.0950246949165004
0.9489788231207236	0.4044890932290788	0.0940601170218068
0.4570354779625099	0.9017826664062965	0.0940599535313415
0.4461591515178847	0.3963834178904690	0.0940196701713052
0.7059791499355058	0.1530259852170625	0.0927367689838278
0.6978122729417170	0.6509489474094483	0.0892989909306452
0.1985949715959294	0.1487412059492673	0.0996792026045617
0.2030205483064597	0.6555724067541290	0.0928410389353706
0.6980665464011785	0.8964755919508763	0.0876128992969725
0.7030410990526931	0.4058420187772060	0.0991373451410260
0.2060974265310876	0.9034198362383495	0.0988562869743555
0.1970567076345466	0.4024490899363714	0.0930648133516674
0.6567683353969112	0.7930535627406521	0.2563856775868867
0.4960176626173596	0.4942899265059332	0.2533392749247530
0.7271597775547435	0.8863960879949137	0.3714665476795876
0.0327379157644155	0.3347176058700131	0.1425632054448224
0.0364448882635353	0.8201185273984958	0.1499652411049116
0.5348271782077756	0.3183221516677899	0.1487447664513449
0.5347058585087140	0.8139569471142912	0.1422261431822278
0.0317252999890692	0.0607712845417792	0.1379641752998089
0.0279509867678241	0.5632341005889260	0.1375302856937378
0.5397272797101662	0.0603143787879103	0.1358651485533328

0.5252350981646543	0.5545944485106489	0.1363642694515164
0.8014140391391270	0.3329727827552421	0.1469605585596302
0.7810651828437797	0.8156670033977705	0.1420765975138930
0.2829764335664157	0.3202561576388989	0.1482142052678740
0.2965309360081199	0.8223049943383549	0.1473768329392108
0.7837160107133263	0.0778478726921132	0.1334210181744565
0.7752188287214259	0.5612893219567616	0.1378565400618892
0.2855531074945915	0.0604175765707100	0.1426182436379714
0.2785568810993559	0.5616265626620822	0.1306155849250801
0.1163921808594465	0.2435917401737838	0.0543890377589700
0.1284728137284435	0.7451418557242352	0.0535879545918151
0.6291834046687846	0.2378456672737608	0.0568360872467347
0.6110630744561725	0.7186974780046900	0.0436345195515158
0.1193194634279050	0.9813706508028101	0.0456478207411158
0.0988412984348823	0.4785424641907777	0.0424015662390333
0.6108804784170618	0.9789039921166628	0.0386442750754386
0.6120944022301620	0.4731006740403821	0.0416793233465262
0.8681914543539100	0.2482073615259119	0.0505522705683335
0.8777613640771532	0.7389715095525831	0.0517644131835054
0.3665281548243474	0.2331519464550181	0.0544584821720010
0.3759294593601548	0.7490977675483755	0.0497234640816746
0.8723922040915104	0.9855081987464654	0.0411385607310380

0.8678752909818707	0.4770987037584716	0.0455385373241749
0.3707167084386981	0.9839808778229759	0.0453818175073508
0.3570711314232754	0.4729316800520007	0.0409739031361696
0.4892667102157185	0.5845464972519604	0.2925195217495135
0.6097990360444118	0.7671996275126965	0.3677052638232616
0.6171056154222797	0.7018785164323373	0.3076200159578664
0.7144903625080092	0.8751254123305282	0.2761822216178171
0.5131914910925545	0.5270721568232157	0.2075801359012116
0.4267823311992650	0.6171864738097126	0.2719781762592492
0.4467142632216246	0.5371741595543507	0.3378997923335590
0.5283697937883404	0.7832291445674577	0.3648226061401518
0.7145730987642870	0.9447927240389712	0.3975747314938665
0.5981038606156018	0.7076889393210585	0.4093747274460386
0.6888775334905403	0.6725980948243552	0.3135528711366014
0.1209055829865963	0.9799565146027401	-0.0002869360081650
0.0847497578573271	0.4609996354830375	-0.0027028898461369
0.5955760394860680	0.9658175074858193	-0.0066043773230610
0.6126339764989026	0.4657909697188665	-0.0040854658543514
0.8774642088537201	0.9872760296433856	-0.0047636813201048
0.8633427090914604	0.4603291122628020	0.0003341164720439
0.3639245591663997	0.9788945436305513	-0.0004665351815079
0.3462098336986950	0.4561127027464524	-0.0042723272126789

0.0459599511055517 0.3603282826287768 0.1868453082779261

0.0308482784984954 0.8235336535976684 0.1957075760239326

0.5323867725385428 0.3271576291552306 0.1944790563383933

0.5520570163427972 0.8116128272238398 0.1872961008627539

0.8187743175253551 0.3501598859331183 0.1918947296961452

0.7572771649205784 0.8039975007807560 0.1870639658914569

0.3029151229074664 0.3365312567656014 0.1932020985801929

0.3020833598504092 0.8279960965695436 0.1932369672061224

Supplementary Dataset 5. The coordinates of the glycerol adsorption step on nickel/cobalt surface. The energy of the glycerol adsorption step on nickel/cobalt surface was calculated to be -411.50 eV.

Ni Co O C H

1.00000000000000

11.8928003311000001 0.0000000000000000 0.0000000000000000

-5.9265986588999997 10.3212535711999998 0.0000000000000000

0.0000000000000000 0.0000000000000000 21.1420993804999995

Ni Co O C H

8 8 35 3 24

Direct

0.7512157359649088 0.5045098671036713 0.0975583146079619

0.7519734888495795 0.0047929362935232 0.1009136590353110

0.2519523507889269	0.5032056668584399	0.0971638556934776
0.2516945483943184	0.0056572540589439	0.0990803661413181
0.5016202900435321	0.7542061457365929	0.0983533733052973
0.5018400822117645	0.2558971870469977	0.0938864022372503
0.0008625724996370	0.7542229902788862	0.0999314137707426
0.0031638874898804	0.2544455098753497	0.0994224136036818
0.7519502827822934	0.7564034771902377	0.0962507092845622
0.7514435199867914	0.2545363367884206	0.1001958638070090
0.2516198617357106	0.7543945004752350	0.0998259644702763
0.2522096465446682	0.2540399626525421	0.0969465911061755
0.4994684081577019	0.5017961677519693	0.0939212361852218
0.5015167294421151	0.0048004467057254	0.0997260768970039
0.0020544965208566	0.5045983719892987	0.0998514227452778
0.0032366029765199	0.0055446761564070	0.1009660554270547
0.4957015159199606	0.2111660692276593	0.3680311927037929
0.5237083955145131	0.3327901643799254	0.2589480114285470
0.6712003378397681	0.6275686287020573	0.2623666159666210
0.9110985691503709	0.8292915225507901	0.1529677793178562
0.9144231412391896	0.3294271428629454	0.1537065349894517
0.4137259179845666	0.8290189953874602	0.1535992607867022
0.4076091517342548	0.3278380988167450	0.1450231697912781
0.9172810170187731	0.5757429522858684	0.1415625230637375

0.9205710848622169	0.0775277643168002	0.1425226651437224
0.4186722964556720	0.5734255025255801	0.1376114624960075
0.4198524513589115	0.0797699795256211	0.1400031246301080
0.6644392147286051	0.8291101966293116	0.1514222810488779
0.6623503519109559	0.3331629985589641	0.1485809622655892
0.1661458797575700	0.8336817751998167	0.1516795974366189
0.1729569271777739	0.3276648492056518	0.1473028259656518
0.6667879867518582	0.5894488669598217	0.1399226258911409
0.6679574011031814	0.0904565194216583	0.1431746010175118
0.1675710943004735	0.5893870195891638	0.1422975139981836
0.1726267960018069	0.0957705664788461	0.1414245254375207
0.8352222384531287	0.9213240585413685	0.0564770539332519
0.8362232933007773	0.4194407790485160	0.0564516695720841
0.3361730571173769	0.9190769350767560	0.0565895890134124
0.3309271527500623	0.4126625735493075	0.0530270599616703
0.8387551022173536	0.6779209269797817	0.0458957156325459
0.8384158120984211	0.1767873692421305	0.0483902073719441
0.3365210443618502	0.6764852690480165	0.0462941229983423
0.3313366548338465	0.1790530119425822	0.0461562058229808
0.5855964777304965	0.9337225623673939	0.0579183519742392
0.5857970335339003	0.4350891706437596	0.0517958081709128
0.0841372494072495	0.9332337672819364	0.0582624885972445

0.0835025917737751	0.4315383353866904	0.0570838093103281
0.5893944515241335	0.6794722610732912	0.0428684945304492
0.5912432178828428	0.1776339402808722	0.0442237258210339
0.0894436432524311	0.6788400391076496	0.0461678928639117
0.0974513526295391	0.1814904348311702	0.0474559798680239
0.6096816433181796	0.3376809036926748	0.3623086660244340
0.5778507751225407	0.4133900147886401	0.3142718141515651
0.6983951099512052	0.5408438510603178	0.2958238698221756
0.7488473802566247	0.5894706461603123	0.3399322638181530
0.6348242251385396	0.3879382120256678	0.4083173663183338
0.5222429561611414	0.1488939650563849	0.3796640475058143
0.6926458714141552	0.3289008418483126	0.3451999193311063
0.5047236531720060	0.4349042407114273	0.3341634669032513
0.4727697625811714	0.2439428349137971	0.2752063709890380
0.7641949399459811	0.5183459684585608	0.2684907768666080
0.6620237513105155	0.6041287776659433	0.2160206756967261
0.8962922010072882	0.8233481632355789	0.1983238646918562
0.9096629053981686	0.3338346182788290	0.1993162997007479
0.4112806675818815	0.8292811078470438	0.1993953826363067
0.4143369894691462	0.3290097354328241	0.1912533945139317
0.6650025925478715	0.8168609989043998	0.1967900130613087
0.6416538605109093	0.3302195645203614	0.1934983477448863

0.1676231341513855	0.8305096847718176	0.1974130582849758
0.1772679290116978	0.3155907830575134	0.1924373669718593
0.8379012565638301	0.6785405755674070	0.0000947566810746
0.8349884724060940	0.1779872910968741	0.0026422491179616
0.3345758672841070	0.6821478424034615	0.0006445708736368
0.3272558546741672	0.1888993094344230	0.0008173870123294
0.5938986216366930	0.6796966347868704	-0.0029040771122668
0.5962828209228233	0.1690007626074166	-0.0011148692081662
0.0918986568529232	0.6740287916974591	0.0004765823581309
0.1080542708495257	0.1860360636584671	0.0018701280249110

Supplementary Dataset 6. The coordinates of the glycerol adsorption step on nickel surface. The energy of the glycerol adsorption step on nickel surface was calculated to be -387.95 eV.

Ni O C H

1.00000000000000

11.7038002014000000	0.0000000000000000	0.0000000000000000
-5.8513176253000001	10.2171014480999993	0.0000000000000000
0.0000000000000000	0.0000000000000000	21.1695003509999999

Ni	O	C	H
16	35	3	24

Direct

0.7377825285265307	0.7279401816194360	0.0982427091952296
0.7377283883752896	0.2278893622226954	0.0984812049660136
0.2375371879803959	0.7282146946141980	0.0983268433731894
0.2379135436189369	0.2285372037277853	0.0978477295318737
0.7372462752774445	0.9771969932649083	0.0985394025920364
0.7372755830919264	0.4777148904278021	0.0990848499636198
0.2374578617798541	0.9782538339069172	0.0987598935766954
0.2375408839340739	0.4779641655137998	0.0985808526800190
0.9875193938153427	0.7280744095595532	0.0987966302543132
0.9877668308065992	0.2280410406615049	0.0985662810379051
0.4872454372041892	0.7280471616437525	0.0976707957921471
0.4879016580693254	0.2284526504847288	0.0975773371452686
0.9874647943938508	0.9780380105134109	0.0989704651237500
0.9875577979959007	0.4781946428466304	0.0989018524560200
0.4872930411768583	0.9777292617063634	0.0986808570886112
0.4860718302805023	0.4755265341877031	0.0972414288362149
0.4475913655928005	0.2754221804917119	0.3709176391550692
0.5791449346688630	0.6827363311087042	0.2625007797835400
0.4541994337706988	0.3942412499442269	0.2620828347589310
0.8287791318603901	0.9037367514242196	0.1514772976322933
0.8299670679509434	0.4049999588478150	0.1516144158668269
0.3299159647617310	0.9048868789116240	0.1514406195827933

0.3299904888553387	0.4031858718232826	0.1500206845677080
0.8174133353063744	0.6403059882512228	0.1396326179598178
0.8179188819677561	0.1395278098990260	0.1391681088344456
0.3183869019465468	0.6406314265286861	0.1387143009099565
0.3184811591601850	0.1408283022904869	0.1388722775847398
0.5787969858116249	0.9021499931886142	0.1508902681915774
0.5771584633078036	0.4013571148093656	0.1495193262047578
0.0798244145155891	0.9051889646112673	0.1517392203517013
0.0804847538398723	0.4051605118938079	0.1515022666847769
0.5671832917793256	0.6387382273070065	0.1386395738404685
0.5678853330612860	0.1402910188621790	0.1389925909204139
0.0680247803135652	0.6402730201692649	0.1394277993045987
0.0685037432862037	0.1403794209917883	0.1391734220181551
0.9073560315799174	0.8161256930206167	0.0580698576912652
0.9074426700665675	0.3162150369237213	0.0581047007873497
0.4068522030060548	0.8160357527618984	0.0572047300980236
0.4066173535559308	0.3151554729188449	0.0558178502906191
0.8946599415219395	0.5514099507833277	0.0460366599563520
0.8946952782126889	0.0506067370059031	0.0458749912102814
0.3938628649881562	0.5509736120397548	0.0451719449972771
0.3943297351840390	0.0501367703249931	0.0457118571128926
0.6575072621046349	0.8157496449927012	0.0569993859040647

0.6583242006862042	0.3163190912470734	0.0572966841327910
0.1569385685791211	0.8162684170149735	0.0580427446708921
0.1567069994207317	0.3160376883095279	0.0574153945428244
0.6448525367065041	0.5509572475925443	0.0456511916473809
0.6443080790594938	0.0500899704973514	0.0459003864274433
0.1442882574885448	0.5510512527775792	0.0458299743993765
0.1443734651699460	0.0503211608383906	0.0457875763963194
0.5586601252999153	0.4047541406120978	0.3614616588228888
0.6242377397289051	0.6080009080265207	0.2952301736125715
0.5124467312513016	0.4753354677036261	0.3169937543960640
0.5914458655178632	0.4579516419091857	0.4066997178878681
0.4796769033455843	0.2166103462913235	0.3829045109477718
0.6415071678611082	0.3989848506415616	0.3398634198238588
0.4367657565375438	0.4900359010543585	0.3408025623933972
0.6776681944191758	0.6635063947331760	0.3377189221697226
0.6941264986986611	0.5925954623848854	0.2661786285349274
0.5707597512779804	0.6600465511368390	0.2162808112161316
0.4042641687113410	0.3037453740244248	0.2772445035568163
0.8784083634448026	0.5267607861990452	0.0017972375858052
0.8790578842301084	0.0250902631057926	0.0017407329841778
0.3762553297859217	0.5271727552546908	0.0008937636568883
0.3776355603503255	0.0241093427623220	0.0016343985085626

0.6312444708285400 0.5279173775516952 0.0011800512745601

0.6276536039082838 0.0245216999753994 0.0017802034667340

0.1273862025572569 0.5259984109796851 0.0016652875888212

0.1280038073265822 0.0244431966285466 0.0016918658721626

0.8385923599746724 0.9213322649639846 0.1965425607876547

0.8451019192034858 0.4289235474082039 0.1959444634463706

0.3463244240989302 0.9263143067946327 0.1960096360297713

0.3543087380864210 0.4138208110352261 0.1948629336342568

0.5913771635357988 0.9126107011751179 0.1963209253281231

0.5672349209535187 0.4084647420329767 0.1950926707385663

0.0951625866158632 0.9292455448256588 0.1960433502140427

0.0988954778566189 0.4282054006663308 0.1958366084552532

Supplementary Dataset 7. The coordinates of the glyceraldehyde formation step on cobalt surface. The energy of the glyceraldehyde formation step on cobalt surface was calculated to be -433.61 eV.

Co O C H

1.00000000000000

12.1820001601999994 0.0000000000000000 0.0000000000000000

-6.0910000800999997 10.5499216077000000 0.0000000000000000

0.0000000000000000 0.0000000000000000 21.1058006286999991

Co O C H

16 35 3 24

Direct

0.9535637192584018	0.1524501801769008	0.0897556618124430
0.9590494887566229	0.6609623691783895	0.0933081245667533
0.4569003846830176	0.1534505282779690	0.0872556232907814
0.4512729110992548	0.6477917316100490	0.0965992470424637
0.9530133669708303	0.9026087769466259	0.0928345791512480
0.9472440488620545	0.4028099404407325	0.0982248383662497
0.4500458260512189	0.8962973719787458	0.0921921566212507
0.4464626005386128	0.3951673027128929	0.0908545003036399
0.6996335340948513	0.1494313864225213	0.0914602482401455
0.6989816810824792	0.6579993070589106	0.0906287299755486
0.1976421414796946	0.1489395666247565	0.0903919474282882
0.1974143512172613	0.6552991608825663	0.0954509099260307
0.6991919695455803	0.9002120128944118	0.0886263341513958
0.6992148386771760	0.4002615483754468	0.0956296423831074
0.2064960067420109	0.9081374868005810	0.0906265099190409
0.1924493446517608	0.3904009781899556	0.0959503013226539
0.6704100910014801	0.8066307551309088	0.2661257647366003
0.4873305579110490	0.4798901752608389	0.3078387612462299
0.6554252600253238	0.8496102718877059	0.3922770726264573
0.0277918909839415	0.3074838159798140	0.1491607406253563

0.0468377996096074	0.8355366286404653	0.1443062473127882
0.5292279553013043	0.3162057775601209	0.1452974962001798
0.5331895273939264	0.8142932731228176	0.1471448255604102
0.0273386559555471	0.0547318722175657	0.1360980714036447
0.0331400504253788	0.5688818972796440	0.1386921641070470
0.5358534938062849	0.0538640360601293	0.1302754635854815
0.5272825084172896	0.5629075414851804	0.1372502681394775
0.7941136715794470	0.3313132469746649	0.1489882707204781
0.7875036648156508	0.8245063614042987	0.1431654468781481
0.2843765206755512	0.3118066002239822	0.1455163574191998
0.2946756842401937	0.8334747523635688	0.1448062393117806
0.7770510546924961	0.0592831251985614	0.1264819722850886
0.7826244758486127	0.5742440423547320	0.1449351490201621
0.2869040628729852	0.0657016332031787	0.1303201870595402
0.2777462721790027	0.5615694204618346	0.1461807944335934
0.1106033377208359	0.2237393213914450	0.0466405787020831
0.1263623475395130	0.7495227337838576	0.0513484499086722
0.6206366193154630	0.2306838488385546	0.0511912358272718
0.6202048764754396	0.7390359081922352	0.0482931059071731
0.1146117147277964	0.9792659950514999	0.0395317659041423
0.1076229810516980	0.4790321108559901	0.0480826826112170
0.5977532471731303	0.9548534565913195	0.0368711262473461

0.6201392754392381	0.4908107669318769	0.0431156273157779
0.8666274851141407	0.2490629407103221	0.0544480234082350
0.8737910585974120	0.7405958572287958	0.0535287784826524
0.3745596445386529	0.2464293382346700	0.0451509209880761
0.3630592780153991	0.7300478474825126	0.0556536241876889
0.8706284582782765	0.9769192992073983	0.0382009285814772
0.8672055201946200	0.4963506581349085	0.0445824214703912
0.3642251888488929	0.9777123797086843	0.0382521172164875
0.3603778936389331	0.4830947013331434	0.0443484340354774
0.5411899537145555	0.5796940849688060	0.2781308746752549
0.5640217203344815	0.7278857580839799	0.3670739697072117
0.6290070212694379	0.7050374019024372	0.3111092552157922
0.7083625109283597	0.8849033192438388	0.2909523040797533
0.7825227355749378	0.5798689490899358	0.1907136176186281
0.2767478007168342	0.5656774153620551	0.1920049300824331
0.5311025214234237	0.5834541557520317	0.2253834477207077
0.4784369364254540	0.7285376740656424	0.3504847659894600
0.6113554991515250	0.8825368681705741	0.4173015208924277
0.5365788834834305	0.6514855365560746	0.4020570529265539
0.7117565144490278	0.6998867749250288	0.3292160629356342
0.1091501654378661	0.9703934272981339	-0.0062377860279667
0.0950520751462776	0.4682889449466557	0.0025457985353386

0.5735276647101399	0.9082397867990735	-0.0030038586421206
0.6213842209368858	0.4883473370663149	-0.0028192780045554
0.8674285599385317	0.9624112931562988	-0.0071823349965109
0.8694834523325767	0.4945231570597220	-0.0013381603516750
0.3516284392810486	0.9645861206634654	-0.0071558936676807
0.3632040327530167	0.4852595809504403	-0.0015921610266857
0.0262152341603335	0.2925699242991924	0.1943562629968099
0.0588070922300165	0.8636379296412471	0.1881218231044571
0.5147874293307169	0.3071462501462030	0.1906803382505507
0.5541043579803828	0.8235543202424260	0.1920568455939886
0.8115720779052329	0.3503613928641393	0.1937570025033944
0.7740867660224792	0.8311231935385088	0.1883587673570617
0.2892276153376678	0.3048232435552024	0.1911110237889775
0.3122478169098766	0.8615049685900836	0.1885958448128818

Supplementary Dataset 8. The coordinates of the glyceraldehyde formation step on nickel/cobalt surface. The energy of the glyceraldehyde formation step on nickel/cobalt surface was calculated to be -413.30 eV.

Ni Co O C H

1.00000000000000

11.8928003311000001	0.0000000000000000	0.0000000000000000
-5.9265986588999997	10.3212535711999998	0.0000000000000000

0.0000000000000000 0.0000000000000000 21.1420993804999995

Ni Co O C H

8 8 35 3 24

Direct

0.7516792210149403 0.5057635037735815 0.0989362809468533

0.7511162774951394 0.0049232210687348 0.0990793664483673

0.2460889246696851 0.5043504203934405 0.1000271545221851

0.2531506229292179 0.0089312207305313 0.0981132584861479

0.5030706261430462 0.7600239788564811 0.0982734652777658

0.5030640514941428 0.2551476766491557 0.0975194599750117

0.0055344391793953 0.7652875395174730 0.0975384440070865

-0.0005293636304161 0.2532521557481411 0.1026551810942080

0.7512715322808421 0.7570787309939481 0.0974756131902125

0.7518223468653277 0.2531679605383209 0.1042529648434335

0.2515251742610149 0.7592800053713702 0.0964139769247932

0.2512675548935968 0.2555374084235111 0.1002178387881721

0.5020078147887180 0.4957269043805582 0.0911169204087621

0.5020982478676480 0.0063205232241904 0.0988276201987499

0.0047717193969520 0.5047424721663810 0.0993168724460022

0.0015778157932786 0.0069841906408140 0.0991067705379116

0.9228235699898407 0.8515514535466694 0.1493879681069396

0.9099419190068311 0.3246354713406035 0.1592639938585916

0.4220608597421311	0.8506088032366101	0.1488528560066684
0.4174897944867556	0.3361932661034965	0.1498615705881745
0.9194910466461380	0.5911342726392628	0.1448724555659148
0.9146839364861852	0.0773331529779889	0.1430466508545415
0.4140407752422641	0.5863608033329850	0.1453091351759121
0.4176507248767869	0.0809864240684603	0.1416740026727695
0.6662594666341732	0.8325779832418236	0.1514757877167902
0.6649371778996646	0.3358853718401686	0.1531197554564564
0.1680826135781015	0.8361281652428091	0.1508514470638998
0.1647481378231914	0.3313534403831669	0.1546986731823550
0.6770040503641150	0.5944842895972754	0.1408330976118030
0.6618655479508004	0.0869980628662433	0.1447673855026211
0.1696836448210872	0.5927645295140630	0.1414415401541330
0.1645268441942629	0.0904671446160722	0.1431629209311706
0.8385675772144801	0.9210735825131677	0.0547546916396964
0.8330280106105534	0.4158863800788386	0.0609071542952471
0.3401824172624108	0.9235927632828858	0.0544340978794442
0.3225817389942505	0.4168063524968826	0.0565185665691538
0.8402823565484842	0.6786362026487743	0.0468681354888428
0.8365794882017807	0.1780069506425322	0.0493414237666872
0.3370773300356532	0.6775637088647619	0.0473628048903833
0.3376584321459797	0.1803190490596425	0.0461071748713985

0.5871923145665923	0.9329629362605358	0.0555718461350707
0.5936278580478357	0.4286977933785058	0.0514276655754230
0.0885242168185546	0.9369043905217036	0.0550732458044490
0.0835852872672999	0.4242186117898935	0.0578177473623630
0.5831922167731672	0.6735729720408220	0.0458577745580217
0.5827765003193262	0.1617372133878877	0.0497280833312074
0.0887382035629554	0.6838283959173906	0.0458196627629499
0.0792990093586384	0.1621738271499173	0.0500431447937669
0.6509394467858092	0.2917001317846699	0.3684750291523672
0.6902995915754090	0.6663495846415846	0.3159114479890688
0.5249665956030286	0.3357215465445245	0.2714993622841295
0.5958937321766870	0.4349937026955274	0.3172640940951044
0.7079820943816544	0.4209130263217314	0.3436705370808184
0.6481533290273623	0.5662019431660917	0.2851244596561970
0.9161064652056204	0.5818894966466013	0.1905096489094669
0.4068053352754316	0.5830440753208552	0.1910301958638035
0.9255777933933655	0.8742986457072569	0.1937363167413046
0.8970086416683248	0.2991736577211636	0.2033681782701562
0.4262671173651060	0.8735700773902322	0.1932200304123363
0.4316208749699333	0.3391492055361089	0.1954138409005875
0.6776535622058180	0.8380952203610468	0.1969838589795122
0.6411787608361611	0.3269749662505753	0.1976026260419454

0.1796344707307630	0.8451687909169476	0.1962356450879548
0.1594312383503569	0.3132150878104070	0.1996023481495770
0.8400205989742471	0.6808094353037988	0.0010752798996105
0.8359261925839855	0.1869879687017652	0.0038231411855612
0.3356602555134380	0.6783581116793368	0.0015526945138381
0.3314252624863798	0.1796531658550104	0.0004265504575896
0.5868079547208589	0.6802678184411119	0.0001204021396626
0.5840191671546496	0.1431151975645127	0.0050505029181442
0.0920973809524493	0.6836476216344722	0.0000557285401707
0.0754315451316602	0.1434791751489468	0.0050820101535067
0.7580797890064657	0.4941680106188958	0.3808056707487608
0.7134459676395107	0.2619779436816515	0.3656672200866843
0.7772683293648854	0.4358685341668253	0.3053868855346262
0.5323201390685558	0.4287175148493576	0.3568470656376871
0.6490399314847120	0.5662070765957159	0.2319616462889064
0.5059885554988663	0.2534050940440603	0.2907006856210096

Supplementary Dataset 9. The coordinates of the glyceraldehyde formation step on nickel surface. The energy of the glyceraldehyde formation step on nickel surface was calculated to be -389.31 eV.

Ni O C H

1.0000000000000

11.7038002014000000 0.0000000000000000 0.0000000000000000

-5.8513176253000001 10.2171014480999993 0.0000000000000000

0.0000000000000000 0.0000000000000000 21.1695003509999999

Ni O C H

16 35 3 24

Direct

0.7364202337762542 0.7302531356493968 0.0995360746159621

0.7358571640287052 0.2267929374289884 0.0962328477062403

0.2355533812243081 0.7300901320004619 0.1007363525005241

0.2357355593896180 0.2266606473610808 0.0952206901435515

0.7369711310616791 0.9780345075402731 0.0970900330835512

0.7335069726316266 0.4718356580284385 0.1022498938485132

0.2370066459273605 0.9783841125091274 0.0970281570081638

0.2333492258931420 0.4707005408181092 0.1017035023672723

0.9899682768573603 0.7298795632575550 0.1003268126729337

0.9861706277067612 0.2270840889252249 0.0961881181020657

0.4895684737760366 0.7299641433332544 0.0995792083159721

0.4861224983410546 0.2273882963098693 0.0959904699115835

0.9868501699955876 0.9781986886521384 0.0973073872353477

0.9866450485047832 0.4775297225743294 0.1016184803943561

0.4868096216658491 0.9784143393951444 0.0978215198885806

0.4862003181393677 0.4763578830285032 0.0988197072829249

0.8290574905556478	0.9069607449152626	0.1517766617515919
0.8261876132054552	0.3970137112700132	0.1532987615248098
0.3299485576702532	0.9082312966137457	0.1521409686949156
0.3257185657585367	0.3953461811469940	0.1515131371137216
0.8223206729428320	0.6497101364185680	0.1472076989893590
0.8175002902100156	0.1400785568964873	0.1377969454310173
0.3210350921460268	0.6484901444078568	0.1467927434914801
0.3174435379031959	0.1404831623873136	0.1374375584069156
0.5794235903231155	0.9057216743651884	0.1515870291480591
0.5661474675715642	0.3942203501660440	0.1531087632652414
0.0799325243658878	0.9079138987325983	0.1517705300996170
0.0681930862004470	0.3928866111330222	0.1542878128888246
0.5688700641156483	0.6428459327860858	0.1412923589307307
0.5667254266944757	0.1395140135123530	0.1380028834374736
0.0689411835204050	0.6438953776353841	0.1435246571315137
0.0668748148581727	0.1390590023599993	0.1374760849085740
0.9059721247464976	0.8143156311008756	0.0580789933206544
0.9080073297400363	0.3203824680850854	0.0563603851788059
0.4049155863448863	0.8145030919345505	0.0584623380431237
0.4075351593723198	0.3191398357215694	0.0544776106977235
0.9015614089073319	0.5597919454486388	0.0504853810493390
0.8938763801688798	0.0484056098942660	0.0439416098400308

0.4010788312084773	0.5597863234789684	0.0494281107946306
0.3940954333271117	0.0487110572844868	0.0441168889556426
0.6559343262993989	0.8144999716550961	0.0562314211358985
0.6506788038938418	0.3062541146043904	0.0546721543558552
0.1556084501163744	0.8139008484857693	0.0571429250248928
0.1497619812628025	0.3054176016629789	0.0538047588601220
0.6518742876603911	0.5575595735391650	0.0498968254115176
0.6438838147444090	0.0488402554697990	0.0446102974040738
0.1484042906757398	0.5556414370273405	0.0513705537536231
0.1437490184286555	0.0481038323570570	0.0437816602766668
0.5798375487900842	0.3580744426696918	0.3682338615797752
0.4388973840350790	0.3950897562381475	0.2755001193288206
0.6196055812640596	0.7334215682666827	0.3127601210923148
0.5183451187244754	0.4994291540227752	0.3181138899085647
0.6354196208239744	0.4858917930379538	0.3404696040923632
0.5672794353636198	0.6293470485035501	0.2840957703490208
0.8317840004556513	0.6681123787433838	0.1921963118368048
0.3258630625588363	0.6625295603153971	0.1921524598247921
0.8977371410539990	0.5507570754440405	0.0049263700022388
0.8801250863365765	0.0252988105756559	-0.0005298285341288
0.3968991053463465	0.5520160720877395	0.0038092404817173
0.3808315942237676	0.0261352180783124	-0.0004183960727357

0.6440122796736759	0.5438868103656281	0.0045865011324966
0.6291397744813708	0.0279883573630508	-0.0000675224487019
0.1348675615539005	0.5396012056616722	0.0063117407415480
0.1290432378493380	0.0269674646931552	-0.0008590397638406
0.8447604766687037	0.9407973429642837	0.1946447014794622
0.8377370395016621	0.3951642286221299	0.1985205343008883
0.3489805148889334	0.9459408604572147	0.1942393146457138
0.3403738867247350	0.3912574833703091	0.1965557380830238
0.5994856916452004	0.9334686629081137	0.1953267690884993
0.5416761744612385	0.3894314997310274	0.1973893165511715
0.1010856987421394	0.9447557575447438	0.1940210848328179
0.0633190346497458	0.3847039904450410	0.1998523923482822
0.6409968258502119	0.3255519810385445	0.3630523983572898
0.6934665010990516	0.5635014673320395	0.3750225140545757
0.6981393127607410	0.4951059922505995	0.2996122288955954
0.4604458920519541	0.4970340771220814	0.3598757609297250
0.4254091883946035	0.3144611162592807	0.2954976130811194
0.5565009998846386	0.6249209931364338	0.2312611735748689

Supplementary Dataset 10. The coordinates of the glycerate : lattice oxygen attack step on cobalt surface. The energy of the glycerate : lattice oxygen attack step on cobalt surface was calculated to be -436.62 eV.

Co O C H

1.00000000000000

12.1820001601999994	0.0000000000000000	0.0000000000000000
-6.0910000800999997	10.5499216077000000	0.0000000000000000
0.0000000000000000	0.0000000000000000	21.1058006286999991

Co O C H

16 35 3 24

Direct

0.9491590825789789	0.1486280062391318	0.0902242745673789
0.9508818428811276	0.6538964190724815	0.0997880892193325
0.4545965024666504	0.1474531984998758	0.0909384591653467
0.4486370791162094	0.6544558884932520	0.0916521436859717
0.9580772863741304	0.9119019031487801	0.0935563089193362
0.9440031834493200	0.3876756106429661	0.0946111393807400
0.4512487957409567	0.9009277938409800	0.0921107776190402
0.4471084066114551	0.3942609302955459	0.0952496935413534
0.6978652259963458	0.1470865825576140	0.0892917331964476
0.7215412628390780	0.6681369735026707	0.0828780413934096
0.2095877939099529	0.1562383398784560	0.0854731333299354
0.2041514996890853	0.6597350677337823	0.0939511226618997
0.6994209916696467	0.8985334015900516	0.0796326917596654
0.6941881490893379	0.3948671111701223	0.0972809840845316

0.2049193783334143	0.9032228165002245	0.0990344783783498
0.1960597710503188	0.3934872935569416	0.0969603190193335
0.6499460171633342	0.7649178430234461	0.2492373734881415
0.4428128402643539	0.4268222944248306	0.2462796164383542
0.5845611735332059	0.7294831033665814	0.3715169866163158
0.0292231643216762	0.3182510334321088	0.1490333750534302
0.0392658097913846	0.8311495607560929	0.1507762785890878
0.5332988854775430	0.3174369008457482	0.1474582626596208
0.5419820070890780	0.8361909310787798	0.1383224994008541
0.0257255420992356	0.0628339678187546	0.1346576383757449
0.0222641861492864	0.5510958085890422	0.1484234226065460
0.5390938327306557	0.0629409531463613	0.1311848010532044
0.5249875669317893	0.5651802802746877	0.1641341719127568
0.7730444083962243	0.2926804569795604	0.1455679465721879
0.7843625630081554	0.8265876150947410	0.1383637778338862
0.2788266315801848	0.3079297990883373	0.1447793043369164
0.2898248065087839	0.8221494908882543	0.1519521352477533
0.7800948722725590	0.0645804098836081	0.1272493104600503
0.7746992122466853	0.5705878804483632	0.1450967360825380
0.2939913381722645	0.0613535791788919	0.1350199203203426
0.2775304328562926	0.5686037642204795	0.1456934480822424
0.1064286836535311	0.2265185121741290	0.0499940981416856

0.1224780852099507	0.7356843328041888	0.0575120886958025
0.6197952841289727	0.2282490717787131	0.0500678896659869
0.6187620926682789	0.7337864620935719	0.0477123961946240
0.1209980061868710	0.9791317074693875	0.0405040317605306
0.1141573743954344	0.4862531564043537	0.0471048396206177
0.6249312953327422	0.0008233402404849	0.0345164056401102
0.6190716346244347	0.4941509823647677	0.0461168656724009
0.8635138654595274	0.2374209814780373	0.0488423294541309
0.8775881656579165	0.7441624072085102	0.0472031586726899
0.3714723819939369	0.2240628780737327	0.0469212635990323
0.3678096116724475	0.7454255717261986	0.0517001422397918
0.8663125626605180	0.9771328457227029	0.0335759820865946
0.8648925468509688	0.4795007498544211	0.0505494765599663
0.3635989400147907	0.9612344893353932	0.0410895735487487
0.3651237655653967	0.4822440311096368	0.0472285711282844
0.5105651278528958	0.5352301971990963	0.2232366515316410
0.5025029823519124	0.6295035760831544	0.3286420433061737
0.5868036656794026	0.6376257455181669	0.2734172244228338
0.6783119891754358	0.8189467019450701	0.2869706482806110
0.7723032417308205	0.5818637587097516	0.1903859469955216
0.2835533979960351	0.5738412803988469	0.1914473069041157
0.0312446773330204	0.5435577937719928	0.1935847670393792

0.4260245284929973	0.6452665426905339	0.3111540768803824
0.5329251037961306	0.7423668839597467	0.4023580904854080
0.4609871588956570	0.5356247959323031	0.3508723841640521
0.6586279043853218	0.6153574620636018	0.2918904574340470
0.1153248484134055	0.9667320688167513	-0.0050353857067154
0.1125623855511196	0.4844526230688042	0.0011541736651144
0.6355775204051509	0.0262851417151283	-0.0097562584982231
0.6063699610208063	0.4873166536477599	0.0005781612488171
0.8764148745983822	0.9813925833813948	-0.0120639047247211
0.8662663721113838	0.4804778805408856	0.0046025590750841
0.3452832970272334	0.9213989975044525	-0.0004770394893696
0.3671837992149389	0.4819490089836589	0.0012992618871286
0.0160381963996563	0.2992540035063095	0.1938893855423399
0.0462329917074160	0.8548697929431951	0.1950854857726736
0.5200466933124700	0.3160949447839760	0.1928710238871884
0.5650643050800603	0.8594843359850580	0.1824149192015352
0.7696809816486994	0.2622407224650298	0.1885088001222711
0.7560821154234144	0.8129102622449029	0.1827267674039445
0.3030243326504166	0.3123542590169413	0.1892724662161841
0.2906903022892725	0.8327435641644004	0.1974852825703048

Supplementary Dataset 11. The coordinates of the glycerate : lattice oxygen attack

step on nickel/cobalt surface. The energy of the glycerate : lattice oxygen attack step on nickel/cobalt surface was calculated to be -417.00 eV.

Ni Co O C H

1.00000000000000

11.8928003311000001 0.0000000000000000 0.0000000000000000

-5.9265986588999997 10.3212535711999998 0.0000000000000000

0.0000000000000000 0.0000000000000000 21.1420993804999995

Ni Co O C H

8 8 35 3 24

Direct

0.7493366438145560 0.5037514952238437 0.1077612238636024

0.7572210023332439 0.0125182103653501 0.0966666941161513

0.2527656421937367 0.5035702637336446 0.1025016251914484

0.2508283114585034 0.0064826629023255 0.0937319639442602

0.5063076543529287 0.7610140550022521 0.1059900590680193

0.5024124260522845 0.2561823978014650 0.0896165049711238

0.0035585327567177 0.7580457537949578 0.1063000693917586

0.0093530594149359 0.2551385364349904 0.0947474993583404

0.7693101221840541 0.7841779468898574 0.0903747159367308

0.7479650940124358 0.2486526336132979 0.0957967180297705

0.2590657937878517 0.7691434535756749 0.1039582026178802

0.2521995393948153 0.2557949123360858 0.0928035580852937

0.4955285469086285	0.4952000524001446	0.0922442431762206
0.5019629389136624	0.0071809301785860	0.0928474089006064
0.9941967983439908	0.4932417654936467	0.0957374153412495
0.0090598270197566	0.0081002559660109	0.0949217589258907
0.9133872859631393	0.8376695235408353	0.1528281550638973
0.9161083281045063	0.3212642725372442	0.1491166067286331
0.4158207185600158	0.8359580553901904	0.1583699552898971
0.4053124277091633	0.3242658456112441	0.1423704118753764
0.9226065895290577	0.5843780980638767	0.1548160538889556
0.9378396203740499	0.0973907986127391	0.1360438058270902
0.4230625981849352	0.5819035047137214	0.1503371696928900
0.4197695782918575	0.0802826921692222	0.1343384645092028
0.6782637677840255	0.8459112467443906	0.1493227585153531
0.6610545081593566	0.3290783654771406	0.1478961819664681
0.1713712639913423	0.8399600878379671	0.1564394144809660
0.1705898967997046	0.3207624995692597	0.1485076259253797
0.6633970602143807	0.5854502021256232	0.1708561207152433
0.6599174158514094	0.0958111913599996	0.1412808319128268
0.1683759297099261	0.5899838842745999	0.1515494461062019
0.1737120286695273	0.0975668827211746	0.1356474586499828
0.8486750155335765	0.9277816802394723	0.0447849856208184
0.8370462296178515	0.4215201081141469	0.0566313427973387

0.3307174259486063	0.9129517157192283	0.0522846876333547
0.3306521781577100	0.4177665491913051	0.0525444184060744
0.8333519545044790	0.6699942062041171	0.0573354818821300
0.8443872153954511	0.1804367503641425	0.0439141394810360
0.3367737906984565	0.6759526003616758	0.0570578276419199
0.3316109028051352	0.1819102636495222	0.0412210909383925
0.5724865306429562	0.9286150616792541	0.0537686062202213
0.5896710926149760	0.4400634472381357	0.0539577111934073
0.0858557632194781	0.9232249595004697	0.0557854074511271
0.0866765424445325	0.4203416524940477	0.0570197534774204
0.5882931089771236	0.6762479237749609	0.0524335020475786
0.5905642790997614	0.1792501176033642	0.0379222641006888
0.0871648140263962	0.6717189872567575	0.0545626753427343
0.1055700151407278	0.1961179591574115	0.0395547143542609
0.6771185733154145	0.3963539819407421	0.3582842799341758
0.5316524756077375	0.3807113208365273	0.2573688070682512
0.7350375887524563	0.7266797052750857	0.2528111801689162
0.6244416372021143	0.5064253285541933	0.2807274643008212
0.7360388884276899	0.4977140160053141	0.3115099989604760
0.6778389997919976	0.6142533609529854	0.2300584723922696
0.9345074504695585	0.5873334464104084	0.2001882195143072
0.4255389567262667	0.5750365605308505	0.1959118852514989

0.8808375780517790	0.8284492357811274	0.1960964144714469
0.9062677453927939	0.3026730247410912	0.1940666691691758
0.4353193485115627	0.8831396058375524	0.1979486458575832
0.4088011889715699	0.3230202665891996	0.1884680753165587
0.6979507597429249	0.8375471140845295	0.1933335708534148
0.6330053529376826	0.3085404593818166	0.1915281946407155
0.1938562633081076	0.8737779937678279	0.1990432716853463
0.1447511806860473	0.2667386745735559	0.1863162002367745
0.8238928190051333	0.6500841166419822	0.0125172735678350
0.8454695161374951	0.1795619154988389	-0.0019201548272772
0.3384039944066614	0.6826656593267991	0.0113720287181057
0.3208925558874193	0.1876311533678990	-0.0039489995715339
0.5849603148875044	0.6751835456538103	0.0065710577054826
0.5972847966699975	0.1755645596391763	-0.0076106629602347
0.0946980362510083	0.6832759262481072	0.0090473724410055
0.1322897426518096	0.2485800839314445	0.0012035498051291
0.7354110135397676	0.3633776879495108	0.3673542689366788
0.8046739366522916	0.5913076950880432	0.3331440265251945
0.7856196890907928	0.4716234735202181	0.2750682466755560
0.5790183557923578	0.5371630958480139	0.3172673030458672
0.5200644730879039	0.3212559699255991	0.2919872207897768
0.1701731661129132	0.5947483897121160	0.1973381704385442

Supplementary Dataset 12. The coordinates of the glycerate : lattice oxygen attack step on nickel surface. The energy of the glycerate : lattice oxygen attack step on nickel surface was calculated to be -392.15 eV.

Ni O C H

1.00000000000000

11.7038002014000000	0.0000000000000000	0.0000000000000000
-5.8513176253000001	10.2171014480999993	0.0000000000000000
0.0000000000000000	0.0000000000000000	21.1695003509999999

Ni	O	C	H
16	35	3	24

Direct

0.7412210857158406	0.7345640678468026	0.0996856590547069
0.7333657748834688	0.2240919040195802	0.0930385334214384
0.2387540453224728	0.7312751975686570	0.1001396236902177
0.2344875257603680	0.2244750863360748	0.0939905407175402
0.7360425628323574	0.9761749251606705	0.0925388837985661
0.7317858517946692	0.4691137609339200	0.1060071295733069
0.2362047236105119	0.9773324892200201	0.0948485022367905
0.2320652759439962	0.4693479042868062	0.1054896993923570
0.9896964864138641	0.7315152406871729	0.0993864455750402
0.9839643797675427	0.2248528164476907	0.0931813505162986

0.4908409352323035	0.7337365792679836	0.0995527701651765
0.4832533283440502	0.2240658150494507	0.0941355952930899
0.9881975202772142	0.9778023054475495	0.0941092339386603
0.9806801690407525	0.4673589729218912	0.1053863945240602
0.4844150783856509	0.9768789343039572	0.0944568349037955
0.4812266592723990	0.4673337911253514	0.1063781905400376
0.8298959611368536	0.9062188074415115	0.1472103579259983
0.8132910421128647	0.3847655153014571	0.1555715610675535
0.3301875842442051	0.9083986606167535	0.1508034769352788
0.3121760603747967	0.3848979318395668	0.1541426295325147
0.8218004895491754	0.6437473011205981	0.1500288153821418
0.8149895792962645	0.1371903797334310	0.1338333127649700
0.3164835237965429	0.6390526052759998	0.1501547997588981
0.3145432952125458	0.1366916933303552	0.1361014959978020
0.5799160901101968	0.9048273831911290	0.1470536312683613
0.5643452087082873	0.3831375325627504	0.1548108339036199
0.0819419956451965	0.9103036429739337	0.1508897433470102
0.0652795550209886	0.3850070917831121	0.1554302138996146
0.5674627685735042	0.6380798224409233	0.1702862188720610
0.5653064824551768	0.1380086743037914	0.1351965939030760
0.0702785933737007	0.6449830761213656	0.1493602080706101
0.0656396618511884	0.1386488563164772	0.1350266267017909

0.9123313636382169	0.8159387236138808	0.0541092907561123
0.9016885550002078	0.3095882305642758	0.0531283088740959
0.4008130877196604	0.8148889962618476	0.0545536158084698
0.4007401343757319	0.3077456474540670	0.0535855567967082
0.8999016629246549	0.5556124706799803	0.0550411491492412
0.8940280758192269	0.0477079436391768	0.0403821117670741
0.4041105025038500	0.5599283594660888	0.0573775913745527
0.3919775554123693	0.0474507282484680	0.0413623986267690
0.6567766404212936	0.8135417993011911	0.0530130435475335
0.6532197697898149	0.3147564566008944	0.0542103139237553
0.1562561291673849	0.8152706933048449	0.0554123737780139
0.1565223926459928	0.3180505488153736	0.0545327888814160
0.6523944581462585	0.5600881787154911	0.0581461855280227
0.6411313697256833	0.0467784231009985	0.0408522888531688
0.1517658541074182	0.5545190190462295	0.0553610312870338
0.1447206646199846	0.0493395316421740	0.0413143067572385
0.6288325961270077	0.4866119702946909	0.3625350184739460
0.4624385466955956	0.4371315955794299	0.2642542166402772
0.6428373375796881	0.7874486295374309	0.2494330953233842
0.5501746739493708	0.5702303881639881	0.2823190898788921
0.6739704120215754	0.5795785727020083	0.3113636249855117
0.5888765098122691	0.6727974102276332	0.2290983973475224

0.8328614251011964	0.6541304271620516	0.1954829858728393
0.3139123614709045	0.6403398679766831	0.1959772823919356
0.8915062759916441	0.5406434344593527	0.0098209497466048
0.8785720081081703	0.0220143053460844	-0.0037639889870397
0.4029210243593037	0.5501576404476006	0.0118694536824273
0.3754648441244758	0.0193047017197527	-0.0024375846539432
0.6457609809014574	0.5509793656766643	0.0125525594275730
0.6245238944398424	0.0217616887581366	-0.0033530868392179
0.1464650766929078	0.5380924509951612	0.0102467491003139
0.1285434058261821	0.0226174565452942	-0.0026843742360614
0.8282154312946091	0.9215065991079205	0.1922180745228394
0.7923179197099580	0.3538268906529973	0.1986991150807196
0.3577330452843400	0.9523395379494900	0.1913914694079584
0.3214162907527075	0.3833259984700843	0.1997857556955218
0.6018538259468457	0.9064376638897849	0.1916490655603622
0.5445929662987467	0.3795340069033259	0.1996636776702725
0.1068021423486194	0.9600467393059332	0.1900444421673222
0.0563925410848966	0.3563709086415323	0.1989324127378643
0.6965452836085001	0.4646192398620459	0.3725195287681577
0.7394404022484459	0.6799780895625356	0.3282051386041927
0.7247882765418907	0.5524476095781465	0.2754645027996024
0.5024986529598112	0.5994459395455163	0.3188723405296878

0.4602564049759281 0.3849749399898999 0.3007561569170979

0.0775169567635621 0.6596210563765357 0.1946747656670539

Supplementary Dataset 13. The coordinates of the glyceric acid formation step on cobalt surface. The energy of the glyceric acid formation step on cobalt surface was calculated to be -436.60 eV.

Co O C H

1.00000000000000

12.1820001601999994 0.0000000000000000 0.0000000000000000

-6.0910000800999997 10.5499216077000000 0.0000000000000000

0.0000000000000000 0.0000000000000000 21.1058006286999991

Co O C H

16 35 3 24

Direct

0.9376584155894806 0.1436926100251670 0.1010679773699593

0.9536528400019898 0.6591030893313286 0.0895901160612557

0.4531108119604449 0.1473021043531464 0.0824661683206558

0.4516864660703565 0.6558505776146106 0.1039391991705655

0.9475032257178243 0.9087978508505178 0.0919599157018404

0.9415833486272603 0.3895062577464100 0.0976164814941186

0.4463355528586658 0.9011638653164467 0.0915803685551082

0.4322408747201538 0.3681019916034966 0.0786254040346316

0.6943648343138791	0.1520679488481544	0.0910967504551238
0.7168446087924835	0.6600875056660056	0.0633129279623546
0.1984578167385564	0.1510158838623145	0.0905443710975382
0.2073634117842318	0.6584552619985974	0.0909487274720439
0.6934572083117808	0.8996099257052355	0.0983903686682112
0.6932067658237506	0.3978086556344367	0.0985664089548477
0.2036096444802187	0.9070524573582124	0.0872041385288228
0.1983983125990825	0.3845502579973074	0.0955181160395617
0.5576727032051125	0.6267811099430575	0.1808934281736761
0.5177309380824340	0.3963007783604466	0.2970533506494982
0.3804614265689499	0.6494923825257612	0.2693788470731782
0.0344357793694504	0.3134815080458217	0.1499827999918728
0.0406939217440573	0.8467822178506399	0.1401299576110769
0.5259586720280207	0.3421471260471571	0.1462854801708663
0.5238246378813558	0.8357466172303718	0.1465383336032062
0.0211297566183452	0.0589003806746360	0.1377608907898124
0.0383165841520030	0.5653625779413919	0.1434652548570458
0.5244068662349328	0.0624414655099940	0.1297751216047362
0.7034504403194850	0.5599722782786755	0.2617429684892008
0.7893418104023392	0.3312946283903299	0.1532125543720493
0.7747495369743086	0.8285604892111920	0.1623926898118770
0.2836303625190992	0.3030576218390853	0.1379083353006249

0.2970007496550173	0.8349447925946915	0.1442104581944320
0.7732685685326375	0.0768515047704727	0.1373183446019271
0.7753133778709219	0.5816876161055015	0.1314484856561520
0.2892510195541730	0.0690297582320425	0.1239894380158877
0.2760128254883258	0.5522968397117202	0.1461593820257817
0.1170345248829569	0.2341766902938856	0.0509244359465696
0.1257511001832292	0.7465581448718202	0.0529424361022642
0.6177591528308625	0.2334863198078178	0.0505286120599766
0.6135707049788910	0.7221486525785910	0.0569745828158797
0.1178160340526910	0.9981661910115067	0.0375001407040021
0.1158441948968045	0.4812430201018092	0.0487679544083515
0.5962777210022283	0.9654622737797529	0.0417357734956400
0.6104771463870561	0.4791683112125464	0.0367551448198135
0.8641417542492158	0.2431302135326714	0.0519287693740174
0.8825576443684363	0.7610003251761078	0.0422349477743101
0.3797052978398788	0.2291307378556378	0.0329088016330190
0.3624581897014688	0.7348555239882637	0.0536736328806499
0.8611162533986515	0.9793116370295188	0.0455808087188159
0.8661841399041071	0.4798792632443726	0.0485559073988122
0.3636258877392760	0.9611530531405423	0.0366659237215765
0.3707957567475579	0.4847442958337886	0.0501342044375205
0.5879539788523085	0.5061314875135539	0.2643235581002127

0.4798505103798743	0.6258704698304645	0.2890277928607863
0.5046782307906826	0.5544850614051422	0.2359975086070673
0.6481055939695919	0.7074408780766247	0.1837579009559303
0.7663364013069175	0.6020006662928512	0.1751912839013815
0.2783348524655613	0.5610006543286464	0.1921172276364185
0.0353120698770134	0.5654111319006149	0.1893671903755603
0.4518781476094051	0.5676076831284209	0.3323870439091021
0.3549244984127040	0.6805393751088852	0.3056493274048479
0.5680847330511943	0.7160143181560544	0.2982202184714644
0.4140337674083561	0.4711176860597158	0.2220083392020594
0.1269005590404577	0.0144297537888894	-0.0077848847801031
0.1076369870797074	0.4750617890772952	0.0029610811074835
0.5803178621597797	0.9450186156493905	-0.0030000264641736
0.6033105877069963	0.4564477863833490	-0.0077664561999291
0.8649841256246609	0.9737019916083722	-0.0001983089841656
0.8622548930466999	0.4653350086008493	0.0032265319888195
0.3446092546581729	0.9201321809858172	-0.0046231174435017
0.3825622677654651	0.5100311002806623	0.0058963254907632
0.0387867808528497	0.3049424462500521	0.1954729093181283
0.0586575103164461	0.8871765149440827	0.1816171233060677
0.4707024426482280	0.2867776042536030	0.1795447482587506
0.5243742860976024	0.8564808389823055	0.1909536249377974

0.7801539980569838 0.2948777153763288 0.1950198822553948

0.8182689941380012 0.8919845284112149 0.1952459936449444

0.5778298750808608 0.3726322043758454 0.3141824696099835

0.3178369743314213 0.8692961716015734 0.1868689601255430

Supplementary Dataset 14. The coordinates of the glyceric acid formation step on nickel/cobalt surface. The energy of the glyceric acid formation step on nickel/cobalt surface was calculated to be -416.00 eV.

Ni Co O C H

1.00000000000000

11.8928003311000001 0.0000000000000000 0.0000000000000000

-5.9265986588999997 10.3212535711999998 0.0000000000000000

0.0000000000000000 0.0000000000000000 21.1420993804999995

Ni Co O C H

8 8 35 3 24

Direct

0.7492334837352497 0.4975315613868329 0.1161488215059044

0.7546378078693771 0.0097728548191172 0.0958912668247531

0.2562094592948467 0.5075149207254960 0.0915360015025496

0.2516071089166732 0.0064969583609793 0.0976765081868694

0.5120037983610368 0.7625914274894303 0.0955236748274339

0.5017980494316013 0.2557199643658085 0.1009966326457283

0.0005840891945500	0.7599220820600079	0.1169789456872949
0.0055980832718967	0.2572672039625807	0.0927031374261102
0.7524622390847102	0.7798754120413028	0.1033684005773860
0.7523701067845616	0.2534111260702439	0.0988697873690234
0.2600048066784597	0.7701133701115469	0.1018450436978636
0.2558746501291279	0.2564175862360778	0.0904444715676286
0.4921665570703114	0.5025770368925409	0.0589791287361497
0.5048976669014457	0.0073927606215127	0.0961384765075479
0.0002757392327894	0.4958606336917670	0.0989506684885618
0.0048251893950942	0.0117869208013405	0.0939874424040247
0.9136914420814312	0.8482960308987522	0.1607300657856358
0.9201544929553591	0.3268997320106357	0.1530967675002266
0.4225844461014862	0.8341294710104660	0.1520270684285421
0.4202806567054140	0.3264435169810979	0.1633056326332176
0.9242469493873234	0.5829030946227431	0.1616767646835561
0.9241338877679721	0.0829695775961860	0.1379918462087771
0.4355167611310707	0.5813845014217619	0.1290130586751023
0.4164485999094455	0.0819633242057411	0.1389884070165097
0.6671393363532094	0.8264108573668777	0.1546549767415077
0.6652181441054321	0.3166371524520088	0.1549170542296176
0.1764918413744859	0.8453743936884568	0.1588654213177308
0.1791948494265132	0.3342867820651728	0.1409542436287233

0.6722972957333505	0.0933278522924978	0.1396885503185770
0.1788562551870149	0.5962209726255501	0.1488148060392017
0.1758716128768743	0.1031315286331532	0.1380230590518806
0.8407176403416983	0.9294308904280408	0.0502588448521192
0.8330197457812394	0.4170095633766956	0.0620131999162611
0.3303834461189251	0.9177469435445942	0.0540310919677511
0.3245832843157020	0.4057927253504959	0.0376590844152590
0.8242668369244222	0.6717620419155461	0.0722742518911033
0.8363165273861096	0.1829889508886451	0.0452303377979864
0.3386863202810831	0.6835283003388279	0.0475126929947008
0.3385983400011676	0.1814621411348608	0.0445148218264571
0.5913476044664001	0.9340786488256004	0.0540442748973055
0.5909995534044611	0.4312363141316541	0.0617018598953696
0.0795026789430867	0.9257511895896648	0.0601319830961397
0.0828640974435072	0.4355007242062230	0.0516322160670172
0.6005611830235547	0.6874876117816504	0.0363726132585550
0.5883217419093522	0.1835747357064712	0.0452066602190073
0.0799207291725885	0.6784290467653790	0.0597754197465128
0.0877782590326768	0.1756914420531889	0.0379507453198146
0.7898067874315049	0.4804615869303971	0.2830464133820291
0.6813874323438539	0.7668426504630985	0.3031603959483405
0.4919401902176990	0.6153743758535720	0.2592553520133219

0.6385525525215925	0.5343601753385041	0.1861544153320867
0.6870616023503323	0.5987677682209902	0.2442417203406178
0.6913311251819455	0.5133574780753434	0.2973332222071702
0.6082049504687679	0.6599202466462295	0.2677482379155857
0.9185561621663425	0.5661502730847664	0.2069006747613298
0.4472701394887439	0.5688221241945218	0.1736673343900753
0.9165162890613551	0.3098059655607641	0.1980407674410021
0.4376186086765920	0.8482706490899237	0.1970605545184266
0.3750770241209593	0.2553925725081332	0.1931881121898324
0.9150476534600864	0.8953651635041663	0.1984631605672058
0.6614437132156951	0.2915188014569459	0.1988086558206741
0.2052202324194783	0.8754990717495512	0.2015411600851653
0.1821198369001999	0.3160317532052103	0.1854658694001717
0.8173155531125631	0.6582081328586852	0.0268437712916076
0.8337783190415677	0.1950629354311358	0.0000224016877712
0.3335990943795973	0.6928783104841593	0.0022236971887431
0.3470462106003425	0.2008197935015720	-0.0004501003837425
0.6265860304046607	0.7259106301089604	-0.0054003146303578
0.5970316764361556	0.1990842456738883	-0.0000222477863928
0.0772919807302214	0.6876716183080053	0.0143030260742598
0.0838950657661564	0.1741910326522026	-0.0078444381584996
0.1865000052184686	0.5978607332996977	0.1944891364998226

0.7137346130750941 0.5677959265973573 0.3421738523549131

0.5954098431286282 0.4250971086083271 0.3013838946734310

0.8024010844826689 0.4400968876989183 0.3202553084603630

0.6266288379630413 0.8042925360157166 0.3134444580035123

0.7863661483199651 0.6777573996832637 0.2348838023785657

0.5472216591366611 0.4503905891760543 0.1864250614418453

Supplementary Dataset 15. The coordinates of the glyceric acid formation step on nickel surface. The energy of the glyceric acid formation step on nickel surface was calculated to be -391.34 eV.

Ni O C H

1.00000000000000

11.7038002014000000 0.0000000000000000 0.0000000000000000

-5.8513176253000001 10.2171014480999993 0.0000000000000000

0.0000000000000000 0.0000000000000000 21.1695003509999999

Ni O C H

16 35 3 24

Direct

0.7442929670160116 0.7389315718489572 0.0907595731922768

0.7358851512197386 0.2236779132017965 0.0944530683746500

0.2365490868210219 0.7300038378298235 0.1045160558059597

0.2291645566916526 0.2155650780650784 0.0880409112307883

0.7347845560589324	0.9718629238470428	0.0914463169256266
0.7325159400187773	0.4708786124812810	0.1015828732583758
0.2371729295986597	0.9756544032157863	0.0964896927410127
0.2317550238160707	0.4668466363068314	0.1091344047311467
0.9882334981890339	0.7300017964920936	0.0978283621792016
0.9835169923320009	0.2192383148072979	0.0921870769044729
0.4852345488940960	0.7317057530280798	0.1089084473053995
0.4835521707515859	0.2233200351774892	0.0935399894332812
0.9829945210006792	0.9709685483856866	0.0875921696928519
0.9816269160969924	0.4642244615332189	0.1071454229158298
0.4847110475452436	0.9766160234283744	0.0990424643415157
0.4808482269360205	0.4661276296044439	0.0976769640754054
0.8208050725311324	0.8919821900203623	0.1370374834401921
0.8126134469470255	0.3861182159694200	0.1563467443053711
0.3280868411349168	0.9109107450800973	0.1548895554558318
0.3465539663694244	0.4249448499407121	0.1711783150730451
0.8112156081919676	0.6408554617948912	0.1436123114658766
0.8157131344514142	0.1319300526145831	0.1341176861971794
0.3095007918431415	0.6445009436633985	0.1544987899524944
0.3143631167277689	0.1394359620256941	0.1354247068416164
0.5781009748931398	0.9109020376341626	0.1548541989837500
0.5743542161489802	0.3965529283428137	0.1499117302723314

0.0768290212331574	0.9047620680311178	0.1477761041197181
0.0635429776658180	0.3778812123694918	0.1567507641314403
0.5682594915540080	0.1405530663914287	0.1371535219580637
0.0672927086238574	0.6423969067507027	0.1504154033691499
0.0637425976264153	0.1306179108620494	0.1317791458495071
0.9119108197686719	0.8114256511286210	0.0463629900729404
0.9019278325934861	0.3078301591749301	0.0541831787342807
0.3975835176529463	0.8138180148228635	0.0607977463413997
0.3988739407574984	0.3032057609927822	0.0510310999632158
0.8943981461804722	0.5474182499605226	0.0550241621941719
0.8968077362841997	0.0512427552061810	0.0349006017437838
0.4020789234716275	0.5624978426385570	0.0606656780075239
0.3954527672770264	0.0464339900541049	0.0443267845571725
0.6460530503106640	0.8053885297276872	0.0555326849810278
0.6566121924839080	0.3171339003242347	0.0542318189929418
0.1636502662099421	0.8133804595056044	0.0571794900411607
0.1545302295506785	0.3099602825769325	0.0541320685427398
0.6536369122960300	0.5704204108676734	0.0504692887476333
0.6422179321057885	0.0431201924643095	0.0436753011560929
0.1499505994069842	0.5546694314970092	0.0573735172913605
0.1453201353674604	0.0430675070807040	0.0391748840650568
0.7033343145143103	0.5755765747413130	0.2659218784398651

0.6099359193480820	0.8777213595898583	0.3075447721354818
0.4082857391225827	0.7178859620374202	0.2770431431823491
0.5414514952013990	0.6398746713495893	0.1871560589316937
0.6050772417124555	0.7103395921026102	0.2420112832892732
0.6223337106130092	0.6248410836081199	0.2915467709628461
0.5280832350853593	0.7669529663905958	0.2751125009841786
0.7968347889679237	0.6384227106591089	0.1892322396475760
0.3157745799881723	0.6572832176998892	0.2002612899432900
0.8824056599039952	0.5230888651710461	0.0106324022982416
0.8928996257254842	0.0459354230307624	-0.0108055051685205
0.3999176676760831	0.5690701063832178	0.0150968682659083
0.3790458577632986	0.0139711115255019	0.0012018439947101
0.6502036825708687	0.5732091857685715	0.0046796968306172
0.6275336261545192	0.0170721202983396	-0.0004634674139716
0.1445852106957093	0.5436000689577642	0.0119100650702125
0.1347968726269961	0.0233969147238373	-0.0056719580724567
0.7929591129606499	0.3539635426025203	0.1992983381848963
0.3494808905918901	0.9546886664788781	0.1955754429122848
0.3194025324879147	0.3416888745894659	0.1920133806941343
0.6217737227349865	0.9705811426996415	0.1897857108626897
0.5907482526234830	0.4151770412720322	0.1948032787715816
0.0842141242619507	0.9480799838359730	0.1878117891535757

0.0539297102415469 0.3550883995177083 0.2011252825331808

0.0752621308527281 0.6558905326254304 0.1958128664943347

0.6685690427056239 0.6844279078410088 0.3339362152252887

0.5247174275885216 0.5422910473188302 0.3042784359641056

0.7415819781037075 0.5517188537813713 0.3006186908955696

0.5534209604473198 0.9054773965832932 0.3288153238426400

0.7018427364677082 0.7920864258471266 0.2274154961156163

0.4541881611286488 0.5472185934565753 0.1937050680319817

Supplementary Dataset 16. The coordinates of the glyceric acid desorption step on cobalt surface. The energy of the glyceric acid desorption step on cobalt surface was calculated to be -435.39 eV [-359.19 eV – 76.20 eV (glyceric acid)].

Co O C H

1.00000000000000

12.1820001601999994 0.0000000000000000 0.0000000000000000

-6.0910000800999997 10.5499216077000000 0.0000000000000000

0.0000000000000000 0.0000000000000000 21.1058006286999991

Co O C H

16 35 3 24

Direct

0.9376584155894806 0.1436926100251670 0.1010679773699593

0.9536528400019898 0.6591030893313286 0.0895901160612557

0.4531108119604449	0.1473021043531464	0.0824661683206558
0.4516864660703565	0.6558505776146106	0.1039391991705655
0.9475032257178243	0.9087978508505178	0.0919599157018404
0.9415833486272603	0.3895062577464100	0.0976164814941186
0.4463355528586658	0.9011638653164467	0.0915803685551082
0.4322408747201538	0.3681019916034966	0.0786254040346316
0.6943648343138791	0.1520679488481544	0.0910967504551238
0.7168446087924835	0.6600875056660056	0.0633129279623546
0.1984578167385564	0.1510158838623145	0.0905443710975382
0.2073634117842318	0.6584552619985974	0.0909487274720439
0.6934572083117808	0.8996099257052355	0.0983903686682112
0.6932067658237506	0.3978086556344367	0.0985664089548477
0.2036096444802187	0.9070524573582124	0.0872041385288228
0.1983983125990825	0.3845502579973074	0.0955181160395617
0.5576727032051125	0.6267811099430575	0.1808934281736761
0.5177309380824340	0.3963007783604466	0.2970533506494982
0.3804614265689499	0.6494923825257612	0.2693788470731782
0.0344357793694504	0.3134815080458217	0.1499827999918728
0.0406939217440573	0.8467822178506399	0.1401299576110769
0.5259586720280207	0.3421471260471571	0.1462854801708663
0.5238246378813558	0.8357466172303718	0.1465383336032062
0.0211297566183452	0.0589003806746360	0.1377608907898124

0.0383165841520030	0.5653625779413919	0.1434652548570458
0.5244068662349328	0.0624414655099940	0.1297751216047362
0.7034504403194850	0.5599722782786755	0.2617429684892008
0.7893418104023392	0.3312946283903299	0.1532125543720493
0.7747495369743086	0.8285604892111920	0.1623926898118770
0.2836303625190992	0.3030576218390853	0.1379083353006249
0.2970007496550173	0.8349447925946915	0.1442104581944320
0.7732685685326375	0.0768515047704727	0.1373183446019271
0.7753133778709219	0.5816876161055015	0.1314484856561520
0.2892510195541730	0.0690297582320425	0.1239894380158877
0.2760128254883258	0.5522968397117202	0.1461593820257817
0.1170345248829569	0.2341766902938856	0.0509244359465696
0.1257511001832292	0.7465581448718202	0.0529424361022642
0.6177591528308625	0.2334863198078178	0.0505286120599766
0.6135707049788910	0.7221486525785910	0.0569745828158797
0.1178160340526910	0.9981661910115067	0.0375001407040021
0.1158441948968045	0.4812430201018092	0.0487679544083515
0.5962777210022283	0.9654622737797529	0.0417357734956400
0.6104771463870561	0.4791683112125464	0.0367551448198135
0.8641417542492158	0.2431302135326714	0.0519287693740174
0.8825576443684363	0.7610003251761078	0.0422349477743101
0.3797052978398788	0.2291307378556378	0.0329088016330190

0.3624581897014688	0.7348555239882637	0.0536736328806499
0.8611162533986515	0.9793116370295188	0.0455808087188159
0.8661841399041071	0.4798792632443726	0.0485559073988122
0.3636258877392760	0.9611530531405423	0.0366659237215765
0.3707957567475579	0.4847442958337886	0.0501342044375205
0.5879539788523085	0.5061314875135539	0.2643235581002127
0.4798505103798743	0.6258704698304645	0.2890277928607863
0.5046782307906826	0.5544850614051422	0.2359975086070673
0.6481055939695919	0.7074408780766247	0.1837579009559303
0.7663364013069175	0.6020006662928512	0.1751912839013815
0.2783348524655613	0.5610006543286464	0.1921172276364185
0.0353120698770134	0.5654111319006149	0.1893671903755603
0.4518781476094051	0.5676076831284209	0.3323870439091021
0.3549244984127040	0.6805393751088852	0.3056493274048479
0.5680847330511943	0.7160143181560544	0.2982202184714644
0.4140337674083561	0.4711176860597158	0.2220083392020594
0.1269005590404577	0.0144297537888894	-0.0077848847801031
0.1076369870797074	0.4750617890772952	0.0029610811074835
0.5803178621597797	0.9450186156493905	-0.0030000264641736
0.6033105877069963	0.4564477863833490	-0.0077664561999291
0.8649841256246609	0.9737019916083722	-0.0001983089841656
0.8622548930466999	0.4653350086008493	0.0032265319888195

0.3446092546581729	0.9201321809858172	-0.0046231174435017
0.3825622677654651	0.5100311002806623	0.0058963254907632
0.0387867808528497	0.3049424462500521	0.1954729093181283
0.0586575103164461	0.8871765149440827	0.1816171233060677
0.4707024426482280	0.2867776042536030	0.1795447482587506
0.5243742860976024	0.8564808389823055	0.1909536249377974
0.7801539980569838	0.2948777153763288	0.1950198822553948
0.8182689941380012	0.8919845284112149	0.1952459936449444
0.5778298750808608	0.3726322043758454	0.3141824696099835
0.3178369743314213	0.8692961716015734	0.1868689601255430

Supplementary Dataset 17. The coordinates of the glyceric acid desorption step on nickel/cobalt surface. The energy of the glyceric acid desorption step on nickel/cobalt surface was calculated to be -414.15 eV [-337.95 eV – 76.20 eV (glyceric acid)].

Ni Co O H

1.00000000000000

11.8928003311000001	0.0000000000000000	0.0000000000000000
-5.9265986588999997	10.3212535711999998	0.0000000000000000
0.0000000000000000	0.0000000000000000	21.1420993804999995

Ni	Co	O	H
8	8	31	18

Direct

0.7526223040810277	0.4977957620113027	0.1061382523469177
0.7538062958493230	0.0064713516853467	0.0938401544684165
0.2553766228792348	0.5026651106719110	0.0981411326680750
0.2499727028469614	0.0034968580081886	0.0974901514136942
0.5127626351194194	0.7650047910443083	0.0991832876503786
0.4979240609401275	0.2605313742503529	0.0999240353951324
-0.0009048768903073	0.7574109978606428	0.1154196046813301
0.0023952917489254	0.2528993716146425	0.0937880780938600
0.7599352082190857	0.7727836884645595	0.0977635516045018
0.7469868939578534	0.2547140332077324	0.1014163294237402
0.2584152557635484	0.7648098225954882	0.1085552712239803
0.2513334752952080	0.2524085006626194	0.0908376480760732
0.4879811573821246	0.5043705900931620	0.0579878100929155
0.5057168680534796	0.0108775150544405	0.1000726859675371
0.0005302013959944	0.4927680532690926	0.0991414348772647
0.0048207700230564	0.0098445789237821	0.0833509334235011
0.8741761153168884	0.7971207419969463	0.1657602188441397
0.9063332335907991	0.3174908547814002	0.1513018807441582
0.4279011955524690	0.8385454908542387	0.1567826437928089
0.3918150656990468	0.3127389765119641	0.1552413147627829
0.9195454477324433	0.5803957350545316	0.1577564744153261
0.9203783480880853	0.0687729360518926	0.1336619551276441

0.4455955801326563	0.5808999370739109	0.1346492739537701
0.4163673954590200	0.0798255173992642	0.1399829759377630
0.6870559885513100	0.8514066472249374	0.1405699246505527
0.6577296221699387	0.3353682662702139	0.1567476283156442
0.1712684793797567	0.8428073344409713	0.1615979311928895
0.1672431178526047	0.3302020473702482	0.1451841267254977
0.6619484172258279	0.0991053506564704	0.1448480497074069
0.1810422815291520	0.5971132577790976	0.1552176127253750
0.1656741796374460	0.0947687472935330	0.1344492834305718
0.8514234363275185	0.9207345230935026	0.0399704205519907
0.8315728645110605	0.4099323586268622	0.0591691904053575
0.3332178645696567	0.9134250838392062	0.0591310075879105
0.3198482694531334	0.4051285957576924	0.0407893849679011
0.8298093462485956	0.6722327366788318	0.0625259068648834
0.8299179820160881	0.1660121260174802	0.0468825815333774
0.3386256364366393	0.6787396028898188	0.0550519749342730
0.3361159759643343	0.1718839476039137	0.0420832375019184
0.5867887260497328	0.9417180614987112	0.0544868692682636
0.5906174615160856	0.4383990866657587	0.0589982244726794
0.0806867690100189	0.9186207838851839	0.0587268006912054
0.0871336362194907	0.4331977445295682	0.0553277655600395
0.6001179743535775	0.6914968946797416	0.0373132186476595

0.5935989553774659	0.1885968485338643	0.0485832666388167
0.0848398883938302	0.6771238789345045	0.0614721715756137
0.0921728962917560	0.1797806440334475	0.0365587357913250
0.9148443511661548	0.5750668672002117	0.2034620549040683
0.4519530031423182	0.5396285045710005	0.1731092962455914
0.8934527217274379	0.2922599760580981	0.1955032960068518
0.4473866226302783	0.8616130595849760	0.2009997194981854
0.3528710138341778	0.2444035157951119	0.1870303221354672
0.8864292809950048	0.8737426782542910	0.1886565698313117
0.6490871746896696	0.3182899235105424	0.2017444380344691
0.1945380559490898	0.8736214871616708	0.2046359230920824
0.1693947097110348	0.3091926513393605	0.1892917217486596
0.8299218620442813	0.6710932127218553	0.0165956631511090
0.8177664094182037	0.1545687218903895	0.0014792009292361
0.3309169244260687	0.6845848671386989	0.0096932510641212
0.3432293399230372	0.1809922090122585	-0.0035267276060823
0.6285305397875007	0.7288537444026715	-0.0045410070980948
0.6127642648091790	0.2064137764164224	0.0038776143421495
0.0892377313399059	0.6890419475287006	0.0159733163554126
0.0997471715947383	0.1968287178715852	-0.0085647574368634
0.1870622532275585	0.5985014175670856	0.2009692705667378

Supplementary Dataset 18. The coordinates of the glyceric acid desorption step on nickel surface. The energy of the glyceric acid desorption step on nickel surface was calculated to be -390.14 eV [-313.94 eV – 76.20 eV (glyceric acid)].

Ni O H

1.00000000000000

11.7038002014000000 0.0000000000000000 0.0000000000000000

-5.8513176253000001 10.2171014480999993 0.0000000000000000

0.0000000000000000 0.0000000000000000 21.1695003509999999

Ni O H

16 31 18

Direct

0.7492635910660338 0.7434389619859695 0.0925603717493463

0.7332100449951231 0.2232420574476980 0.0968413392303049

0.2384428152909984 0.7323382951885613 0.1015123840185589

0.2328863840120638 0.2231818844788716 0.0935724929156734

0.7364698704320159 0.9740885096558555 0.0914210801601066

0.7336926064127949 0.4727966336371422 0.1052769816012976

0.2371763131371072 0.9790319912056418 0.0977279190002886

0.2313739603270859 0.4681136269786842 0.1058318210148341

0.9902177166414831 0.7316961921642945 0.0985497653389933

0.9821282408688945 0.2207548982584238 0.0942282946486996

0.4841830508039305 0.7354248684557148 0.0944445612227005

0.4838861025470371	0.2262102079114852	0.0966504189387802
0.9850648107808991	0.9744108389334736	0.0904300442156913
0.9815233457246895	0.4666701372822738	0.1057875670222544
0.4834449472720004	0.9787503564319356	0.0968080247617437
0.4800140070941549	0.4672911847326346	0.1020931627054065
0.8252376737550855	0.8960875723756161	0.1378339911384750
0.8161625632844417	0.3885567198002813	0.1575574269624915
0.3304658568981435	0.9097715205836724	0.1542519321656804
0.3325170687448007	0.4088551130278803	0.1556327267002333
0.8170170838264120	0.6518428127825926	0.1474552610054524
0.8141477633421403	0.1318762212331902	0.1356761480082435
0.3188088046042776	0.6436450076209340	0.1490900124469175
0.3145891537325035	0.1412406663823454	0.1386328407809352
0.5758175541595414	0.9018276802704500	0.1478257282355328
0.5698100766724417	0.3973633082656469	0.1552313852190411
0.0801786480584415	0.9055622104052199	0.1496981084434499
0.0660286688620170	0.3837450014582186	0.1560219364339253
0.5655140688659143	0.1389754956850280	0.1389427515667612
0.0704980484287139	0.6422591933971181	0.1494512589225358
0.0626393088872242	0.1343327631814748	0.1352687849360723
0.9173654576986880	0.8149147998401486	0.0477401612534534
0.9007143654785102	0.3089107864141261	0.0556623093269306

0.3952672438662930	0.8199262794478005	0.0543291139958016
0.4003414966505828	0.3067116207297943	0.0534020200453825
0.8997459292437625	0.5535039441660794	0.0550731854080453
0.8967249866439354	0.0543650533325380	0.0363887749418327
0.4015360262673037	0.5594510832315942	0.0551674298061401
0.3930544993658440	0.0520753899417512	0.0453370690099428
0.6546481559025839	0.8096024115599005	0.0522730810051108
0.6528507350179177	0.3114696561535225	0.0562743734482576
0.1616493957455461	0.8169044386244554	0.0571085341426972
0.1530919278654508	0.3119317117027057	0.0552637451762500
0.6553572160562912	0.5738584643243219	0.0550712391417196
0.6419123578411364	0.0470954883075180	0.0434305524478951
0.1511706524993852	0.5560828637715225	0.0551203078207012
0.1441841649794360	0.0494108776777877	0.0423825668696539
0.8178304072655371	0.6659107436336751	0.1926047300492250
0.3250393119237830	0.6520168676177186	0.1947903104434265
0.8871180972119329	0.5347815554452319	0.0102718448111049
0.8822845405466311	0.0423042059689594	0.9911177771437977
0.4009761469752534	0.5500081186497116	0.0095814960231069
0.3769571950372835	0.0283347302979035	0.0009651779386847
0.6505391084181088	0.5783818197265632	0.0093458561164219
0.6302078295969969	0.0325030896123994	0.9981474370970917

0.1477370888561364	0.5447917844934924	0.0096128705676468
0.1296801807228359	0.0305863583084999	0.9975184351769623
0.8095586907904601	0.3696657217152721	0.2023691423098889
0.3561527583626152	0.9518995358784484	0.1953312112944642
0.3268077627134563	0.4138689019308696	0.2006787602578321
0.5988481062747643	0.9304538089471117	0.1912242418328288
0.5669940324735900	0.3868778314065530	0.2005181296328834
0.0922374968884480	0.9438879205995659	0.1914580847843759
0.0639361837884424	0.3686580723534306	0.2011727026844772
0.0766878364214146	0.6516992326830503	0.1951198492273848

Supplementary Dataset 19. The coordinates of the glycerate : configuration changed step on cobalt surface. The energy of the glycerate : configuration changed step on cobalt surface was calculated to be -437.10 eV.

Co O C H

1.00000000000000

12.1820001601999994	0.0000000000000000	0.0000000000000000
-6.0910000800999997	10.5499216077000000	0.0000000000000000
0.0000000000000000	0.0000000000000000	21.1058006286999991

Co	O	C	H
16	35	3	24

Direct

0.7554016809558844	0.7689307647921776	0.0833379494486163
0.7659209555979908	0.2696846951551595	0.0902027742054478
0.2680083394074504	0.7611577094436234	0.0847586462553906
0.2608258545239993	0.2615830849363219	0.0899585194929657
0.7518822294663506	0.5077863097687518	0.0917769988982763
0.7563571489436856	0.0129165938214316	0.0967187966236413
0.2698170621550803	0.5114530918063175	0.0875692719897975
0.2543764969183449	0.0048712861828576	0.0964713289110933
0.5110665730468642	0.7579790852798771	0.0876280819694701
0.5233143217852900	0.2731537070113839	0.0843605227803338
0.0248168036820467	0.7699473494006004	0.0812246733655496
0.0243432186505174	0.2766495310703523	0.0903006090905417
0.5152135975239289	0.5087925643445331	0.0863332034841368
0.5051378364460503	0.0062311295312691	0.0957998553030452
0.0165608197024967	0.5179770944927097	0.0894818794987212
0.0027873259747797	0.0092925812940054	0.0950946639742103
0.8364894896829806	0.9261453399839686	0.1465722345229677
0.8559842834262046	0.4450826096416035	0.1424851387307776
0.3320751612324169	0.9159186802862378	0.1450944437308362
0.3687634753305735	0.4408976294371312	0.1395317527305175
0.8352487693901557	0.6821484802335258	0.1348317265178213

0.8459415002906732	0.1885844313996586	0.1451885917799858
0.3521558434255629	0.6658556308348690	0.1280162561057954
0.3475354143320624	0.1808619289983087	0.1622123040573017
0.5895743622848311	0.9260263396563095	0.1475417660676501
0.6039748478465955	0.4458398358349261	0.1363466014071094
0.0847176333221717	0.9183863745201148	0.1440754270237829
0.1160959315539052	0.4471586931027233	0.1418246813578597
0.5940519655174858	0.6724422283232436	0.1245293523621523
0.5939592702130526	0.1860297325418213	0.1402156125896272
0.0952237558112888	0.6730581927268101	0.1293002734475886
0.0909524686950661	0.1802675813888637	0.1418927108725949
0.9076525427215404	0.8386673274004565	0.0467068623756009
0.9369727426827048	0.3552981991623996	0.0524615932101731
0.4284782237756665	0.8384909125204414	0.0466933743601604
0.4339764298585558	0.3426969459514232	0.0501320457595227
0.9169635437630197	0.5959907430280440	0.0384686843737400
0.9307078271812783	0.1098949876673518	0.0449760932338553
0.4226155173320970	0.5653216455766218	0.0347026337108450
0.4218409753053050	0.0970503105502250	0.0467426318469570
0.6732029195154660	0.8458041912277900	0.0463323334632753
0.6885461910098384	0.3420613756586223	0.0431710890679269
0.1746074247590387	0.8423662228325346	0.0395778974993886

0.1827881569396260	0.3546369366718500	0.0473605299283828
0.6649003025195680	0.5827976149636463	0.0334156788487528
0.6794677399515984	0.1019457099205581	0.0454943093007876
0.1737165457041213	0.5872103422797382	0.0372522762597087
0.1729755726117519	0.0887984815964110	0.0457029251866442
0.5885381709560340	0.4001016495982717	0.3288259177647500
0.2212257106755602	0.1792654029930088	0.2419061621876884
0.5142828821396893	0.1851688154809893	0.2608958079964303
0.4252568852040245	0.2280296088082144	0.2720804698073728
0.4968346266345111	0.3720900424816920	0.2788667243682394
0.3226732683632098	0.1905986873213123	0.2209895494159437
0.5749731880439506	0.1918086737650812	0.1851868277919158
0.1218946046616464	0.1828325897116531	0.1856983540755528
0.9069387927136759	0.5889871270847508	-0.0072331113286581
0.9289237724350908	0.1093423786592763	-0.0009472426602059
0.3977358647341119	0.5199355658679976	-0.0054769826822032
0.4144116529112167	0.0879924545300504	0.0010230002693785
0.6491409058738387	0.5599780572120598	-0.0111139373373457
0.6832047059128593	0.1005913010486727	-0.0003871788464989
0.1614288333252956	0.5779190205725288	-0.0083314771081907
0.1744920489817855	0.0820955919409902	-0.0000397859960793
0.8300095781127268	0.9139368044294491	0.1920648608178873

0.8702870746984818	0.4646638168531002	0.1873332240130163
0.3301670341433872	0.9026718134557229	0.1904570756565283
0.3895514091505491	0.4721771011708130	0.1827221217231556
0.5803042107719117	0.9152289846905455	0.1930358123892631
0.8129731668066974	0.6753378409819584	0.1794416151655611
0.0813744601145513	0.9016324038941709	0.1890984811785405
0.1333108955042692	0.4662595251904513	0.1865931376826739
0.4294931236043068	0.4037213718646355	0.2921291264463828
0.6542938393480002	0.4883557986853619	0.3249134550640873
0.5441725359131020	0.4155601913519243	0.2336255665214360
0.3745251070111629	0.1856642258901777	0.3166392261028397
0.8445683994346934	0.1906120832975140	0.1910724959291328
0.5849297090527071	0.2378756669179732	0.2893007165701333

Supplementary Dataset 20. The coordinates of the glycerate : configuration changed step on nickel/cobalt surface. The energy of the glycerate : configuration changed step on nickel/cobalt surface was calculated to be -417.44 eV.

Ni Co O C H

1.00000000000000

11.8928003311000001	0.0000000000000000	0.0000000000000000
-5.9265986588999997	10.3212535711999998	0.0000000000000000
0.0000000000000000	0.0000000000000000	21.1420993804999995

Ni Co O C H

8 8 35 3 24

Direct

0.7472203902207807	0.5056036868107309	0.1076754886197445
0.7506727657616995	0.0111290663940321	0.0934869619742980
0.2468907094157850	0.5012546275134296	0.0979858851271964
0.2442478948582830	0.0067756559919585	0.0965451040205485
0.5004568446084270	0.7578243031890358	0.0992351376257107
0.4956467638962990	0.2596715241280694	0.1022132637562995
0.9965583863047349	0.7553255833860421	0.1003214110405396
0.9981707717675042	0.2532527866403863	0.0930856135616321
0.7576225624770787	0.7762042049654574	0.0920007073815095
0.7462709106259557	0.2564514073060671	0.0969911482167381
0.2564201619974494	0.7683443782881189	0.0949200273733315
0.2473627196110861	0.2555747424644395	0.0952929158321997
0.4829751321942246	0.4859431539236490	0.0969656289119668
0.5069626821264801	0.0070118388112712	0.0952230242552608
0.9906664877457587	0.4945662038573088	0.0925438678829731
0.0047748474893600	0.0093770905473672	0.0918204175743890
0.9113240710622740	0.8397531940440383	0.1496340230667441
0.9070188657159733	0.3220864177225372	0.1480774864443448
0.4141224095269426	0.8333777729598995	0.1535497625157171

0.3879720386817624	0.3247319857826670	0.1484221724288987
0.9220640681583711	0.5892723778187746	0.1506961178720501
0.9208581705298264	0.0909710910425871	0.1335859821080575
0.4204218454547084	0.5830817911958386	0.1451052262884033
0.4200302507726973	0.0846972523459426	0.1440157842196343
0.6685071924898078	0.8399472722196761	0.1494389346579305
0.6617756897930328	0.3335889573400305	0.1556514451703984
0.1637773948113498	0.8407820539114937	0.1490501131919237
0.1593087042202583	0.3233958804995818	0.1449655258653880
0.6665896058552258	0.5890996718209709	0.1723729834651996
0.6700886313941250	0.0957198394455954	0.1376681585024586
0.1651335547943059	0.5934973560602360	0.1459893288834959
0.1567334104945067	0.0983328717177861	0.1394035479142636
0.8322871999518773	0.9260347671590474	0.0475892984697837
0.8242388916964232	0.4160603958034808	0.0561634544748496
0.3412002101352162	0.9194986197728561	0.0526188409269722
0.3246891525284568	0.4069869933093951	0.0493574424361931
0.8241888045074924	0.6696996253777164	0.0536907477957032
0.8388896562532084	0.1835301957048472	0.0422028702398003
0.3322628244528115	0.6705980981992729	0.0488392015554165
0.3292169354063667	0.1789134595882409	0.0483390304540633
0.5864069995392793	0.9294373234641613	0.0536846261042811

0.5808365696981694	0.4365347624899586	0.0595362160353915
0.0670493383581625	0.9273233379331302	0.0494512468875767
0.0738406002230120	0.4142877281996351	0.0536571561728783
0.5874715562151333	0.6742837508622058	0.0499110937789363
0.5888257146199719	0.1897520692184209	0.0471934511229944
0.0796099118757153	0.6702010456690519	0.0466883402466675
0.0934732508609436	0.1848127422630230	0.0415942895434756
0.3897576272647727	0.3748061191381888	0.3203297559039317
0.5006617367202908	0.6064882855528517	0.2652731796128833
0.8006782492691960	0.6136326405334115	0.2541538728430434
0.5853825176628575	0.5562661196292933	0.2803505406724936
0.5048158296818946	0.4078065008508390	0.2836996056446620
0.6940535073324804	0.5920200148035663	0.2318026916039389
0.9017724199641749	0.5967452390637417	0.1952458517320710
0.4435591765668815	0.5866488166798682	0.1903932435383041
0.9058752802020008	0.8431906209093075	0.1952876985679328
0.8949000748810895	0.3015863252155783	0.1928463354160377
0.4102101630184152	0.8242594019399859	0.1991254090984907
0.4283385608150727	0.0838361330688225	0.1895639930911442
0.6786498260803583	0.8423766906726953	0.1949756687306614
0.6652211296790113	0.3241989346199978	0.2009899689148757
0.1648256036673026	0.8427165560536485	0.1948133537926276

0.1483516481663149	0.2845783595039485	0.1865928076074580
0.8089183926824212	0.6490553527673539	0.0090630959897303
0.8501485309819163	0.2002917062068787	0.9970915249867582
0.3322075192583069	0.6720666808022732	0.0030365450548623
0.3328178989376718	0.1953478417701427	0.0032386693739150
0.5880882906336987	0.6642983191829016	0.0043928036896022
0.6034309891483962	0.2127003968815014	0.0027455466850662
0.0806804112907816	0.6739433046559815	0.0008399189903562
0.1097582468989589	0.2075566052693597	-0.0028918647232756
0.3171490774521766	0.3056335979920481	0.2976588912894685
0.5621147336624140	0.3701895181270689	0.3071511574790369
0.4763576923817941	0.3680797958849222	0.2355248660792070
0.6317787456907104	0.5962684395377724	0.3262934973968660
0.4214925277648366	0.5482197066753832	0.2890319117329167
0.1549603050654812	0.5951991048511958	0.1913620432107209

Supplementary Dataset 21. The coordinates of the glycerate : configuration changed step on nickel surface. The energy of the glycerate : configuration changed step on nickel surface was calculated to be -392.33 eV.

Ni O C H

1.00000000000000

11.7038002014000000 0.0000000000000000 0.0000000000000000

-5.8513176253000001 10.2171014480999993 0.0000000000000000

0.0000000000000000 0.0000000000000000 21.1695003509999999

Ni O C H

16 35 3 24

Direct

0.7444101243865437 0.7301573834947890 0.0992818898253547

0.7293090956522710 0.2202600469481332 0.0946317823620751

0.2404264054451967 0.7292603920869073 0.1024906820676303

0.2392110301871803 0.2294159764677259 0.0958858225057583

0.7366218752367959 0.9741531235365352 0.0966696286263413

0.7315970325187611 0.4665021180540151 0.1025067187749765

0.2375562967969735 0.9788002020765694 0.0990413636967433

0.2341877790543986 0.4649743339790169 0.1023727595372709

0.9906994502700941 0.7283574106626310 0.1008244310793015

0.9800380896961033 0.2244190694254523 0.0963602800307882

0.4887248044530302 0.7295574594781956 0.0981713578448880

0.4830391129480761 0.2236473624309493 0.0947072669553802

0.9865064639887766 0.9713651236017041 0.0972981182847767

0.9804266034380139 0.4651280846151428 0.1042390667299582

0.4827237156594534 0.9744797374242850 0.0958357020253674

0.4791935904777624 0.4661632620709433 0.1020150047428996

0.8242956121053949 0.8954796616656867 0.1516670209519661

0.8124414752987691	0.3830686333064717	0.1544418735803117
0.3288532442310441	0.9035532729132564	0.1537942616639867
0.3052576651387041	0.3766604795866509	0.1466830602044556
0.8238319279290712	0.6438442700393416	0.1465227901002276
0.8129651897151008	0.1359323350640650	0.1378354164190887
0.3179989911516330	0.6317898665005452	0.1473907062442180
0.3173471302908173	0.1439801140518187	0.1394198727688815
0.5774478433292443	0.9037951012494997	0.1486295813450678
0.5624193138310022	0.3846567922419409	0.1539864891235992
0.0852023762140288	0.9072356153395481	0.1530318584393507
0.0650209622922180	0.3848334401081803	0.1561001609358468
0.5625930298213642	0.1354919573239709	0.1369995285727491
0.0713131653195840	0.6439912811257339	0.1495046853199694
0.0725964808348602	0.1473828372557846	0.1422716998493661
0.9132432296112254	0.8041789682268837	0.0530466053145972
0.8968295751959768	0.3028385005412790	0.0534704180800203
0.3992886636813417	0.8142677005273623	0.0548827906318942
0.3940564843410685	0.3008615154442849	0.0507997258547877
0.9054006040040747	0.5560274312535582	0.0535549325553093
0.8958717440951841	0.0477469366470694	0.0462579943918548
0.4071619762130773	0.5608012293279421	0.0515068155374911
0.3871392508371542	0.0426529799346785	0.0444530726976658

0.6623093003645486	0.8116413544182165	0.0566581132828791
0.6466523355192922	0.3031788589544738	0.0524864379511035
0.1576985459844221	0.8161850840400035	0.0583613501526104
0.1533850444522824	0.3112968854868841	0.0541527740582928
0.6510163334975755	0.5588802726449678	0.0550304111461015
0.6416199057982277	0.0445140802154656	0.0430002680156751
0.1553555517206694	0.5494926701321832	0.0561034415434627
0.1570894144708269	0.0725822104483452	0.0470989962344050
0.5767961967849269	0.6447669177006636	0.1689039318588864
0.7170121130136030	0.6740303813644425	0.2492578819848255
0.4093719064111676	0.6570359568739660	0.2665161592540656
0.3238288449157180	0.4348986841092210	0.3333395550116095
0.6063464650588674	0.6479886434451131	0.2279872264929981
0.4999712006406712	0.6104418367782249	0.2788157482205408
0.4229256800214002	0.4615903755712580	0.2858899694324860
0.8075567903847873	0.6587251023261351	0.1912834881060576
0.3396402108188361	0.6353042561706687	0.1927581226133562
0.9036046225078226	0.5519838973730458	0.0078147133051012
0.8801133176808646	0.0300762424987165	0.0013905308934858
0.4112834962785624	0.5546483737159427	0.0060128245021108
0.3628935372815678	0.0028126381818974	0.0027582979909719
0.6480057920706868	0.5576956288874233	0.0092482602860809

0.6294530013950896	0.0302301705151771	-0.0022439234648874
0.1544316198616259	0.5367797926296416	0.0107437867350754
0.1722450794747808	0.0891053146802894	0.0020717087116048
0.8198014508395808	0.8872643398955092	0.1973133731919903
0.7980080123525638	0.3679930750140164	0.1994073397246672
0.3573511868292084	0.9358650717535901	0.1963494237741770
0.0884810863529190	0.1737096140468656	0.1863129682038734
0.5930236140594898	0.9304993481538284	0.1926172318955642
0.5491317715281174	0.3611089433686926	0.1982620724088550
0.1075390389801689	0.9389461482303904	0.1960926978897409
0.0564428354044421	0.3783588590325568	0.2016146833803669
0.0751490048124859	0.6583964938109392	0.1948212107153885
0.5525044070517041	0.6546026559802098	0.3232177891609914
0.3359035058809799	0.6042675192706103	0.2952162060914480
0.3768912023589373	0.4161726135304170	0.2403388908421572
0.4899122832349303	0.4266471927714712	0.3017234974434423
0.2491712752142612	0.3487411459500104	0.3249254342862035

Supplementary Dataset 22. The coordinates of the glycerate : 2nd lattice oxygen attack step on cobalt surface. The energy of the glycerate : 2nd lattice oxygen attack step on cobalt surface was calculated to be -440.60 eV.

Co O C H

1.00000000000000

12.1820001601999994	0.0000000000000000	0.0000000000000000
-6.0910000800999997	10.5499216077000000	0.0000000000000000
0.0000000000000000	0.0000000000000000	21.1058006286999991

Co O C H

16 35 3 24

Direct

0.7554016809558844	0.7689307647921776	0.0833379494486163
0.7659209555979908	0.2696846951551595	0.0902027742054478
0.2680083394074504	0.7611577094436234	0.0847586462553906
0.2608258545239993	0.2615830849363219	0.0899585194929657
0.7518822294663506	0.5077863097687518	0.0917769988982763
0.7563571489436856	0.0129165938214316	0.0967187966236413
0.2698170621550803	0.5114530918063175	0.0875692719897975
0.2543764969183449	0.0048712861828576	0.0964713289110933
0.5110665730468642	0.7579790852798771	0.0876280819694701
0.5233143217852900	0.2731537070113839	0.0843605227803338
0.0248168036820467	0.7699473494006004	0.0812246733655496
0.0243432186505174	0.2766495310703523	0.0903006090905417
0.5152135975239289	0.5087925643445331	0.0863332034841368
0.5051378364460503	0.0062311295312691	0.0957998553030452
0.0165608197024967	0.5179770944927097	0.0894818794987212

0.0027873259747797	0.0092925812940054	0.0950946639742103
0.8364894896829806	0.9261453399839686	0.1465722345229677
0.8559842834262046	0.4450826096416035	0.1424851387307776
0.3320751612324169	0.9159186802862378	0.1450944437308362
0.3687634753305735	0.4408976294371312	0.1395317527305175
0.8352487693901557	0.6821484802335258	0.1348317265178213
0.8459415002906732	0.1885844313996586	0.1451885917799858
0.3521558434255629	0.6658556308348690	0.1280162561057954
0.3475354143320624	0.1808619289983087	0.1622123040573017
0.5895743622848311	0.9260263396563095	0.1475417660676501
0.6039748478465955	0.4458398358349261	0.1363466014071094
0.0847176333221717	0.9183863745201148	0.1440754270237829
0.1160959315539052	0.4471586931027233	0.1418246813578597
0.5940519655174858	0.6724422283232436	0.1245293523621523
0.5939592702130526	0.1860297325418213	0.1402156125896272
0.0952237558112888	0.6730581927268101	0.1293002734475886
0.0909524686950661	0.1802675813888637	0.1418927108725949
0.9076525427215404	0.8386673274004565	0.0467068623756009
0.9369727426827048	0.3552981991623996	0.0524615932101731
0.4284782237756665	0.8384909125204414	0.0466933743601604
0.4339764298585558	0.3426969459514232	0.0501320457595227
0.9169635437630197	0.5959907430280440	0.0384686843737400

0.9307078271812783	0.1098949876673518	0.0449760932338553
0.4226155173320970	0.5653216455766218	0.0347026337108450
0.4218409753053050	0.0970503105502250	0.0467426318469570
0.6732029195154660	0.8458041912277900	0.0463323334632753
0.6885461910098384	0.3420613756586223	0.0431710890679269
0.1746074247590387	0.8423662228325346	0.0395778974993886
0.1827881569396260	0.3546369366718500	0.0473605299283828
0.6649003025195680	0.5827976149636463	0.0334156788487528
0.6794677399515984	0.1019457099205581	0.0454943093007876
0.1737165457041213	0.5872103422797382	0.0372522762597087
0.1729755726117519	0.0887984815964110	0.0457029251866442
0.5885381709560340	0.4001016495982717	0.3288259177647500
0.2212257106755602	0.1792654029930088	0.2419061621876884
0.5142828821396893	0.1851688154809893	0.2608958079964303
0.4252568852040245	0.2280296088082144	0.2720804698073728
0.4968346266345111	0.3720900424816920	0.2788667243682394
0.3226732683632098	0.1905986873213123	0.2209895494159437
0.5749731880439506	0.1918086737650812	0.1851868277919158
0.1218946046616464	0.1828325897116531	0.1856983540755528
0.9069387927136759	0.5889871270847508	-0.0072331113286581
0.9289237724350908	0.1093423786592763	-0.0009472426602059
0.3977358647341119	0.5199355658679976	-0.0054769826822032

0.4144116529112167	0.0879924545300504	0.0010230002693785
0.6491409058738387	0.5599780572120598	-0.0111139373373457
0.6832047059128593	0.1005913010486727	-0.0003871788464989
0.1614288333252956	0.5779190205725288	-0.0083314771081907
0.1744920489817855	0.0820955919409902	-0.0000397859960793
0.8300095781127268	0.9139368044294491	0.1920648608178873
0.8702870746984818	0.4646638168531002	0.1873332240130163
0.3301670341433872	0.9026718134557229	0.1904570756565283
0.3895514091505491	0.4721771011708130	0.1827221217231556
0.5803042107719117	0.9152289846905455	0.1930358123892631
0.8129731668066974	0.6753378409819584	0.1794416151655611
0.0813744601145513	0.9016324038941709	0.1890984811785405
0.1333108955042692	0.4662595251904513	0.1865931376826739
0.4294931236043068	0.4037213718646355	0.2921291264463828
0.6542938393480002	0.4883557986853619	0.3249134550640873
0.5441725359131020	0.4155601913519243	0.2336255665214360
0.3745251070111629	0.1856642258901777	0.3166392261028397
0.8445683994346934	0.1906120832975140	0.1910724959291328
0.5849297090527071	0.2378756669179732	0.2893007165701333

Supplementary Dataset 23. The coordinates of the glycerate : 2nd lattice oxygen attack step on nickel/cobalt surface. The energy of the glycerate : 2nd lattice oxygen attack step

on nickel/cobalt surface was calculated to be -419.48 eV.

Ni Co O C H

1.00000000000000

11.8928003311000001	0.0000000000000000	0.0000000000000000
-5.9265986588999997	10.3212535711999998	0.0000000000000000
0.0000000000000000	0.0000000000000000	21.1420993804999995

Ni	Co	O	C	H
8	8	35	3	24

Direct

0.7469859607403878	0.4988121499924945	0.1013703665973160
0.7527324739872354	0.0078167423344652	0.0932479490638782
0.2491948397905883	0.5064109138853989	0.0990410769072128
0.2468081415694107	0.0059802944337498	0.1064277797421146
0.5023389815646800	0.7588408481072382	0.0961411401406992
0.4965362020163301	0.2544859676173524	0.1073130259007307
-0.0018859586337237	0.7562113253677506	0.1041974671893493
-0.0020673908887175	0.2547429340735404	0.0997098596845440
0.7598525537004437	0.7737744975298214	0.0906673294523127
0.7457283019804986	0.2518918755324432	0.0961766415480842
0.2524908621429911	0.7652169898741384	0.1015274713841038
0.2477722318324495	0.2623098885679335	0.1080111420045503
0.4933309784117382	0.4939273612215418	0.0935046937670230

0.5093772498783392	-0.0067568202458068	0.0796901647990518
0.9919956411197680	0.4940178246300440	0.0918043223276750
-0.0047902602881739	0.0053354003506799	0.1013766853100463
0.9028018995288083	0.8259476720274205	0.1549231247257824
0.9086567186714749	0.3304477144658178	0.1509912928071570
0.4248129306548753	0.8472533862614293	0.1492728055936926
0.4049578204108796	0.3410221657578173	0.1612829413802741
0.9201240640785304	0.5832985746712064	0.1489351023128369
0.9086559842651224	0.0801261095114118	0.1410003066700481
0.4196439106988064	0.5904122644619707	0.1437281472445554
0.4059879062706746	0.0976387396597810	0.1650726325164636
0.6672045152299613	0.8344839971604486	0.1472671472630109
0.6644563834726976	0.3313579242987839	0.1540483226138530
0.1669783296834332	0.8348005872020527	0.1557966793749193
0.1645044870116556	0.3382203225108590	0.1539679781138422
0.6532971280723935	0.5759126054694143	0.1628957046280365
0.6641517497014420	0.0875956656465861	0.1369666428345226
0.1713229399486140	0.6013198461961743	0.1468003411787344
0.1577760877726736	0.0992346981504700	0.1540508311947648
0.8380130961600204	0.9257290836087830	0.0487098997807940
0.8244311766591119	0.4105498154819335	0.0520937465895392
0.3312042485525435	0.9193163071021534	0.0573476172149576

0.3231558790808453	0.4078669708302680	0.0567984848293182
0.8270724705395394	0.6674128147962040	0.0530171491070952
0.8374392102567586	0.1777033016196310	0.0434878592134568
0.3304332429113367	0.6821573043511562	0.0498819273786374
0.3258604204080339	0.1813376522534684	0.0622223645367558
0.5993758866442090	0.9242023106550323	0.0429562066805645
0.5766184613532128	0.4232669799632874	0.0575411370139170
0.0750643912828776	0.9281065712866541	0.0630162132411956
0.0824255298565776	0.4295698049174268	0.0544886827600943
0.5853783198180120	0.6690426775851807	0.0465336434057707
0.5706412552855695	0.1717058110326369	0.0457851247634087
0.0820175210297613	0.6767701905993569	0.0502702137379723
0.1010994643447784	0.1857597275464797	0.0545846562770687
0.7504490106514164	0.5552374914173739	0.2502243236095609
0.4857325014976493	0.5761606947294593	0.2636265871524252
0.4152187037801277	0.2449866486135316	0.2552908210288206
0.6525952069747373	0.5406744074050697	0.2213344813403134
0.5220971201203555	0.4768815313135540	0.2565898920886486
0.4087919520958850	0.3483985418487789	0.2278943899764378
0.8916881858605733	0.5753757378729710	0.1931006420104452
0.4338501506306187	0.5941234387941251	0.1896533769106818
0.8785809186585207	0.8070931302864532	0.1991101679868088

0.8970090587703474	0.3251459451425159	0.1964371338859254
0.4553708389959656	0.8653347288832368	0.1926138358370985
0.1470729969996897	0.1012092475767048	0.1995145832334454
0.6868921441231639	0.8486154832547828	0.1919784680473479
0.6675266909546579	0.3242985116874152	0.1996419282853083
0.1743541964454331	0.8304089363211576	0.2012589257434984
0.1644103960362991	0.3286286590251657	0.1995413312306187
0.8211526766937153	0.6554813803259592	0.0075154744620587
0.8386387892882601	0.1832750949610665	-0.0022371275161715
0.3284733245197397	0.6930067771389251	0.0045051523842610
0.3270131168942605	0.1951221181396758	0.0169375805331427
0.5870285882725778	0.6671167177587638	0.0006990793538379
0.5724718046673732	0.1946247479932051	0.0015929134093035
0.0863306070907822	0.6927975975280946	0.0051045558063604
0.1240286055795800	0.2081647380053170	0.0104183344937858
0.1654469150653990	0.6063097325555351	0.1923688506623214
0.5400940826588015	0.4499618918499454	0.3036041003384057
0.4218891190323651	0.5481475353728152	0.2974558678601144
0.3188673483671836	0.3477750095472762	0.2443003868234744
0.4058525108135475	0.1805876918122525	0.2203885584179487
0.4579893518815009	0.0591809024847968	0.1779961302974235

Supplementary Dataset 24. The coordinates of the glycerate : 2nd lattice oxygen attack step on nickel surface. The energy of the glycerate : 2nd lattice oxygen attack step on nickel surface was calculated to be -394.53 eV.

Ni O C H

1.00000000000000

11.7038002014000000 0.0000000000000000 0.0000000000000000

-5.8513176253000001 10.2171014480999993 0.0000000000000000

0.0000000000000000 0.0000000000000000 21.1695003509999999

Ni O C H

16 35 3 24

Direct

0.7385232107819255 0.7301652167455852 0.0943078240441296

0.7338078050978006 0.2232275846245724 0.0949235316783434

0.2354322128345483 0.7323050651487621 0.0996409817845604

0.2379311260535816 0.2337054218582172 0.1008744448548952

0.7325788433489403 0.9742841414101527 0.0937084408173696

0.7334675420321918 0.4700141424764168 0.1021990837192038

0.2333176699553305 0.9758726005530413 0.1047045685205736

0.2289864660065465 0.4724414104935288 0.1042177801991112

0.9885140661613745 0.7295562635708585 0.1015174297342922

0.9776654787594743 0.2239599775807707 0.0972782946620905

0.4846291407323692 0.7310813190205951 0.0942212694781949

0.4900986567573254	0.2256717242083231	0.0982439396900181
0.9833631521204698	0.9713145566719144	0.1014930192024316
0.9799352812306911	0.4674844950861353	0.1032929054393357
0.4835088046614509	0.9770059206362181	0.0959793376380002
0.4832922336899578	0.4696983582514189	0.1021288609253369
0.8162863055540519	0.8942584539334506	0.1496512867499900
0.8133713031606997	0.3853029137301118	0.1547707609486824
0.3268960989523877	0.8990305082182168	0.1561572196692837
0.3241059467372617	0.4030001405931898	0.1617662895862685
0.8165854896817831	0.6365593012503926	0.1456928694594556
0.8114183568027935	0.1344244187864034	0.1375217933819199
0.3143052953533620	0.6426056418571117	0.1446966015251394
0.3373388941470351	0.1449665689478047	0.1579744277986481
0.5736165579609307	0.9033631049175804	0.1468940005557370
0.5748413917328679	0.3930741030010246	0.1537298877782698
0.0675992388793964	0.8949305545055151	0.1589706050049109
0.0661938897836542	0.3902229927479290	0.1569923781829484
0.5693525437195376	0.1384521299590260	0.1388074192966890
0.0694029824280495	0.6455632327240056	0.1489520240465488
0.0584891968582147	0.1392593030404594	0.1468482246117482
0.9180204190151967	0.8150483935402213	0.0545068109385722
0.8975886890206296	0.3036040904726974	0.0535836433082167

0.3952624768634309	0.8186346770708600	0.0533341512438200
0.4060707419451278	0.3032793835884674	0.0571786745607954
0.8966526958184391	0.5536073171903521	0.0534064563570291
0.8981949346521891	0.0483490915315936	0.0464279659288099
0.3954841172793915	0.5517915806482828	0.0535126109877977
0.3953425469865138	0.0542952432393587	0.0494499660228293
0.6542349516955069	0.8158373177977974	0.0511596206075659
0.6572467001508228	0.3166151325180017	0.0541885344163162
0.1492193648584952	0.8091775279770719	0.0568976891073231
0.1464170172764388	0.3166122584283541	0.0583417295358192
0.6495246144460466	0.5557493039146572	0.0510515465159493
0.6435193312574454	0.0490957277195755	0.0413300405175943
0.1488927785861833	0.5559853767398418	0.0529736512163692
0.1509432366060440	0.0622485919465360	0.0549638372813893
0.5680708931422718	0.6414127199260168	0.1607676461601766
0.3927251936578945	0.6354905221529825	0.2619942252180230
0.3625348931170123	0.3137446284400435	0.2512924179483384
0.6732245523386928	0.6287476590358233	0.2477926425447096
0.5718366544798988	0.6109742962232817	0.2196072757729760
0.4406243707904031	0.5431204060030526	0.2558815058812183
0.3338207515765940	0.4090668136118217	0.2271009711688565
0.7907052812209500	0.6391279960780621	0.1900679047265028

0.3294497395891668	0.6471913222857145	0.1907322618241781
0.8887635855446224	0.5427885210887354	0.0078878587527216
0.8888647728809398	0.0321185149581188	0.0012577996503964
0.3865748214951709	0.5356848739709930	0.0083530842039465
0.3903285827366191	0.0423772449701305	0.0040013170576964
0.6387730338305131	0.5397757806219337	0.0059356644910748
0.6321733912692762	0.0264976216966255	-0.0032044373570068
0.1428513764644427	0.5405023289760431	0.0077989064995740
0.1503821050352660	0.0544244900224440	0.0093425492432650
0.8090127950136642	0.8890297898048134	0.1952931831769880
0.7992092548431481	0.3712468658136918	0.1998161873346826
0.3490295229716373	0.8995419484203128	0.2002981081756056
0.0395230453050497	0.1379473869670971	0.1915829496946989
0.5937344732178998	0.9350330208190513	0.1900523708397276
0.5763051010261607	0.3876445949984259	0.1995105066904448
0.0662343664259551	0.9040183037468746	0.2043798880620778
0.0655036837496689	0.3940720193218508	0.2026906077964362
0.0766087259053676	0.6660869988320187	0.1936709687107874
0.3709696605690767	0.1063435087955774	0.1883676743101378
0.3309896387268790	0.6055574848510247	0.2971461682166242
0.4625361563009759	0.5211068911066586	0.3032963832746048
0.3453105317433163	0.2507130650836203	0.2159314472171303

0.2378804475975445 0.3918268235047686 0.2472055761830647

Supplementary Dataset 25. The coordinates of the 1st C-C cleavage step on cobalt surface. The energy of the 1st C-C cleavage step on cobalt surface was calculated to be -439.10 eV.

Co O C H

1.00000000000000

12.1820001601999994 0.0000000000000000 0.0000000000000000

-6.0910000800999997 10.5499216077000000 0.0000000000000000

0.0000000000000000 0.0000000000000000 21.1058006286999991

Co O C H

16 35 3 24

Direct

0.7646751304365289 0.7703478447399273 0.0821816549816241

0.7705359302730005 0.2703751926632993 0.0839751871769461

0.2674584686310680 0.7591320597395110 0.0909990203609650

0.2600310743589696 0.2654046237469363 0.0991634066223894

0.7727191615676452 0.5187989951293253 0.0722604533880647

0.7581932010285563 0.0042909079637669 0.0944264356058258

0.2568306164438565 0.5074593570216063 0.0921374174765427

0.2658928201413854 0.0055016735983605 0.1027812616282855

0.5001683741095244 0.7525020382223310 0.0918557932238328

0.5225035799362291	0.2538397404896728	0.0635621798890976
0.0337005768878097	0.7706565074415529	0.0783206754844012
0.0166621674922324	0.2662087008171532	0.0882350697913533
0.5062488546714716	0.5083704711845486	0.0750207965018857
0.5129496750210067	0.0046472078994719	0.0963063650214327
0.0222615631166222	0.5150150344970986	0.0866389962994490
0.0090650767672982	0.0015332803809202	0.0943880024829272
0.8426700129768130	0.9166770655837845	0.1454663761602587
0.8552365484961695	0.4558447298856068	0.1298176927711422
0.3521874143291200	0.9328608594742389	0.1518781140856553
0.3327399030105818	0.4326411609686810	0.1449294824793158
0.8159821758675972	0.6547002368940491	0.1308930049496359
0.8568055044556838	0.1852940372222478	0.1385962404303346
0.3388377560309031	0.6686607126526496	0.1328183475652558
0.3170388671011735	0.1777412279678288	0.1783292774356375
0.5989914872333617	0.9272348599550395	0.1501232491273889
0.6895293824287576	0.5263173564117191	0.2436069232462999
0.0905698487088327	0.9151789302036716	0.1461707306451105
0.1162218230682445	0.4531600467602604	0.1484723038447046
0.5811649701202578	0.6593868289929121	0.1331277539305057
0.5952559733887491	0.1933802748280868	0.1345596335364669
0.0988732266630121	0.6717845161668804	0.1276565163028088

0.0908904419977753	0.1806560820408335	0.1383405153832138
0.9287999480544474	0.8329993406662933	0.0455928945214383
0.9487132530584557	0.3563571046412187	0.0490719053941950
0.4273908262727905	0.8367304112594266	0.0507774865098043
0.4208739182197552	0.3315216386576005	0.0636288388617723
0.9434123601734958	0.6018676735685095	0.0285925620634325
0.9264016282424054	0.0879971643132903	0.0432982475774984
0.4024941107310779	0.5737101605468469	0.0374927527749594
0.4205629387511770	0.0774957489858618	0.0395318236613993
0.6634537796536712	0.8283444373193434	0.0492231855774610
0.6939260791998995	0.3639019842979714	0.0373740047035499
0.1980639274721832	0.8478774072152303	0.0456288529548780
0.1748020202035793	0.3499643475434276	0.0509130980838267
0.6762242413071153	0.5811131020078965	0.0294402177741498
0.6789655343232175	0.0931965907347353	0.0434554751873939
0.1778880546430592	0.5697726613389585	0.0368735042610148
0.1947306837525748	0.1032589632263494	0.0546278144746607
0.5222940128259440	0.3873089565268620	0.2998668984919626
0.1891453912840510	0.1980540350262957	0.2514289832940049
0.3935026264944760	0.0714289417485498	0.2742044654858585
0.3073583546682079	0.1099431756210198	0.2849788685251952
0.5835326966804777	0.4329914357697630	0.2447316189838515

0.2679102379893548	0.1639494971538078	0.2340692513288684
0.5732930959505040	0.1960858075460165	0.1784319212622041
0.1085819281444979	0.1930510000137133	0.1848616044088000
0.9469699119335484	0.6048284140686623	0.9826385213334304
0.9228385628736018	0.0817163227713394	0.9974797645952149
0.3819367267579193	0.5743863126394987	0.9931377812149482
0.3863522416445825	0.0331723293571583	-0.0001860888256513
0.6723976993601365	0.5672376274028373	-0.0161130683492622
0.6697235354629288	0.0793388663380090	0.9980597792103104
0.1495031632207187	0.5138141164637410	-0.0000158985852613
0.2142024218651646	0.1140075400283589	0.0096178796824398
0.8360276084585414	0.9005312714279328	0.1906578245454687
0.8658867518566816	0.4915557316889296	0.1721623224341594
0.3572592598665955	0.9413355439459032	0.1975923151031913
0.2973575639945734	0.4219967942640183	0.1874110082310451
0.6010850996013605	0.9328769417477675	0.1959299228296688
0.7792054221712742	0.6215449375469496	0.1728384430543674
0.0721638291036131	0.8801704806092927	0.1886065960182566
0.1459787266849394	0.5184186990278059	0.1812619713485555
0.5649341107859871	0.6463568469973873	0.1782992782689134
0.4391279070528045	0.3106738094522973	0.2920887228505417
0.5300121667520986	0.3797308074068966	0.2021372970565767

0.2620963019812102 0.0921045542867679 0.3313383410219234

0.8712429787677259 0.2018420692410607 0.1836906508374556

0.4083400339473495 0.0387853630144076 0.3134823911327939

Supplementary Dataset 26. The coordinates of the 1st C-C cleavage step on nickel/cobalt surface. The energy of the 1st C-C cleavage step on nickel/cobalt surface was calculated to be -417.96 eV.

Ni Co O C H

1.00000000000000

11.8928003311000001 0.0000000000000000 0.0000000000000000

-5.9265986588999997 10.3212535711999998 0.0000000000000000

0.0000000000000000 0.0000000000000000 21.1420993804999995

Ni Co O C H

8 8 35 3 24

Direct

0.7526082315087540 0.5027129764497866 0.1072960038658857

0.7505111061672981 0.0049792889922121 0.0846083589620588

0.2453958026230650 0.5024403766470368 0.0956178037560700

0.2451229473955874 0.0087743595163745 0.1038943204608164

0.5057710277912032 0.7574444672335342 0.0946262945895090

0.5083369870945081 0.2609400767711898 0.1065455107908928

-0.0000165921791538 0.7652299910959742 0.1059965757408646

0.9945244607061489 0.2525345620568046 0.0880751465893999

0.7615450495429857	0.7667338658082505	0.1060787950039618
0.7557617342230227	0.2538178299132626	0.0956754420670090
0.2551998406714769	0.7722392821021622	0.0914761611183385
0.2382920907557388	0.2501907134405855	0.0985727086172712
0.4841588104636993	0.4988323777131565	0.0654481497446018
0.5081725541725135	0.0023917328139639	0.0865375576215353
-0.0042107535893839	0.5016558099894739	0.0961994807038504
0.9769290870888763	0.0011692080492031	0.0522733794860608
0.9197512990511763	0.8419919116806860	0.1639684695972290
0.9082474180077763	0.3017091626098558	0.1531352715424033
0.4172303207433716	0.8355095995540452	0.1463614935909995
0.4223652681997707	0.3351043482792662	0.1902724835622900
0.9308775441775932	0.5867611465687232	0.1528066672234255
0.9223109290750889	0.0854281335588891	0.1206694507727124
0.4278403671386073	0.5786743955249898	0.1351787425023161
0.4270320212861332	0.0886959409568147	0.1474192075048769
0.6702144181300271	0.8467075355659377	0.1469483321239538
0.6720204156468441	0.3287979990036550	0.1542455780388602
0.1703689434469975	0.8423464720893241	0.1539123101942263
0.1595256992811430	0.3264906380820716	0.1452556938181798
0.6824004526277566	0.5874363042201366	0.1762501056026832
0.6809154606341572	0.0920587938560155	0.1343073787130896

0.1648656838149928	0.5934601516606195	0.1435715487655677
0.1819692406454403	0.1138762886560025	0.1577988489964030
0.8064716505376718	0.9087427857934524	0.0346597661623438
0.8367839687309107	0.4148331309964222	0.0575450714442525
0.3300460727709807	0.9242339348116883	0.0498537699998023
0.3127874493256023	0.3980997097466659	0.0460599391880058
0.8291260803425289	0.6686625375825465	0.0586251865150382
0.8364749374475189	0.1840976558852991	0.0348320579756165
0.3308487894682020	0.6715441135345288	0.0462424960206339
0.3388218257081983	0.1880053204472162	0.0596609521458017
0.5871931764744703	0.9265797020060422	0.0446895526667691
0.5797571026084544	0.4252255044493674	0.0635758918575794
0.0792051090836866	0.9335001078382994	0.0538003654157622
0.0686577033253613	0.4188951011167908	0.0544688725933046
0.5921867240403325	0.6775688164498513	0.0420863142461179
0.5878214631467670	0.1836454734291143	0.0454652656819226
0.0805689722363050	0.6813185345971703	0.0493454929863387
0.0885787614922676	0.1898126706302016	0.0318879460328211
0.7837871149309026	0.5469036986911782	0.2583795658954137
0.5472367971454741	0.6470828051864674	0.2627916776090803
0.4644470723057842	0.2399006080856506	0.2714799608601546
0.7056939780586672	0.5804866894197029	0.2354714667140387

0.6365489153983772	0.6134204522555843	0.2819583426293426
0.4200561345503001	0.3154976088086925	0.2465357710191135
0.8969113125547838	0.5626124313895983	0.1963643584737418
0.4572071494441866	0.5751277932003459	0.1776093096129824
0.8975875034011251	0.7964391443309408	0.2041732980656831
0.8649822976925483	0.2254952031976325	0.1805130330032657
0.4249955770597278	0.8571879029201300	0.1908315750153421
0.0868845297960075	0.0622581836977922	0.1621707805162713
0.6753566249690497	0.8643086648446415	0.1918235492310253
0.6793908689876677	0.3254458976083041	0.1997905409698778
0.1690894267769842	0.8392523818364106	0.1996134819203277
0.1385480002713066	0.2941994683142408	0.1883167516539981
0.8258007639727859	0.6678296693687527	0.0128687985897917
0.8497948479309637	0.2188061547321581	0.9922310648047719
0.3281398289559518	0.6714023554607355	0.0004248954650154
0.3481759664051928	0.2040805313127700	0.0143561060864019
0.6306055579335437	0.7207076871397075	0.0023238958281585
0.5951290675402889	0.2017207242216540	0.0004353980432782
0.0782232951059985	0.6881833215743839	0.0037388771650735
0.1266187240563097	0.2397481732032340	0.9935667577965903
0.1490300197846026	0.5933211339185607	0.1885435770660257
0.6609459711087943	0.6210340551854101	0.3320700418083612

0.5150188757079655 0.6725768864183086 0.2991817909072191

0.3814913194052840 0.3565380799237157 0.2817312736309966

0.4606585183515745 0.2418941887052586 0.3175548007392394

0.4419425553153560 0.0923669314733829 0.1926182008799764

Supplementary Dataset 27. The coordinates of the 1st C-C cleavage step on nickel surface. The energy of the 1st C-C cleavage step on nickel surface was calculated to be -393.01 eV.

Ni O C H

1.00000000000000

11.7038002014000000 0.0000000000000000 0.0000000000000000

-5.8513176253000001 10.2171014480999993 0.0000000000000000

0.0000000000000000 0.0000000000000000 21.1695003509999999

Ni O C H

16 35 3 24

Direct

0.7457247054066458 0.7325357925415688 0.0865805459222967

0.7383551188529268 0.2241457433872553 0.0914064114921759

0.2401992307216455 0.7335504114430403 0.0980235595601493

0.2386294234639111 0.2271900687242699 0.0916350307881472

0.7371491969012001 0.9756627146609392 0.0896896930498707

0.7377225972931923 0.4694499745318882 0.0983106076269555

0.2380853119225210	0.9741232984680940	0.1010245511583176
0.2313316837660389	0.4752649245386087	0.0999050680003718
0.9932530290993070	0.7275675529034031	0.0973538728168399
0.9826735953153484	0.2236996893621949	0.0930080987513919
0.4889849095020679	0.7352732640320937	0.0937053364808878
0.4933265834974398	0.2288624185279501	0.0956389560803934
0.9864279902471398	0.9679133103030718	0.0966330259358280
0.9877251783776637	0.4669114366687474	0.0979222254754454
0.4872903074539072	0.9806950479561171	0.0928819816385545
0.4959591887248615	0.4769163041565379	0.1020382061733359
0.8184561677719426	0.8917380383071157	0.1458382095264823
0.8212568405475956	0.3877536983415116	0.1536173402109237
0.3346359841804571	0.9061542506464088	0.1546690637676603
0.3141354280063343	0.3893124255711082	0.1820157440110152
0.8203631284842170	0.6363012880244866	0.1406776703277705
0.8160128331265573	0.1356990375373560	0.1338114129575225
0.3192472609191866	0.6503794013439383	0.1465946211279947
0.3290952076129013	0.1506706602277915	0.1454208729694871
0.5799797723053687	0.9089343699143604	0.1431881406979016
0.5805742790905426	0.3975003878550789	0.1539619551083185
0.0718600974489510	0.8923889715183492	0.1549701593223081
0.0663134556512320	0.3781882843306176	0.1624596104145767

0.5734331528616454	0.1408194312002884	0.1362798420225143
0.0745553975209102	0.6436922286591092	0.1456936461524628
0.0646634608621735	0.1392093365156025	0.1403589057594162
0.9226055757340391	0.8100154341481992	0.0489440783856538
0.9023633167481967	0.3041088844913223	0.0494836129420780
0.3970232721325228	0.8211768324069286	0.0514310131188329
0.4125267387672231	0.3134440096184644	0.0547215177086487
0.9010461452657876	0.5506260205922554	0.0496254906348088
0.9003707699324894	0.0461328428840022	0.0423090327331752
0.4051965410803686	0.5558281706733574	0.0557457664786618
0.4006781810487994	0.0594592710671936	0.0457919838190651
0.6571711317695593	0.8163406114698138	0.0481032752973835
0.6582654725817637	0.3152724820287017	0.0517676544038031
0.1556401649216008	0.8073983652009941	0.0537315404503603
0.1507498135994791	0.3124097671675873	0.0541698614832660
0.6550418233565679	0.5561383395816497	0.0466261445173406
0.6467941520397864	0.0523026406808502	0.0377839781433670
0.1559899500790426	0.5625145236482536	0.0492388432987183
0.1537554877107636	0.0548425015245711	0.0484351207049795
0.5600239938796120	0.6370564691585719	0.1668456047574098
0.4720294169388324	0.7470102294124654	0.2625423707327811
0.2146261799798228	0.3111830858650190	0.2745814812643504

0.6683048793831150	0.5953837634422703	0.2456831127867107
0.5977810091119985	0.6421664609447317	0.2247686141946425
0.5604062868779521	0.7048385486546883	0.2732627651839521
0.2599277114189718	0.4031646120050886	0.2283448167759780
0.7969274421952655	0.6312691417872601	0.1856934688071610
0.3455960851490057	0.6850749177995537	0.1890253365184423
0.8929928468376434	0.5397237732221599	0.0041211510594692
0.8889273369758471	0.0295425700068150	0.9972154939778732
0.3999151664497819	0.5379693669996305	0.0107955729427840
0.3966356942265530	0.0480353426078781	0.0003490552185425
0.6354885300832627	0.5336214060010825	0.0023397792848545
0.6366393037100401	0.0345394453084152	0.9927732496916063
0.1549196492281339	0.5560057135032930	0.0036073730248298
0.1475588835019670	0.0405807902492764	0.0031858899667361
0.8166238301658686	0.8945088810003503	0.1915211441720775
0.7812606792836567	0.3581828607589957	0.1948101596054787
0.3650115357071572	0.9491142868460526	0.1951941304082448
0.0567412870372627	0.1496137119914211	0.1854012579188859
0.6017209717084007	0.9454138559078058	0.1854203557386954
0.6059126933325817	0.4366609160715894	0.1961039143695504
0.0674780707814144	0.8972250378525886	0.2004802588236239
-0.0031943530332672	0.3736650574794954	0.1895586452980135

0.0815575324980729 0.6626751895316635 0.1905445536118304

0.3458184773310733 0.1750297203011240 0.1896202548631283

0.4651125050454867 0.7906853126054700 0.3001889272043445

0.6000527216805834 0.7151002485473189 0.3208300942107811

0.1727378617281321 0.3368439590661907 0.3071283924058304

0.2509430114935794 0.4903069529693708 0.2373695355982257

Supplementary Dataset 28. The coordinates of the 7A. hydrogenation step on cobalt surface. The energy of the 7A. hydrogenation step on cobalt surface was calculated to be -437.85 eV.

Co O C H

1.00000000000000

12.1820001601999994 0.0000000000000000 0.0000000000000000

-6.0910000800999997 10.5499216077000000 0.0000000000000000

0.0000000000000000 0.0000000000000000 21.1058006286999991

Co O C H

16 35 3 24

Direct

0.7522834198893729 0.7698743814578024 0.0871498921998331

0.7570201014226506 0.2638685307456871 0.0877334416157803

0.2508192011554736 0.7453179632208471 0.0841407535619473

0.2460586423464971 0.2554707672939072 0.0948033142618858

0.7627824307958511	0.5153092414583276	0.0854786865088929
0.7445702577884316	0.0030053262056450	0.0958740766886160
0.2506606761240099	0.5017242226232400	0.0918784388976318
0.2557972187634164	-0.0013504879731694	0.1052375758511255
0.4893677672832240	0.7506235507853740	0.0939845297154479
0.5088986518165988	0.2553025829837269	0.0736942587261883
0.0217672126585574	0.7685264643667374	0.0819783684517483
0.0077174035244331	0.2607567520856732	0.0848616803384475
0.4942454528964291	0.4966662867181704	0.0972008392160418
0.4991183350922018	0.0002598189126213	0.0995102761649202
0.0100601220532813	0.5088068382176716	0.0856120196746003
0.0035400455640202	0.0073326826202177	0.0945308080475656
0.8386132567945299	0.9259262783135813	0.1460012270118665
0.8516107010883888	0.4493760560046007	0.1367996319373164
0.3449260312871396	0.9450044198129472	0.1701302437705589
0.3267174625927420	0.4347388791678899	0.1460131464509613
0.8319306739722923	0.6691902095018853	0.1366503198755138
0.8418976849071902	0.1767522528198010	0.1401851478213489
0.3242465310748351	0.6640969303912413	0.1323576864400644
0.3127394213557659	0.1723244452219073	0.1621148587342043
0.5830998922488125	0.9218356204256291	0.1531802220385850
0.5834607535583417	0.4286500107504401	0.1702351895563199

0.0804446923324058	0.9165163688897738	0.1473556818809790
0.0980284413413556	0.4329135618097988	0.1403762221977901
0.5777460176164869	0.6706449379453121	0.1412681143795430
0.5855667405306382	0.1830658926315066	0.1374513476322063
0.0907245614179710	0.6647508673662375	0.1273523731546056
0.0798425123665134	0.1706608339972423	0.1333824493242719
0.9220430088972449	0.8457935172054728	0.0493863639190252
0.9321549068099697	0.3499763750919470	0.0474473499368657
0.4143963693628120	0.8340703699482288	0.0523070927631926
0.4069322241200690	0.3296718150951052	0.0585092882591225
0.9278341313940301	0.5994668987057159	0.0334793577231408
0.9155412448358633	0.0874688921643873	0.0398063911485795
0.4043793626078393	0.5724431216200724	0.0443552013983540
0.4096494178853456	0.0761860265481158	0.0461150727895244
0.6551370253272062	0.8286276056216353	0.0516926681379802
0.6827070570210747	0.3650530546886617	0.0472135825748760
0.1822978462981640	0.8398002338978253	0.0468892133045613
0.1780650674482323	0.3454720561854167	0.0482563996371567
0.6683779702159722	0.5837454221891762	0.0461343204068085
0.6650804304454886	0.0871210672492624	0.0447481334699488
0.1689790966366057	0.5639648368979060	0.0366671416117854
0.1725587849562272	0.0792705416245344	0.0496827174473467

0.5668083924407985	0.4764089001253076	0.2712142868701778
0.2638865158007010	0.2601916951141749	0.2446965162750641
0.4613294817040414	0.1106380382123604	0.2524154154639176
0.4001686204184107	0.1770355896588666	0.2645909189976387
0.5831248474060334	0.4035945150885479	0.2268947922487979
0.3275004068064764	0.1989949738986517	0.2219629491726046
0.5667935535541934	0.1788995041258554	0.1828510707417104
0.2758374378888487	0.2683011510819224	0.2904368476057888
0.9195549023664052	0.5955162330057117	-0.0123378558997299
0.9175925729278881	0.0786859812651722	-0.0058186992878374
0.3894293086523120	0.5547349267994027	-0.0006353686543853
0.3811700613151922	0.0410274220018868	0.0040926102805244
0.6568915526539038	0.5670398077420844	0.0008367454058024
0.6595713003332402	0.0740111702019516	-0.0007026036395916
0.1518235316418519	0.5249739201245913	-0.0051063318141311
0.1774685224951062	0.0795817972800133	0.0038400667983496
0.8382404094362408	0.9196484929464059	0.1917641982317874
0.8751514961558396	0.4850348070780259	0.1791497657875114
0.3027276056213634	0.8583849989665776	0.1861508231277913
0.3020843156154324	0.4163199839077714	0.1903008791580645
0.5749526294684932	0.9290915258011855	0.1984297300632200
0.8308556141790451	0.6591034573474409	0.1822789739030657

0.0708036297908732 0.9026033479293900 0.1927153026723780

0.1140954961910154 0.4557318036547933 0.1848542748800291

0.5698586508856915 0.6711864719758591 0.1870140061658427

0.5640531992518401 0.4402451274713735 0.3128551338308723

0.6187199587596676 0.3421609163199977 0.2444754845560451

0.3987515498636822 0.2008068607776118 0.3146585205467988

0.8537354060891370 0.1830600310617258 0.1856970682895306

0.4083493813642828 0.0367334525295710 0.2194819925672893

Supplementary Dataset 29. The coordinates of the 7A. hydrogenation step on nickel/cobalt surface. The energy of the 7A. hydrogenation step on nickel/cobalt surface was calculated to be -417.67 eV.

Ni Co O C H

1.00000000000000

11.8928003311000001 0.0000000000000000 0.0000000000000000

-5.9265986588999997 10.3212535711999998 0.0000000000000000

0.0000000000000000 0.0000000000000000 21.1420993804999995

Ni Co O C H

8 8 35 3 24

Direct

0.7529570244416858 0.5001143541442481 0.1023704554892115

0.7557901550512938 0.0078836047028555 0.0849972788344535

0.2468856517446462	0.4996513882267158	0.0946871200191513
0.2504344514357649	0.0061215881147715	0.1054005579895019
0.5073298663903396	0.7550198030710182	0.1004683545623903
0.5106712038682094	0.2590827949136724	0.1120886587619864
0.0014801344059092	0.7623946309395953	0.0997820385415389
0.9966702238293109	0.2494275027053861	0.0904304536895038
0.7669130923655658	0.7683544349575661	0.1130486737866051
0.7588641565167031	0.2526901777004671	0.0982518809723709
0.2570465722781188	0.7686024385020959	0.0919104259599574
0.2423468025635712	0.2485694522711372	0.1009925532049253
0.4858747692198445	0.4955341878956908	0.0708990359378953
0.5107788067255195	0.0006139586105173	0.0924477312655395
-0.0014704782737260	0.4985043331776387	0.0933204304892415
0.9820556422106390	0.9886268125816030	0.0737302994662141
0.9199492369784321	0.8516929890861600	0.1511461459764559
0.9131728201220179	0.3046265965386339	0.1537285789757984
0.4164743082367448	0.8315994678719798	0.1499026132143965
0.4319297166252285	0.3392774796991115	0.1800739310762059
0.9293964104819308	0.5862609099389684	0.1462568903549192
0.9200072185785119	0.0845086601301936	0.1278014727951808
0.4220173847193915	0.5741383832123237	0.1380373862612344
0.4281820442918244	0.0842399115680208	0.1536755404765140

0.6572046004621229	0.8210025919392374	0.1679042926293853
0.6805695781452503	0.3326516615328057	0.1560689558287603
0.1658141028177718	0.8360221019949818	0.1522323557130473
0.1621163024136135	0.3255260493335825	0.1468862828964921
0.6872812145772818	0.5925910584675861	0.1684459567444576
0.6859991362756795	0.0901285210151204	0.1377534231450814
0.1656978428521040	0.5907086724361230	0.1424072217274603
0.1876205686790885	0.1112124634496247	0.1596762930532193
0.8085001552161336	0.9035163058948075	0.0434413703955748
0.8381824047322359	0.4095030127635745	0.0560886894359651
0.3365520269832240	0.9228367525679354	0.0522502749079660
0.3151461283444398	0.3940526534789993	0.0480803214186595
0.8285157790834398	0.6702415708139171	0.0547963302784910
0.8366732371022236	0.1787501803796612	0.0372135729518959
0.3372621429892557	0.6716480141600977	0.0468800729903412
0.3414865600434485	0.1846996782147961	0.0624642581002054
0.5902361524478477	0.9228837019850217	0.0503124044885109
0.5806539536943763	0.4221954483222246	0.0604859781077806
0.0904614511568081	0.9353861958957685	0.0507881404835600
0.0710261811704540	0.4138345055880540	0.0536626413225274
0.5984157887823177	0.6774754542450732	0.0517132474522730
0.5890282953194113	0.1819313014645109	0.0511335631312488

0.0853288979621144	0.6784605309586265	0.0462283724061025
0.0837946261663205	0.1763125741997682	0.0382190928902396
0.7594151239918535	0.5285861591053531	0.2535396681432454
0.5348105976297660	0.6569501628512037	0.2542691722382218
0.4580923689795953	0.2839909553138945	0.2784157610264950
0.6820178850322193	0.5780449350447989	0.2291487513546951
0.6067494929977771	0.5998953299335813	0.2709455928240340
0.4269805308282391	0.3525749780395572	0.2375094865079149
0.7592195904406449	0.5351136066823996	0.2994343168775826
0.4405728219952482	0.5754036329765664	0.1833264062137559
0.9123215447646890	0.5698609468309656	0.1912784881839594
0.8727359303309656	0.2309594554090312	0.1828478543786517
0.4210277978211112	0.8437085513960174	0.1953009079455564
0.0958168724675331	0.0663768220787179	0.1719108457361180
0.6986927646017544	0.9097227604402139	0.1838675053208473
0.6929561935221262	0.3415011647343142	0.2015127676722577
0.1624209225544971	0.8320260952608285	0.1979134479059175
0.1428900329416286	0.2979366300352868	0.1906934890998709
0.8220651274261386	0.6776315261272549	0.0095005668010638
0.8454461092480403	0.2033828649954429	0.9930143067913628
0.3394930289759103	0.6768425624840230	0.0010924903413623
0.3521624383084170	0.2013951784546847	0.0172457798218015

0.6372466869004880	0.7224163536839470	0.0122829547139685
0.5926229581290825	0.1999227347642213	0.0061938539024511
0.0863828579196702	0.6855241186039409	0.0005506323523924
0.1139066417692369	0.2125953955119060	0.9962917276413440
0.1550450107325324	0.5877488022164606	0.1879308930378515
0.6116853249858153	0.5847678955346796	0.3217747038757028
0.5879081965910362	0.7292557849604001	0.2209331433000977
0.3808517167963202	0.4044032830025781	0.2582773479663802
0.4501888403576059	0.3090259985178265	0.3214052699512487
0.4371262777355996	0.0849807233476297	0.1992322294734004

Supplementary Dataset 30. The coordinates of the 7A. hydrogenation step on nickel surface. The energy of the 7A. hydrogenation step on nickel surface was calculated to be -392.80 eV.

Ni O C H

1.00000000000000

11.7038002014000000	0.0000000000000000	0.0000000000000000
-5.8513176253000001	10.2171014480999993	0.0000000000000000
0.0000000000000000	0.0000000000000000	21.1695003509999999

Ni	O	C	H
16	35	3	24

Direct

0.7398047104110783	0.7262655636141347	0.0955384474445714
0.7291137542653103	0.2117201929364682	0.0932982504535327
0.2320921880268881	0.7189247735074034	0.1058568670519194
0.2311159825445821	0.2154846706682290	0.0937265014243336
0.7358768952123407	0.9606545527136807	0.0882276731485025
0.7288050956370171	0.4586287092501725	0.1012274087428593
0.2308300982173800	0.9614970599512096	0.1060086595674260
0.2275919668726883	0.4630013188735339	0.1096070395386322
0.9866484336914858	0.7191736571642435	0.1013831319576357
0.9752078523014495	0.2126704726972320	0.0922210099409894
0.4851207239835975	0.7245532748675656	0.1030222454316331
0.4858295747973219	0.2169622855265891	0.0972720504597263
0.9784370820943576	0.9579015471055571	0.0929838028064221
0.9768605531777056	0.4547075213993571	0.1027419567088469
0.4789584661710954	0.9652894074541590	0.1022560450549537
0.4865695828007308	0.4658884573570261	0.1070369195572975
0.8192706221255102	0.8869372885139896	0.1377076370814774
0.8088765080166338	0.3728049668928098	0.1541854609439268
0.3232120061245050	0.8929489122865047	0.1606675304544859
0.3174756460060681	0.3919679036918458	0.1848864964694880
0.8176262389598297	0.6409049904818483	0.1455455626970210
0.8089173202380041	0.1199886022173911	0.1334578025668574

0.3110814957280899	0.6388317818680982	0.1552866411018464
0.3179888971285492	0.1409409714676579	0.1458327285439558
0.5657437621301586	0.8970824778193041	0.1656851392405302
0.5653307107366481	0.3810993322470450	0.1569810585032909
0.0627208339178415	0.8871946317145281	0.1570070630419549
0.0575661234127811	0.3696034506520994	0.1579908172770193
0.5667905412573276	0.1304316846573599	0.1394761299419796
0.0646330671630209	0.6328437361663219	0.1518346615948705
0.0550721207345592	0.1242275576003712	0.1379538149897434
0.9164594130290000	0.8024921853794678	0.0500890043396703
0.8929673238273587	0.2937159908673703	0.0504248785446554
0.3913505755333477	0.8056106164477761	0.0611494164084865
0.4015303928239303	0.3042500558153843	0.0568752789664517
0.8995442499546393	0.5469932133057321	0.0536508911324069
0.9024990027035196	0.0457119273599641	0.0364218921518586
0.3977434398786417	0.5472287171392511	0.0625524743320712
0.3923836118128861	0.0397837160525606	0.0506270722670341
0.6499634894559049	0.7953461073436171	0.0527801447776138
0.6445289679490259	0.2941301116250248	0.0519369730673472
0.1501696529678965	0.7942525980106705	0.0597869210019029
0.1514852154810490	0.3046119900094705	0.0563393848094622
0.6513616429199141	0.5602500752338222	0.0546408090762944

0.6345751571902228	0.0229016856413198	0.0451912348527970
0.1502044977186544	0.5477416023257449	0.0564900573586686
0.1444626776714241	0.0389336691038431	0.0505726553867573
0.5647846835410992	0.6374196069886912	0.1664475142291728
0.4555580231239210	0.7544868194455211	0.2579906500707100
0.3276559456436147	0.3552521220235482	0.2875964019231975
0.6435568319039828	0.5803087341903590	0.2515275693109252
0.5708687669476616	0.6377576129858947	0.2278442184904112
0.5168672227237476	0.6843173219164942	0.2711452555908568
0.3108693710236345	0.4226594880417125	0.2399148536726513
0.6558427601396616	0.5988668621391200	0.2966418288676611
0.3338815676644345	0.6707456371465140	0.1990600333819071
0.9011521732088664	0.5437653131927949	0.0079545223311580
0.9008242909612753	0.0463309125756976	-0.0093297999357958
0.3874722215221728	0.5292821634950967	0.0175736317545137
0.3837295114380693	0.0180654692396499	0.0060448443416302
0.6410373400145868	0.5626449144961072	0.0092199769928459
0.6094572156734536	-0.0117908670258365	0.0025811130844565
0.1520367121332783	0.5442158924186559	0.0108087288270877
0.1377832602467581	0.0229172678971278	0.0053765583522419
0.8091540650293497	0.6505047953184713	0.1907371431685050
0.7854018776421989	0.3456442067069538	0.1976312421091673

0.3512871880791097	0.9168927416181576	0.2040775419086446
0.0374007383235597	0.1183542044542373	0.1830056130827773
0.6464071438483228	0.9758615723509920	0.1795069870417888
0.5838794338193154	0.4024713369231238	0.2014165073003042
0.0504298732522793	0.8919672572322553	0.2019192044463471
0.0271032367466937	0.3628822489906758	0.2010525590624017
0.0745551799696589	0.6525407897824865	0.1966993561082999
0.3366444327999490	0.1697218095921364	0.1895086991951090
0.5061177520861978	0.8211820485232953	0.2189908521141216
0.5305936981708536	0.6732789563271444	0.3216348350388643
0.3221522083731025	0.3934708468458512	0.3276358445896085
0.2768149095346772	0.4912158322599522	0.2530268699458613

Supplementary Dataset 31. The coordinates of the 1st formic acid desorption step on cobalt surface. The energy of the 1st formic acid desorption step on cobalt surface was calculated to be -437.24 eV [-407.50 eV - 29.74 eV (formic acid)].

Co O C H

1.00000000000000

12.1820001601999994	0.0000000000000000	0.0000000000000000
-6.0910000800999997	10.5499216077000000	0.0000000000000000
0.0000000000000000	0.0000000000000000	21.1058006286999991

Co O C H

16 33 2 22

Direct

0.7517038684575887	0.7618694205708949	0.0879987339519146
0.7608312867690543	0.2631536850534343	0.0864828027864265
0.2607090939447463	0.7529743965025978	0.0893142279413015
0.2447793268420572	0.2516631925199995	0.1041680917716498
0.7628120265638368	0.5156119550931061	0.0823861208795937
0.7464464953060963	-0.0036119182533300	0.0945868129419849
0.2485615342502828	0.4991196512507607	0.0953975575062350
0.2603556216103460	0.0036706330579559	0.0990280340173660
0.4915113837848055	0.7464307246972844	0.0929177485233373
0.5126743335511647	0.2518389903058216	0.0664488265966252
0.0372447650404955	0.7706064938071431	0.0727629852622180
0.0087803271590486	0.2538635922377965	0.0912169009575158
0.4913731801948779	0.4958155452630102	0.0897135874782981
0.5044038195679776	0.0002767521566230	0.0918708809034510
0.0128674535071036	0.5060413924011512	0.0875130984797576
0.0019500622087561	-0.0009731406668201	0.0962632029711414
0.8363911629566073	0.9166435180292284	0.1477163089172636
0.8492023602314449	0.4491885206744937	0.1339694492038863
0.3423390499618281	0.9256014830085865	0.1472115583307535
0.3272974652844874	0.4329473534790556	0.1491101302090695

0.7934128946625323	0.6429145269663464	0.1435737588840917
0.8456777211954992	0.1720304579936942	0.1385850595991241
0.3291509711010961	0.6634470105724244	0.1334689844730578
0.3074608660797698	0.1600627602553229	0.1658470171318065
0.5868456058906593	0.9216452440080649	0.1500048260795872
0.0819192235023071	0.9076959199745733	0.1450050878618724
0.1071201112927589	0.4447709517488359	0.1508606713764225
0.5734361051319504	0.6607232094073868	0.1389894925117702
0.5855818680852110	0.1847324972750990	0.1333726557431813
0.0899878152798696	0.6671917386927304	0.1253236748244215
0.0761041037359759	0.1664655380287530	0.1371860645747393
0.9226001824496135	0.8368238275922284	0.0516642927826359
0.9395337684360119	0.3457672482111611	0.0523714418763965
0.4200190358699395	0.8280851560819333	0.0489300421123510
0.4055894444058295	0.3231037131929848	0.0660171872298554
0.9283618572515101	0.6001795103971065	0.0302837087326306
0.9143329811877889	0.0731636884491774	0.0421860011567634
0.3964343693459105	0.5647266779111066	0.0426852999993413
0.4150365290618377	0.0770868921324026	0.0363392256095189
0.6562221880964050	0.8210350053968926	0.0503622130256335
0.6868986630584751	0.3654463520749139	0.0434974917409266
0.1940128648972394	0.8423974881539006	0.0393039545930762

0.1708341380502436	0.3404268902760398	0.0548544681677515
0.6642341049050240	0.5785282616913648	0.0421896892322872
0.6735463646905260	0.0879944312880894	0.0422558669170583
0.1679908456347012	0.5570917491878913	0.0375948839179295
0.1723646209720381	0.0807121660673936	0.0509487632469261
0.2490849899968729	0.2344143087873156	0.2500836438915660
0.4561901612291821	0.0940865661835233	0.2524025802172311
0.3841303445538647	0.1514996459353446	0.2699306574036579
0.3178367800334316	0.1789312308359894	0.2268756246051692
0.5522956050287866	0.1717334985470184	0.1765280016173252
0.2703270593380498	0.2532566245710530	0.2947644266625686
0.9153746018935207	0.5958307090345942	0.9847583873144119
0.9147212194988984	0.0604646591391703	0.9968496887164077
0.3773877559241517	0.5544171227801830	0.9977356194119978
0.3825302671025411	0.0406338289043490	-0.0050552280983103
0.6593846077883986	0.5662780135948117	-0.0034802329808054
0.6708439592319319	0.0781999682719659	0.9966013252510924
0.1400119151790032	0.5000862885803157	0.0011524639475229
0.1782896458155420	0.0926970482080352	0.0053998741720973
0.8359871948961891	0.9068840774411315	0.1933042988079680
0.8610500680506383	0.4776018229139786	0.1777978059513092
0.3564581443312971	0.9375844648025653	0.1926079018379454

0.3030449772128756 0.4239093755162937 0.1935057612473111

0.5784287728392130 0.9277277985484641 0.1952917261279546

0.8754797446907041 0.6820576584002633 0.1648212933842743

0.0879214974252307 0.8981514055199894 0.1902810144546354

0.1379781609722394 0.5074340866990981 0.1848473786436519

0.5645191992088672 0.6592470628290498 0.1846919361875983

0.3722745576575586 0.1581590205075382 0.3207290984140265

0.8618437959609908 0.1794793601972554 0.1838669153319211

0.5286638346766758 0.1265695110340762 0.2807416724485616

Supplementary Dataset 32. The coordinates of the 1st formic acid desorption step on nickel/cobalt surface. The energy of the 1st formic acid desorption step on nickel/cobalt surface was calculated to be -416.58 eV [-386.84 eV - 29.74 eV (formic acid)].

Ni Co O C H

1.00000000000000

11.8928003311000001 0.0000000000000000 0.0000000000000000

-5.9265986588999997 10.3212535711999998 0.0000000000000000

0.0000000000000000 0.0000000000000000 21.1420993804999995

Ni Co O C H

8 8 33 2 22

Direct

0.7531755548602068 0.5008393766214334 0.1014995981952712

0.7534405049939640	0.0042342774365358	0.0935091440282089
0.2475690110980019	0.5006227811641991	0.0949873152092117
0.2505048680065192	0.0060231175364083	0.1027406561182666
0.5074171803091502	0.7538769699811956	0.0977793072549814
0.5118336802011154	0.2544410790014554	0.0989906931777516
0.0025484041001981	0.7629521267353714	0.1013830994488581
0.9974307525368291	0.2504028284908983	0.0939426171460824
0.7677273395276184	0.7662847146073132	0.1128094416563166
0.7597164496719495	0.2534792549214420	0.0962853197371032
0.2584649531437042	0.7703155455055064	0.0921172075819370
0.2444275077942600	0.2502848619560258	0.0965165700410329
0.4851659174418744	0.4974068030407955	0.0658852462241453
0.5136209418310353	0.0001177330626463	0.0888358138433869
0.9991799400592667	0.4990894375163956	0.0932366575786942
0.9817772926940161	0.9897536194434691	0.0799071276088812
0.9190466528861783	0.8485176799877648	0.1534030985949154
0.9157605420162768	0.3078663932262110	0.1563409213215648
0.4187792242880741	0.8332604422792715	0.1486105403138977
0.9291277661753367	0.5856993843903593	0.1463330607484505
0.9242662243029395	0.0881781963641219	0.1333195094526913
0.4268914920990959	0.5729150678366134	0.1372808408251618
0.4288531620643133	0.0897844398510965	0.1471110363997204

0.6676538231724868	0.8392044107624047	0.1526749904301163
0.6750055060400360	0.3286822679632682	0.1499934302202126
0.1691159702603207	0.8389481043126334	0.1530284585033114
0.1707993067849441	0.3288345234181350	0.1462456002795299
0.6833873581591551	0.5889561755694154	0.1697421445370763
0.6814658059547993	0.0949659931615714	0.1384960651754756
0.1659867787560637	0.5911784255793855	0.1426951571383915
0.2039494662433953	0.1232179204003298	0.1583832064469399
0.8112918670628999	0.9072521072288369	0.0466496356218961
0.8404504979498802	0.4111012293514488	0.0547398330686306
0.3356912907079553	0.9214737356442159	0.0502585216569148
0.3152508960763347	0.3970331602757458	0.0445266624019352
0.8293466526352642	0.6697057010561966	0.0551164029363183
0.8368490943527592	0.1774379966250620	0.0409537939641230
0.3357992845389311	0.6705595228639422	0.0468912944829059
0.3380251352444976	0.1823206407662543	0.0549846975346009
0.5930961780460483	0.9239707247532926	0.0487690745722494
0.5803468197463009	0.4237062578298135	0.0586322365121974
0.0875280811413685	0.9354764296134862	0.0525791860278728
0.0711296833577983	0.4131601200397458	0.0544072241180843
0.5961552623191081	0.6767366124930418	0.0461263269590382
0.5895519959055983	0.1772531312871427	0.0430610449337517

0.0857335883629950	0.6797209278196497	0.0471342922123546
0.0815286033377011	0.1766554040008105	0.0401797318067816
0.7563725137836274	0.5141523630659947	0.2501292406638787
0.5566541079511093	0.6703657090076005	0.2623287993949091
0.6880029814909248	0.5780576612523353	0.2306806039370116
0.6342175471203445	0.6145500187547952	0.2771795073226669
0.7607041675515072	0.5167192710238242	0.2961413624749929
0.4510610746149452	0.5740243208303892	0.1813619523049742
0.9088633975625529	0.5663334749058597	0.1910421187493476
0.8733350900381707	0.2341828595852546	0.1852853059528106
0.4256490070790075	0.8513477302554517	0.1934992194307434
0.1171105447870851	0.0869544115782944	0.1768078940559474
0.6551041767169991	0.8240715395248456	0.1979643801360423
0.6855454241873276	0.3332703666256040	0.1956125182494194
0.1670766214903786	0.8368758131067986	0.1987590458351036
0.1635746751847869	0.3084928807946177	0.1910701923593552
0.8240508387941217	0.6793628750706332	0.0099327907103039
0.8476319484607384	0.2047325442623051	-0.0029427909481967
0.3355467710723372	0.6731841095833565	0.0010887629812742
0.3398704929494684	0.1929770114757959	0.0093452766646175
0.6355118035458316	0.7217695503403762	0.0068832061419493
0.5916242635116270	0.1873467216103697	-0.0024802124359377

0.0866240216134457	0.6888348965972906	0.0015589851926838
0.1018988854126977	0.2080152983250787	0.9968847894482342
0.1524320795414429	0.5871491049494085	0.1880370418070167
0.6398554584492673	0.5943538621443806	0.3269622106403543
0.5550651518263809	0.7182729525149988	0.2991201284495562
0.4448907020053196	0.0954883410419987	0.1922088094337068

Supplementary Dataset 33. The coordinates of the 1st formic acid desorption step on nickel surface. The energy of the 1st formic acid desorption step on nickel surface was calculated to be -391.56 eV [-361.82 eV - 29.74 eV (formic acid)].

Ni O C H

1.00000000000000

11.7038002014000000	0.0000000000000000	0.0000000000000000
-5.8513176253000001	10.2171014480999993	0.0000000000000000
0.0000000000000000	0.0000000000000000	21.1695003509999999

Ni	O	C	H
16	33	2	22

Direct

0.7412803947567204	0.7308768439898640	0.0948792086678571
0.7304710726086044	0.2133950674513490	0.0941206928652300
0.2322339423526044	0.7191659115124998	0.1074369897405891
0.2371352089049633	0.2183307201274232	0.0885755874075236

0.7332240200313508	0.9627058996320126	0.0897770133937606
0.7298088800391676	0.4619185262632977	0.1016788807010925
0.2328096169717441	0.9637012629331305	0.1078015700723685
0.2229897389013366	0.4614517699941780	0.1005337241824149
0.9871527408845608	0.7225556600407194	0.1024586025633706
0.9745412339087194	0.2124095342453737	0.0910230744514026
0.4847758614648628	0.7211686143009305	0.1056148280672953
0.4893285889249652	0.2182627259232704	0.0977621791590579
0.9790790229458655	0.9611783828380475	0.0941006376875025
0.9769223944212335	0.4573234249672536	0.1023094487126159
0.4808850399740009	0.9660047606120191	0.1039912517000020
0.4904200883915785	0.4656651621488633	0.1060115498638319
0.8182427636271387	0.8894580312139809	0.1380729788711388
0.8118655264464795	0.3766293089385019	0.1542186099282754
0.3220799302650542	0.8918717357553413	0.1630689630518540
0.8182490162388513	0.6452854122314051	0.1454325895124084
0.8105431536429967	0.1227502037873571	0.1342379615778660
0.3056658595443150	0.6216398358423146	0.1515683031653317
0.3245062071770584	0.1433738886924200	0.1468705750273246
0.5630969382072524	0.8947130743150472	0.1635069040888534
0.5665161140896819	0.3844021272448667	0.1589568605009200
0.0632762659040326	0.8896327630128674	0.1581282817000970

0.0679883953419865	0.3752473450412786	0.1544319453100052
0.5684309504568924	0.1314598541139688	0.1409533826497307
0.0640027439404381	0.6341756510988544	0.1530512421216494
0.0529346691731801	0.1255628329484239	0.1386699378179969
0.9181627365517568	0.8068730252497940	0.0505943791228312
0.8939617720703111	0.2956180046960418	0.0504139339711167
0.3937455266560879	0.8046835208961779	0.0637855549108054
0.4146444264050327	0.3081444462530918	0.0553921846674980
0.8992067379314714	0.5507912126808122	0.0539819779889000
0.9014282605846438	0.0469283417400786	0.0369073816442015
0.3954097470588073	0.5416199650495405	0.0605333715770417
0.3941651558636763	0.0384157345137371	0.0519250268918098
0.6469047649705278	0.7966894700708085	0.0540004587133340
0.6478505621728483	0.2969986638555289	0.0532621726368195
0.1504556927671414	0.7968186937378878	0.0616879220690764
0.1475678140700187	0.3061701908845529	0.0533994851447621
0.6534735625966308	0.5656579784941206	0.0544921055196963
0.6383378096222179	0.0296071494949988	0.0448373535100931
0.1480872576660920	0.5532550718773680	0.0538967361064446
0.1442406221818078	0.0384079803040856	0.0510864689946838
0.5522574217914928	0.6264697799916105	0.1684667285344900
0.4207529233832480	0.7118618310797633	0.2607859946299544

0.6597485349060163	0.5957492578733303	0.2503863308464806
0.5674409226773097	0.6313870593672041	0.2296914540681196
0.5067904766819762	0.6674374452090425	0.2746334258453269
0.6677474232778405	0.6065059280296266	0.2961471575426343
0.3105408527376301	0.6195566339635393	0.1977863018381200
0.9008235915590918	0.5494201970296254	0.0082592398868043
0.9033443094651917	0.0496181809191351	0.9913178614330538
0.3903471365375067	0.5232627463622698	0.0156632585179320
0.3862015995329182	0.0146199656366626	0.0075045432246657
0.6423516502788463	0.5663557490255946	0.0090007854355152
0.6211585876877525	0.0046992384007022	0.0005928741921837
0.1486622900206339	0.5513712440201500	0.0081135446894780
0.1331387673044580	0.0142836889734222	0.0067835215778789
0.8090207613398696	0.6574880721394172	0.1904103293847058
0.7945011619392058	0.3493343061526574	0.1979088419671847
0.3468528361080639	0.9231097471810149	0.2059955342312271
0.0239175787605345	0.1140882655668971	0.1823834338789579
0.6382005461709706	0.9702636849810899	0.1836088711335823
0.5953830548657496	0.4168382132116061	0.2017942163782291
0.0505208480346064	0.8910982403898881	0.2031918128205609
0.0739620863197795	0.3917775856097351	0.1994119319798807
0.0743024975734926	0.6554144410085121	0.1977524411316863

0.3444298233416792 0.1633335785355350 0.1912338562165507

0.4609666742541093 0.7773670306215187 0.2250854940439638

0.5303530968694123 0.6677118624965392 0.3247946387468020

Supplementary Dataset 34. The coordinates of the glycolic acid formation step on cobalt surface. The energy of the glycolic acid formation step on cobalt surface was calculated to be -437.30 eV [-407.56 eV - 29.74 eV (formic acid)].

Co O C H

1.00000000000000

12.1820001601999994 0.0000000000000000 0.0000000000000000

-6.0910000800999997 10.5499216077000000 0.0000000000000000

0.0000000000000000 0.0000000000000000 21.1058006286999991

Co O C H

16 33 2 22

Direct

0.7607537645639452 0.7693412144736749 0.0880473940782792

0.7525245869372348 0.2582342862435764 0.0905557997843591

0.2528752674049191 0.7484429233787118 0.0826330408398109

0.2441610979437341 0.2640450869678349 0.0923977271131911

0.7668451343146425 0.5137296914409650 0.0869100941870755

0.7420453016923062 -0.0013197074615206 0.1004438910200481

0.2520023314250621 0.5050486476207472 0.0877804393224784

0.2590956421339956	0.0002605720697841	0.0974536691306734
0.4930833786615093	0.7531970175776269	0.0907523416184459
0.5279086888083306	0.2569575533748935	0.0639191337118355
0.0263940875023351	0.7686676339592480	0.0855734480283291
0.0060467848689524	0.2605425238562960	0.0914148430445225
0.5007289489213947	0.5040110561179498	0.0803348533948501
0.4997690264538752	0.0050188864401625	0.1003028512620406
0.0062265967277572	0.5093873613018050	0.0911523825646189
0.0019717026725365	0.0088472221604931	0.1023765708358665
0.8229509026789181	0.9256955121056918	0.1497792717360217
0.8368844362123847	0.4326099781711649	0.1448017200812690
0.3495306876103682	0.9568610587223909	0.1647941486295402
0.3328873494215380	0.4359649328780485	0.1388823858591110
0.8135680392036988	0.6623906950488374	0.1432123427512234
0.8212615077748964	0.1629409443685097	0.1432107094480838
0.3302348351813814	0.6649882367844954	0.1293100644935434
0.3183895595279948	0.1833922874928496	0.1781056335057020
0.5915445905163854	0.9273517154018175	0.1511880457505974
0.0888567411959496	0.9205382297155903	0.1475723708990268
0.1043971912727217	0.4351529080833899	0.1403153402250193
0.5739618346889215	0.6636287642411028	0.1357621396357817
0.5938207694640584	0.1896299230500577	0.1278977847292797

0.1031854480406379	0.6824767366306841	0.1317909407620519
0.0852287955625421	0.1854935206440298	0.1390483854902825
0.9190387547262274	0.8380939807008728	0.0538846559781129
0.9131007534217751	0.3280767111038870	0.0538167167967225
0.4194861223921371	0.8402648152774905	0.0523014659633500
0.4209588494518789	0.3216382874961214	0.0663399827216235
0.9296738556507101	0.6045941439615951	0.0365569398661415
0.8969223944701936	0.0753821531396990	0.0518213285146589
0.4022750683033990	0.5744943858215988	0.0364148999104142
0.4145279706869306	0.0810583057198932	0.0392906796408947
0.6637038626150186	0.8450832083479798	0.0553666581493556
0.6906319537695593	0.3523902885855949	0.0377498699421233
0.1862360515556178	0.8428371813508816	0.0445779074849499
0.1718417565654188	0.3462633152064672	0.0446620030040841
0.6722706130014385	0.5973874243767967	0.0382959024758046
0.6693955071446482	0.0709228869994433	0.0476444676633659
0.1698451171676743	0.5693456138001275	0.0361622985609920
0.1782370808895931	0.0923583278513279	0.0523849872408342
0.2814023320698694	0.2817669065434054	0.2575804828600340
0.4428002320537646	0.0980594797136360	0.2634608550929373
0.3625261655516667	0.1396402129164387	0.2848719292750493
0.3213212305728548	0.2022726153466562	0.2348948121631894

0.4104860621402312	0.2082500283643314	0.3237499435919844
0.2932242968221170	0.2895087162599258	0.3034516737327926
0.9292628998914898	0.6069821142477457	-0.0093642639241347
0.8842227209369644	0.0605584484499649	0.0064883021370140
0.3850663648978238	0.5676195011358035	-0.0087419329526467
0.3945489963827225	0.0434190879964088	-0.0026768896034939
0.6801355482614998	0.6001929337167580	-0.0075077392427472
0.6620778667786995	0.0414329631114336	0.0042212089847559
0.1597679904778554	0.5412379466074836	-0.0075997483521738
0.1825554356137304	0.0903820313418965	0.0065231682747879
0.8037676851069051	0.9128565742564818	0.1946731675422091
0.8364873743276511	0.4372427169783102	0.1905853278635254
0.3018011884191796	0.8689577821084881	0.1792416615906519
0.3153165910580544	0.4285331425608150	0.1840488661970888
0.6017465527898563	0.9447478321960968	0.1963519003397363
0.8159636637445883	0.6594186850231738	0.1890247432926058
0.0892718099396822	0.8969868726490817	0.1913835648174168
0.1228765339238364	0.4522533988003068	0.1850989738799870
0.5527035151946303	0.6530091012272796	0.1804839085790787
0.2716556369135514	0.0628084548327977	0.3052972517968747
0.8062683207534090	0.1622931600076876	0.1884702470458400
0.4061802815692701	0.0437556321269197	0.2234416792479322

Supplementary Dataset 35. The coordinates of the glycolic acid formation step on nickel/cobalt surface. The energy of the glycolic acid formation step on nickel/cobalt surface was calculated to be -416.31 eV [-386.57 eV - 29.74 eV (formic acid)].

Ni Co O C H

1.00000000000000

11.8928003311000001 0.0000000000000000 0.0000000000000000

-5.9265986588999997 10.3212535711999998 0.0000000000000000

0.0000000000000000 0.0000000000000000 21.1420993804999995

Ni Co O C H

8 8 33 2 22

Direct

0.7539012182792127 0.4992000728535606 0.1095574045680697

0.7568916488458419 0.0097994241859951 0.0888030261597488

0.2486008048408970 0.5033243562909181 0.0916920534703986

0.2507111704610749 0.0018235398125705 0.1017563215305220

0.5032265571402508 0.7528213324618817 0.1003747136403308

0.5091256481368563 0.2557058378093940 0.0979914821954034

-0.0006767430025504 0.7525270743363378 0.1072245021823831

0.0012850710334977 0.2538664162129644 0.0932937109878703

0.7519223865492645 0.7648531832412202 0.1089604941286094

0.7558749490056089 0.2538963804919980 0.0969919033268155

0.2599567842029003	0.7675432171964132	0.0946877226392383
0.2491440260390020	0.2555635960688201	0.0928177216773436
0.4798528285451862	0.5025311819909637	0.0713089339790448
0.5137195347062422	0.0032014803753971	0.0949432285704069
0.9954937027873646	0.5026034004210870	0.0983720102500990
0.9926716782027719	-0.0000345618703877	0.0939484708500300
0.9155897731207131	0.8370385242844288	0.1599617413384601
0.9088857951144287	0.3194927822737502	0.1502458387396308
0.4145535380789951	0.8274279857445217	0.1537471972546078
0.9327101295310155	0.5751360997611453	0.1507345540192505
0.9158811442039004	0.0756346617134054	0.1375862387761347
0.4250785273075241	0.5667524305444375	0.1328112959452338
0.4310118837249953	0.0878289485196291	0.1461038152999543
0.6497226671325417	0.8150406551254251	0.1689749972437152
0.6647833624459847	0.3283575642033875	0.1554569563941879
0.1710763256113192	0.8374134690520307	0.1534869987879293
0.1708697175508045	0.3377153172638376	0.1450461094913053
0.6816021525869885	0.5821600831532443	0.1873611628964024
0.6759217857547525	0.0890065049593138	0.1371854199176672
0.1683592217980305	0.5941610156401461	0.1448662230841111
0.1641921519567183	0.0942710723325000	0.1479690294116789
0.8313334805357532	0.9183189589137983	0.0511207697610421

0.8335641645253365	0.4124886648343255	0.0567334478562824
0.3472801635550124	0.9217157743516821	0.0543112417133298
0.3269410589881127	0.4109718198909175	0.0367985255830143
0.8262790358429934	0.6670297564352174	0.0630892658261791
0.8388572573312603	0.1762288680078679	0.0399090237350592
0.3360259627571425	0.6761113740679568	0.0458431526407470
0.3392276063560651	0.1713685024646125	0.0521043908159402
0.5889393466215606	0.9213233906817205	0.0532639930965856
0.5791307746505552	0.4329773591849344	0.0662935642613428
0.0711060220523731	0.9258179050900958	0.0583074592595619
0.0703534525516665	0.4336285009453395	0.0536296185615109
0.5979424230741035	0.6810819368163014	0.0467806452816379
0.5992349616127334	0.1864628791815288	0.0456314034694137
0.0871194609764774	0.6807986218012333	0.0491427908550840
0.0825591710746871	0.1683897488137393	0.0408409384982029
0.7135540317056168	0.4863761016553369	0.2708889813809613
0.5512659587915393	0.6777616714821143	0.2679487437059495
0.6750670446031467	0.5667795423376444	0.2445822711098102
0.6316855218278120	0.6352065812049509	0.2915870539870241
0.6993926533178213	0.4823098578780628	0.3165291033355710
0.5812686078233306	0.5675269081446069	0.3311505317935343
0.9174485696984689	0.8563724309592861	0.2045500128470527

0.8877287247329748	0.2913285002376036	0.1938384668846059
0.4105533039570796	0.8104487494947518	0.1987401792713524
0.1451354553849595	0.0982265512964119	0.1922266911461802
0.6847495822568321	0.9040587741781871	0.1836223818786736
0.6729562538559600	0.3322041960732199	0.2010904062774243
0.1802776849038266	0.8357164436721994	0.1988590665732448
0.1812714292981372	0.3512383227836804	0.1902761135213547
0.8222208186620176	0.6634587797737345	0.0173284702467831
0.8422040387959823	0.1848220503112716	-0.0056989941168241
0.3365387015912555	0.6836265470232668	0.0002012844759066
0.3395290179216356	0.1707564498858788	0.0062473965603760
0.6248331156629525	0.7109171061449936	0.0037459457374583
0.6161657270202037	0.2093307558105898	0.0012297453977130
0.0835641081297615	0.6775614852123485	0.0033280014675347
0.0761810088079315	0.1625821439702812	-0.0048627185057157
0.1539648346563605	0.5956093816035593	0.1899429906793084
0.7245209426897670	0.7141866277689084	0.3118840086875432
0.5902278832106220	0.7304880277900729	0.2268585725925066
0.4548827203292275	0.0920710358976401	0.1902454013576779

Supplementary Dataset 36. The coordinates of the glycolic acid formation step on nickel surface. The energy of the glycolic acid formation step on nickel surface was

calculated to be -391.28 eV [-361.54 eV - 29.74 eV (formic acid)].

Ni O C H

1.00000000000000

11.7038002014000000 0.0000000000000000 0.0000000000000000

-5.8513176253000001 10.2171014480999993 0.0000000000000000

0.0000000000000000 0.0000000000000000 21.1695003509999999

Ni O C H

16 33 2 22

Direct

0.7473730623489909 0.7314901400426443 0.0970262111064736

0.7344211044801262 0.2249632444346030 0.0966900832116492

0.2356497291213318 0.7304059145155997 0.0968735384171345

0.2349530706901747 0.2297310268408778 0.0925685903479928

0.7336958669260405 0.9731366376895043 0.1000962009223920

0.7374045407711325 0.4765462741049079 0.0991061563590405

0.2345206508336982 0.9753513563616004 0.1015469460843567

0.2276163074039029 0.4819516383743787 0.0968583505589823

0.9866285790062328 0.7274735526314901 0.0978838474364937

0.9781764954177750 0.2241580015455486 0.0967147957270068

0.4777832076320697 0.7307913526268072 0.0937292782415129

0.4908417158438213 0.2279680244999614 0.0967917871672584

0.9842016772317170 0.9709852182078477 0.1010895800289755

0.9818962463490871	0.4678396782551349	0.1006209751096084
0.4828972788500940	0.9774826297113827	0.0966773752731286
0.4884222810345037	0.4709369482615967	0.0945929952129620
0.8149916271264745	0.8907772066763923	0.1539458404529427
0.8177107184128107	0.3905092819008247	0.1541073912575320
0.3280932566653817	0.8991166785361979	0.1527643920151031
0.8188522995262869	0.6418359957865574	0.1408296082668407
0.8125974646249792	0.1368590767326777	0.1404365710891449
0.3090772005932992	0.6405570762893927	0.1403119629205126
0.3291090452214759	0.1529799617555291	0.1473746489190094
0.5801770071470580	0.9168172154814989	0.1613792343911051
0.5794583522219421	0.4058028630365158	0.1504986326075509
0.0695206231210821	0.8927910005016803	0.1565418827078560
0.0924791598303102	0.4091481431001814	0.1582307642475961
0.5683907895669109	0.1397439166170528	0.1397370539880360
0.0691588543492278	0.6449489104956424	0.1454444029957904
0.0639446734752627	0.1496400415728552	0.1464128165327130
0.9100364671157810	0.8069729519552711	0.0518251703569358
0.8969098874035573	0.3017561315017948	0.0539048183088143
0.3990082937028522	0.8219798417830180	0.0519431614151493
0.4100562986069644	0.3083639471858955	0.0533049272989532
0.8925385534114036	0.5465288775641166	0.0491491194303071

0.8951340527530558	0.0490425120748055	0.0488826670609580
0.3921024276825341	0.5481223082981362	0.0520965095836265
0.3990055774503510	0.0582692242469438	0.0469043018743771
0.6563400715974693	0.8100417662622145	0.0609445209579093
0.6577757014720398	0.3168493056637908	0.0548337418859447
0.1497898723377731	0.8068515628128835	0.0549600674582017
0.1472081691415326	0.3213470765224888	0.0568871481019153
0.6526041196733374	0.5692464929108819	0.0482730195864370
0.6427795232268332	0.0432875326246431	0.0461332338271281
0.1449368886106113	0.5605461638605020	0.0456141827850164
0.1509986558497996	0.0590690077880940	0.0502795372987169
0.5339243735471422	0.6249391849854843	0.1773645104104081
0.4705273676624421	0.7800845455218385	0.2601905258574193
0.6066963355346844	0.5610607989540799	0.2597632353692342
0.5602570691124096	0.6359932666147396	0.2337905828507596
0.5504153691417902	0.7314001601995134	0.2793042302202298
0.6222408977982604	0.5792885200848504	0.3049287220528684
0.5139168501483177	0.6803762389894709	0.3250219894976600
0.8765851080992390	0.5244715220629544	0.0046097034371757
0.8842946096464026	0.0368349205763214	0.0035457155137661
0.3794818813171240	0.5247738210274558	0.0075426760152610
0.3944832792764038	0.0482253498001026	0.0014031119231152

0.6521190106300632	0.5783615850003038	0.0027535779137171
0.6266665922371494	0.0138495254342298	0.0024754534659829
0.1401020964324713	0.5502334956913160	0.0001094686415078
0.1476421254887960	0.0481577537716242	0.0049024667712841
0.8182136614985431	0.8994511651342628	0.1994808319491908
0.8114645254501478	0.3809734309410696	0.1995718886451646
0.3502904741800331	0.9020037328573962	0.1970917491503940
0.0581974397854916	0.1655376486570446	0.1909170544637702
0.6187272208430143	0.0028886078714102	0.1819910173221246
0.6017162477350292	0.4341124115113535	0.1940613598660421
0.0642107497406215	0.8930281177471024	0.2021398328145980
0.1245581594265738	0.4748746976495615	0.1916712985065211
0.0786725993188699	0.6677421062643187	0.1899038768588482
0.3462276209230359	0.1526657823279697	0.1921748252538458
0.5080453033802479	0.8296028142692538	0.2184638373248164
0.6551466087050529	0.8087407448766322	0.2875940943980143

Supplementary Dataset 37. The coordinates of the glycolic acid desorption step on cobalt surface. The energy of the glycolic acid desorption step on cobalt surface was calculated to be -436.95 eV [-354.19 eV - 82.76 eV (formic acid + glycolic acid)].

Co O H

1.00000000000000

12.1820001601999994 0.0000000000000000 0.0000000000000000

-6.0910000800999997 10.5499216077000000 0.0000000000000000

0.0000000000000000 0.0000000000000000 21.1058006286999991

Co O H

16 30 18

Direct

0.7517038532548693 0.7585551262227974 0.0942874893425489

0.7584237274449683 0.2601124359077279 0.0893393339590784

0.2564226935222640 0.7524733386436311 0.0812120796289008

0.2377324555554716 0.2610908113376601 0.0936573458473948

0.7596187774183616 0.5127805633716986 0.0890869005814175

0.7454713386940983 0.9964366650646626 0.1030392750814561

0.2457091417837171 0.5047388111874441 0.0905300958302125

0.2587932843486523 -0.0012563403844328 0.0809603971265544

0.4914442672576115 0.7536288609940410 0.0929928176239366

0.5324909206607846 0.2612742264576455 0.0645447897978704

0.0311901116358536 0.7671508148830940 0.0763767848395974

0.0022709050436434 0.2522440740469586 0.0937918281185043

0.4886688256982666 0.4966066627868745 0.0900660968303667

0.5038041673606279 0.0100853603940062 0.0991829296967304

0.0061933049726055 0.5084669118910797 0.0895839607980598

-0.0000237097255674 0.0009509583853181 0.1018763586707989

0.8219939855082048	0.9190528950996897	0.1525226151448503
0.8351167331483169	0.4290996250652753	0.1414374944790238
0.3512847898959123	0.9754244623382192	0.1519809328060439
0.3209852794605980	0.4321091478324742	0.1437591543453314
0.7762190768145633	0.6385667602575624	0.1541073500841117
0.8373819528870714	0.1631166561203177	0.1433738450009910
0.3276961001375859	0.6677663438688984	0.1299001591127551
0.5952096263412245	0.9331787840743225	0.1538302259757702
0.0953302637081964	0.9199676040583243	0.1394135175485567
0.1041410558488376	0.4447934420272846	0.1459240016303353
0.5637827655844653	0.6623465573450226	0.1397307392887671
0.5997102637465319	0.1925802667337003	0.1266770719048702
0.1016190000454338	0.6836259480698773	0.1277341319753150
0.0852309339918348	0.1790951746792228	0.1396530611749447
0.9082279461051302	0.8216850173077345	0.0581425021034835
0.9212078746816087	0.3303605604364510	0.0549783937867446
0.4245342172370222	0.8432065104864550	0.0524078770775977
0.4138765225883230	0.3115919459104777	0.0800296852458706
0.9233638750894552	0.5949407746837787	0.0356609559498066
0.8954325780684147	0.0634570704988812	0.0521499705851696
0.3965261054468082	0.5671522080542873	0.0393254584621398
0.4249290588557030	0.0890929087716878	0.0313968385498459

0.6613701128995449	0.8416611430192124	0.0592731794609582
0.6913728640678148	0.3549756166127035	0.0344285118138986
0.1876814891968388	0.8375524232253579	0.0339302767870038
0.1612002751460258	0.3449433727039816	0.0490309184023035
0.6575381096767393	0.5856458461634151	0.0448132623725765
0.6796814626347939	0.0776287339193107	0.0511627360942229
0.1691116388383638	0.5698685621737396	0.0356838564623583
0.1742171333217722	0.0940512457661295	0.0461189601930035
0.9118331780887410	0.5835590341689596	0.9901795314326719
0.8782084448596558	0.0395760753336271	0.0077820869643719
0.3804560128952862	0.5568771746227013	0.9940724762770523
0.4197479807184989	0.0643145215114445	0.9873365688729276
0.6602284925168991	0.5868487398880696	0.9988490112371631
0.6826070728700899	0.0602041857966634	0.0064333861518640
0.1583945247361449	0.5362155813891897	0.9929849384403268
0.1758861864096586	0.1024164471562497	0.0003364078036446
0.8023083144752210	0.9067579591914872	0.1974136920026384
0.8248412452499039	0.4240775912658793	0.1871099276263529
0.3071772797308768	0.8990001997967195	0.1775790189899677
0.2985370800301013	0.4280746980495552	0.1881517767433914
0.6074296331475142	0.9602343896779670	0.1977978704313297
0.8496837621990371	0.6741766794327634	0.1818433177311640

0.1132937897233144 0.9069549525437672 0.1826266941551110

0.1320241802514678 0.4977370329228945 0.1837193896139220

0.5535190643598642 0.6604284034065676 0.1853543280080346

0.8506688420060945 0.1671198928745405 0.1889127707362004

Supplementary Dataset 38. The coordinates of the glycolic acid desorption step on nickel/cobalt surface. The energy of the glycolic acid desorption step on nickel/cobalt surface was calculated to be -415.60 eV [-332.84 eV - 82.76 eV (formic acid + glycolic acid)]

Ni Co O H

1.00000000000000

11.8928003311000001 0.0000000000000000 0.0000000000000000

-5.9265986588999997 10.3212535711999998 0.0000000000000000

0.0000000000000000 0.0000000000000000 21.1420993804999995

Ni Co O H

8 8 30 18

Direct

0.7591602606035730 0.4995053825015778 0.1019410838654493

0.7538532955155228 0.0054540968239339 0.0959196299525458

0.2507541136656202 0.5053223830877566 0.0947694221327268

0.2508588408469585 0.0037834492308698 0.1025869111107080

0.5042305453975048 0.7558292506828586 0.0992576493971973

0.5101725464253251	0.2603241771569315	0.1009461609802465
0.0015554062343299	0.7539147570214650	0.1033658434764474
0.0008276870239581	0.2535816203697322	0.0937497639525222
0.7563834028764693	0.7637645561134593	0.0996440337737023
0.7557934315454782	0.2532661803948624	0.0974592572621098
0.2612187249225740	0.7687741193619773	0.0956635804956417
0.2491012178401168	0.2569980795603766	0.0929078945527897
0.4844901512433160	0.5094427585668981	0.0766034731310340
0.5130290817870721	0.0065655151685955	0.0974390763996438
0.9970766911690604	0.5025835819669474	0.0976481741855703
0.9952444343843918	0.0026472938119440	0.0926942038710310
0.9111954576943438	0.8276928545909895	0.1559534860155097
0.9085181548264234	0.3200233060436274	0.1510836396932588
0.4154434921758065	0.8284022718297914	0.1545495928320652
0.9305902301695530	0.5761490734275859	0.1473684780621057
0.9191765339237224	0.0763537554447325	0.1385178498445405
0.4298049521127321	0.5722355891544075	0.1373700765152225
0.4305703068817612	0.0921168981674686	0.1483485368952433
0.6665797801889681	0.8317680202458845	0.1528666986607963
0.6680943060969702	0.3394742427352495	0.1559426532769189
0.1691766358162937	0.8383394040610391	0.1535167149102341
0.1705101338941957	0.3385893572174314	0.1461976658552771

0.6740886445491178	0.0935498598891981	0.1402440782790164
0.1674669074171878	0.5954546902156843	0.1459401698282903
0.1631077839728594	0.0950654097077939	0.1480711048173648
0.8344872796230302	0.9249021591148678	0.0497578091185780
0.8365858284602020	0.4096752863716374	0.0545419337972016
0.3452254902979422	0.9229980292313956	0.0563538501049960
0.3317574661423886	0.4158860879901400	0.0409091542612001
0.8300316271647051	0.6662741477614745	0.0549050891813863
0.8362632144910608	0.1729339593627297	0.0429065181946574
0.3365257728123586	0.6762684466920966	0.0474917977491888
0.3414577261423432	0.1738323093319080	0.0539035018637147
0.5900375762708093	0.9276099759372174	0.0550045224673449
0.5810973839252144	0.4375998040438213	0.0677336592313896
0.0750284523425974	0.9303248929799902	0.0563932146096066
0.0735425121333841	0.4342663270075006	0.0544508057100338
0.5947990711053497	0.6826096420271086	0.0421999257433956
0.5982654311207661	0.1904399211289848	0.0473779985516807
0.0905008528600394	0.6806733378433402	0.0482923722186905
0.0855093012243558	0.1744605025900506	0.0399914009508389
0.9093236068885397	0.8377164166245457	0.2012987723237021
0.8878921043240310	0.2895826465011664	0.1943997738741906
0.4087984220168108	0.8154741600743008	0.1998919732835849

0.1375211190969111	0.0959072031600928	0.1915366283786428
0.6677299049562705	0.8343022448998614	0.1986028196657286
0.6802738565681148	0.3408100586537401	0.2012394181215639
0.1697671512727890	0.8330260476119576	0.1991455384504941
0.1745364486633906	0.3427295208726380	0.1919012597778521
0.8245776298896068	0.6617088634229700	0.0091599120501804
0.8349833682875013	0.1767525771090580	0.9971971750912781
0.3343074759055947	0.6789571699033258	0.0017561789915969
0.3447396814294555	0.1735808142578505	0.0080765089975265
0.6076376953995060	0.7029547420237049	-0.0025803279375138
0.6150903406663014	0.2125527802751504	0.0029074005700762
0.0895573776977298	0.6778217079374945	0.0024796628873386
0.0787258890700302	0.1697415617163110	0.9943040740460454
0.1503367629984923	0.5955975804618231	0.1907922046110383
0.4514497548277494	0.0926038696359372	0.1927217578127700

Supplementary Dataset 39. The coordinates of the glycolic acid desorption step on nickel surface. The energy of the glycolic acid desorption step on nickel surface was calculated to be -390.64 eV [-307.88 eV - 82.76 eV (formic acid + glycolic acid)].

Ni O H

1.00000000000000

11.7038002014000000 0.0000000000000000 0.0000000000000000

-5.8513176253000001 10.2171014480999993 0.0000000000000000

0.0000000000000000 0.0000000000000000 21.1695003509999999

Ni O H

16 30 18

Direct

0.7488208531959343 0.7320333348474267 0.0977798670212981

0.7328341623081680 0.2249665399307923 0.0984802699226344

0.2375859386193801 0.7299929020421690 0.0986977349948262

0.2340893631420042 0.2294962464710837 0.0931544200050113

0.7338362894053897 0.9731179538324263 0.1005878116247621

0.7371821338676088 0.4793119969443094 0.1001862386590203

0.2352923820397193 0.9760551597532393 0.1026172819223653

0.2284707367098608 0.4810833552916490 0.0950654029701161

0.9881239503462746 0.7276594319849293 0.0991200135022211

0.9774911811930593 0.2238039479148294 0.0980079627877986

0.4803370708913493 0.7326150531417329 0.0941568007240253

0.4900560177014340 0.2288014544167539 0.0971503201764163

0.9838206172655102 0.9681629594723885 0.1026142695393963

0.9810786391200186 0.4671663716884474 0.1023751721881887

0.4839780204934764 0.9795148636247921 0.0977719661044867

0.4895913843824028 0.4728994285823869 0.0752328007858715

0.8120833088213791 0.8769979898901795 0.1559756157910028

0.8146427600579871	0.3918620403220638	0.1557748308666154
0.3299090471019564	0.9011438462439213	0.1541673764075705
0.8188291982613256	0.6434248170187676	0.1418295023919714
0.8120143885884360	0.1364509648341779	0.1415985313114988
0.3126371145004351	0.6368497592984126	0.1411135229670042
0.3294746270326521	0.1556524913848256	0.1479769909590349
0.5761235898134063	0.9010975543879487	0.1503794006998059
0.5745481439866094	0.4243155748519830	0.1459481924365144
0.0717910819835518	0.8936312273291093	0.1583135510827091
0.0933029317275772	0.4079224914744740	0.1566812099614382
0.5667917718156715	0.1420076091291460	0.1413456700684070
0.0702715006748735	0.6444483198741827	0.1466984243026140
0.0626154577433970	0.1477435520194159	0.1471978188726986
0.9154180929440348	0.8074759695369922	0.0515150250076822
0.8953020274379050	0.3010017756558086	0.0555311611227777
0.3979570324365996	0.8228882823560683	0.0531418747549151
0.4064354098451673	0.3008934831427435	0.0498653558455251
0.8925107066563566	0.5456881206711477	0.0510348458945017
0.8915111772218789	0.0451500026128819	0.0516494207099356
0.3896788370824622	0.5534441202327727	0.0445920081908682
0.3992794517256054	0.0595699843870272	0.0484453568628688
0.6602669360551120	0.8083551128479054	0.0606710005369098

0.6568021647658532	0.3189610351601700	0.0565210364395826
0.1515314413137145	0.8068225866914843	0.0568701487064042
0.1456521223297715	0.3190945794317832	0.0562032494792060
0.6642773765236346	0.5810703389459410	0.0429362551008239
0.6434868406505917	0.0484682247498975	0.0461291257777079
0.1437048411546074	0.5604564656586901	0.0464115099301006
0.1494474270489035	0.0583212878416245	0.0514789959005924
0.8752406175642740	0.5209527699913261	0.0068223838524274
0.8748687592951763	0.0266555244401880	0.0068547952770025
0.3724405798456563	0.5471188321280994	-0.0005966806427080
0.3954911703249411	0.0523291343092141	0.0028230947736289
0.6875384618787185	0.6123953276285236	-0.0001990120111343
0.6289382701271862	0.0313970489471228	0.0011511891374819
0.1362842189897170	0.5522096979682277	0.0008468816633607
0.1447162873531370	0.0460126078466880	0.0061588881025150
0.8195688600350693	0.8579136871812224	0.1998458292700796
0.8052098598809493	0.3809100910599122	0.2011572388511795
0.3534710893037225	0.9156677700347412	0.1985092679909224
0.0553768325579939	0.1618014595838846	0.1918163665690111
0.5923583148364748	-0.0745616706051301	0.1946362385110048
0.5964193349167984	0.4645279653690503	0.1875566169179189
0.0699018252976744	0.8966855240643782	0.2039549082857693

0.1269015279774288   0.4687689668548172   0.1921277245522835

0.0776084174852211   0.6656219227471852   0.1913298584234058

0.3466398217217432   0.1597736577432069   0.1929664336889797

Supplementary Dataset 40. The coordinates of the glycolate : 3rd lattice oxygen attack step on cobalt surface. The energy of the glycolate : 3rd lattice oxygen attack step on cobalt surface was calculated to be -439.83 eV [-410.09 eV -29.74 eV (formic acid)].

Co O C H

1.00000000000000

12.1820001601999994   0.0000000000000000   0.0000000000000000

-6.0910000800999997   10.5499216077000000   0.0000000000000000

0.0000000000000000   0.0000000000000000   21.1058006286999991

Co O C H

16 33 2 22

Direct

0.9030927392270390   0.0533263817935916   0.1836980146703297

0.9153987496813111   0.5604577430144718   0.1896723132941836

0.4145025709327444   0.0500825715130472   0.1886790439845486

0.3999885733977682   0.5490420127562274   0.1842358536537836

0.9157211985599814   0.8098323964523193   0.1800367403946413

0.9019694506335019   0.2925309704788023   0.1982346200866907

0.4024668201473612   0.7986923201992638   0.1961362596649641

0.4074852951781431	0.2931447705861078	0.1936768189122266
0.6462683463074862	0.0441065739159115	0.1938379172048122
0.6755829564800679	0.5457349792156591	0.1606403510603863
0.1853045035454622	0.0605295260241058	0.1684751764900772
0.1557543839427454	0.5492120869956130	0.1877070688577838
0.6450106132182480	0.7908958067145172	0.1862388955308354
0.6585132297279136	0.2964870736075220	0.1979994768964959
0.1650913518486575	0.8036193756497289	0.1874235185015435
0.1506841081472201	0.2837602683612854	0.1928519724963367
0.9862899110117456	0.2033447137318312	0.2465651308097679
0.0034440606351095	0.7469474238831666	0.2344897579936295
0.4925434251969874	0.2258493521056805	0.2489269273949859
0.4828905367999203	0.7284492818270467	0.2463114774446967
0.9400077987261516	0.9328126055458114	0.2423834621003072
0.0016883156865736	0.4731252409830305	0.2427260974307816
0.4843401677384514	0.9622045153939824	0.2347324884263216
0.4421982440534681	0.4580776053990341	0.2963840458576241
0.7416920657253484	0.2177444125616572	0.2533162566258131
0.2372515997244235	0.2004235365656421	0.2416757649074585
0.2685566367738423	0.7486356359614466	0.2578899578890405
0.7264639486631147	0.9554120961328516	0.2371797044391728
0.7405788544268127	0.4828899590758702	0.2364747643650789

0.2428290792910655	0.9642500362833194	0.2244650332195582
0.2345242429027728	0.4624663142369662	0.2418243163010297
0.0670101427710211	0.1125777672979341	0.1470739150387388
0.0906561927431550	0.6439186325958102	0.1520820647473566
0.5754522404800124	0.1281930050780520	0.1511914370832140
0.5732498154080027	0.6189569943625591	0.1633152853522313
0.0816689976978334	0.8883876195734258	0.1283457905018712
0.0702347020564467	0.3725107881380104	0.1450480569297082
0.5482886721190011	0.8631800321115997	0.1417673263893511
0.5692821621436968	0.3682284386760754	0.1375959297951575
0.8076027579817566	0.1194544900087044	0.1507321623611501
0.8490642204181885	0.6603612970699367	0.1403119089286351
0.3476894345417169	0.1351590243439981	0.1372278791540708
0.3151590938031645	0.6407392841991203	0.1538154708928342
0.8162936349306775	0.8707280309038276	0.1379469392916406
0.8265286342587910	0.3845821299826528	0.1466679858469421
0.3198967978761086	0.8564829514867259	0.1398996607197145
0.3234054385343072	0.3736328575859391	0.1444483948245860
0.4201032093499584	0.5572043904450215	0.3815605191235967
0.2212066369174592	0.5591076582166378	0.3365379764851410
0.2460950050152563	0.4682790308259473	0.3068500961181328
0.3799128125354346	0.4942779226144309	0.3261919161720619

0.7213542483665492	0.5011022060809692	0.2784124083344596
0.3555466426190156	0.5821689709717751	0.3910567553903109
0.2954208785844817	0.7991091407547968	0.1035845310446472
0.3271374470063964	0.3666704441573868	0.0987988387136495
0.9762065804731075	0.1843169007591828	0.2914709021268889
0.0149771388873772	0.7770580990931015	0.2780140653292057
0.4900285089577660	0.2267810491095605	0.2947832444643855
0.4976884417832806	0.7618626499959932	0.2891992437627336
0.7418108959331577	0.2253642180889969	0.2990112709032607
0.0235095118445089	0.9752261043176806	0.2621835716438616
0.2464071269961256	0.1809821711079563	0.2852206747082622
0.3005638188007440	0.8272356505224308	0.2822326218390356
0.7311221361232989	0.9595601791003381	0.2830279423601605
0.1776663445612331	0.3756695066751203	0.3285093959419074
0.0249809265909484	0.4874058722938918	0.2873508891195570
0.2484762670920485	0.6344940223183072	0.3066443082263173
0.5302510037832394	0.8607994044547682	0.0968674813326994
0.8117372640727967	0.8570937836972620	0.0924126481826390
0.0621841491370163	0.3645482175759069	0.0993375900701157
0.0676088964491582	0.8754470776391006	0.0830259621520800
0.5424417375839131	0.3259758897839465	0.0968608872219638
0.8186141784403903	0.3717107560196035	0.1012050674707966

Supplementary Dataset 41. The coordinates of the glycolate : 3rd lattice oxygen attack step on nickel/cobalt surface. The energy of the glycolate : 3rd lattice oxygen attack step on nickel/cobalt surface was calculated to be -418.68 eV [-388.94 eV -29.74 eV (formic acid)].

Ni Co O C H

1.00000000000000

11.8928003311000001 0.0000000000000000 0.0000000000000000

-5.9265986588999997 10.3212535711999998 0.0000000000000000

0.0000000000000000 0.0000000000000000 21.1420993804999995

Ni Co O C H

8 8 33 2 22

Direct

0.9931532374366625 0.2493633769767678 0.0869300600025161

0.0004690103595053 0.7640058688761282 0.1075113651100514

0.5014511842783149 0.2525679602745913 0.1011175505093863

0.5007354794240402 0.7534493979821888 0.0989965358635668

0.2407287913231655 0.0087585557831116 0.0967829872459910

0.2413367513289801 0.4971525736717253 0.0936869600402781

0.7477255884654540 0.0025514754595415 0.0856195169901702

0.7465803609850917 0.4947158322519233 0.1096987899305692

0.9752305180894408 0.9996317794522525 0.0517958760085142

-0.0063422850061509	0.4971447858834314	0.0959946531292598
0.5033970547999737	-0.0044126364611292	0.0914252630930451
0.4719555161429947	0.4922176031418793	0.0629673081041383
0.2441254364877197	0.2530325160404912	0.0923034466759310
0.2511700570189211	0.7677703165652212	0.1000466034454170
0.7514729744269856	0.2491542015425490	0.0991370974574244
0.7526217732487975	0.7621213880029840	0.1042123825594632
0.9905785936146383	0.7998525641411420	0.2616342806414846
0.7804450155253214	0.5848522667227463	0.2783749167416322
0.0806198306416423	0.1817619228755488	0.0268090660689714
0.0792892493388337	0.6795528283353676	0.0539257785387345
0.5860121971530863	0.1765703529349944	0.0480391234069627
0.5857117576957632	0.6762246827600468	0.0436904282310821
0.0635563782104742	0.4151403322207608	0.0522461801918328
0.0732852604171625	0.9255897215678162	0.0542130889810582
0.5774206041615687	0.4271156898963277	0.0696646792963119
0.5817344251872436	0.9226326887497561	0.0472050720386765
0.3366253841483070	0.1800074618736668	0.0504509288404258
0.3293204076872216	0.6728591956235200	0.0505357630962418
0.8339137500113926	0.1804202833839763	0.0367560590892789
0.8264455092551489	0.6643712983177968	0.0625044606605885
0.3080573731977416	0.4001504281746157	0.0376103160710782

0.3208164597836849	0.9159117309820550	0.0552210460569039
0.8311696170671395	0.4101932811876771	0.0606758788966265
0.8034943144263527	0.9065792569036624	0.0350848604318852
0.1957263990216719	0.1176276568837852	0.1528751439556331
0.1610123039703415	0.5858181736753304	0.1455857682775402
0.6777870071801064	0.0877271365635790	0.1371450343341461
0.6829520990862971	0.6031887679285213	0.1905313631628972
0.1552010804407052	0.3214477115107024	0.1396609844460416
0.1618697454115760	0.8266218258053019	0.1693658378345381
0.6611792621561411	0.3232088411848174	0.1579510813872382
0.6678503806257929	0.8452544809477502	0.1489826393004417
0.4150086139625106	0.0812872132749917	0.1478073098002985
0.4220962389101092	0.5711405110762029	0.1373887841209692
0.9209024426324494	0.0827538204690497	0.1205735945889813
0.9236227228277457	0.5813042757651014	0.1540280206152930
0.4145082035104934	0.8326440886879435	0.1525124615607646
0.9044897388211094	0.3021941167483058	0.1535654117058646
0.9128451530184061	0.8346920822591444	0.1649923436323013
0.8840813174480731	0.7936937639784206	0.2258431671002595
0.7722899538937273	0.6515966074343936	0.2293923795102897
0.4273089365303174	0.0963063380883751	0.1930395999309396
0.0535987436833776	0.8014331069105368	0.2290666427605866

0.8516245643667102	0.8527450510395692	0.2530481852651200
0.1537266523698904	0.5839714975402357	0.1912830890694670
0.5940491058193591	0.1909183360253021	0.0027146659248368
0.6207012660162564	0.7220655051612270	0.0040615513093057
0.3409367366752158	0.1900937906136978	0.0048399247874265
0.3315446661728921	0.6808955267578891	0.0048768779146367
0.8209250727309358	0.6571265011261025	0.0168082609048022
0.1401111976515756	0.2940815152343918	0.1835800052280162
0.2269062232521024	0.9095159099973626	0.1871701540090613
0.6643517326442469	0.3134544538426298	0.2032979962669153
0.6753892900438755	0.8701168383781508	0.1930048022286284
0.1035215218717486	0.0763815720623480	0.1635915188133833
0.4235223568665319	0.8512936595595195	0.1973880687525922
0.8706230821707303	0.2313490886061682	0.1841824996035825
0.9313026444149357	0.5614713580312648	0.1977685275774625
0.4399641608314857	0.5520287695274438	0.1795310351005936
0.8688278596209420	0.6468198841113214	0.2955541836118305
0.1097391856855102	0.2234966977832535	-0.0140789020425001
0.8468732805163048	0.2133504472838541	-0.0062044187565505
0.0856586428151663	0.6953501980308906	0.0087395959608523

Supplementary Dataset 42. The coordinates of the glycolate : 3rd lattice oxygen attack

step on nickel surface. The energy of the glycolate : 3rd lattice oxygen attack step on nickel surface was calculated to be -393.84 eV [-364.10 eV -29.74 eV (formic acid)].

Ni O C H

1.00000000000000

11.7038002014000000	0.0000000000000000	0.0000000000000000
-5.8513176253000001	10.2171014480999993	0.0000000000000000
0.0000000000000000	0.0000000000000000	21.1695003509999999

Ni	O	C	H
16	33	2	22

Direct

0.7365034757555501	0.7257960406948181	0.0769674087640554
0.7248202183621714	0.2100138520768094	0.0896616202188415
0.2309132668708707	0.7191469704769193	0.1042502569036993
0.2269949684422188	0.2126921026148874	0.0864267083234047
0.7245487240530332	0.9640683165336873	0.0902351115107007
0.7236976676177128	0.4568815491047897	0.1027572121434190
0.2292931421877326	0.9623365150276372	0.1064918695650147
0.2201328205658876	0.4593571370490024	0.0982917609665724
0.9850991867395973	0.7140720309687669	0.1095261160386602
0.9708960501503422	0.2112224830000488	0.0945347503957061
0.4778903134893154	0.7234588432337883	0.0902355146523522
0.4827935391674327	0.2160969003745649	0.0945596920382037

0.9760632594515356	0.9530366547064523	0.1016526935583523
0.9725566637594869	0.4524033954472940	0.1065478862234215
0.4757309398200296	0.9669322976564432	0.0942176994272954
0.4803873975249380	0.4596873170492570	0.0944538129960787
0.7965634354058735	0.8631369315735901	0.1497572421255232
0.8149541115407364	0.3947389203805126	0.1667868042744092
0.3251052119968385	0.8913347298140264	0.1585058578830885
0.8305854280252433	0.6295962494228229	0.1666874011957471
0.8033388707178338	0.1236413960364604	0.1351943784514512
0.3151672664241065	0.6320077740386690	0.1474596451345236
0.3206660989672084	0.1420470745673489	0.1452475030968483
0.5718909060207814	0.9006162999247055	0.1459528043549515
0.5556783019856288	0.3866609871792699	0.1544849515020426
0.0639598807379724	0.8884061552364254	0.1609000882911818
0.0632274108583260	0.3726888622563783	0.1568990684871895
0.5635271099786179	0.1290790345345882	0.1365625897064248
0.0707550462938155	0.6330656256722741	0.1557425670834151
0.0500730642283083	0.1242361050636675	0.1423510027132597
0.9169025594389183	0.7952404579192102	0.0565494600070949
0.8923496223117989	0.2955834914571980	0.0519528602948344
0.3824590461661898	0.8066069087182891	0.0535536709179783
0.4031230814045620	0.2976425847892445	0.0497955888621725

0.8965428873495170	0.5466363820873800	0.0595203881857335
0.8887405199352761	0.0273431973936467	0.0462922011672363
0.3932058719166251	0.5441488083559692	0.0512407492819218
0.3868206024726638	0.0395294611650306	0.0474181132164956
0.6452410895539106	0.8094734653639346	0.0449382107371940
0.6437521914201978	0.2967570861945216	0.0510553043353734
0.1413929455264220	0.7914048944360892	0.0626709734184815
0.1357361998294905	0.2967098792567165	0.0529628325312607
0.6484970772257563	0.5473064201698576	0.0467120496513601
0.6372160854572115	0.0405100267963421	0.0378137759815395
0.1414865104861436	0.5503991801106244	0.0545287434238994
0.1405554438691828	0.0347397785955185	0.0511447868549315
0.5535464524276232	0.6339742152011338	0.1632069543021710
0.8450388139864844	0.7986257627100698	0.2474989060770909
0.5989824590327655	0.6214808998192993	0.2644609383477379
0.6267863927255477	0.6843169972974221	0.2090969689081346
0.7550212083877704	0.8194670298357516	0.2096785544857286
0.6820639695788615	0.6722199456142058	0.2889508352677288
0.3260864634503128	0.6458961126639480	0.1926879642944842
0.9047666083700132	0.5518830580350763	0.0139322089545102
0.8768446120344803	0.0058174200241875	0.0015745123119678
0.3855662710055746	0.5277280645374469	0.0060824134500477

0.3787084591468843	0.0188302663986963	0.0027309857229582
0.6371600738051417	0.5177756541543801	0.0031670815620999
0.6316401808842933	0.0283141923303851	-0.0075989914420117
0.1367441313355985	0.5477036968155191	0.0088439507916066
0.1315969992080048	0.0119520186691674	0.0067402440822808
0.8385613955238771	0.5571976618827984	0.1864149101177240
0.7680513341729543	0.3142477883708191	0.1909873192777559
0.3543438511542427	0.9205668071374276	0.2012752032294134
0.0382811911476645	0.1284312041775450	0.1875559070374927
0.6034263717402529	0.9624478179222196	0.1810888337396176
0.5681446415083828	0.4040360676547891	0.1994132455489043
0.0443647788129322	0.8940345572925995	0.2050064131303088
0.0522449118600600	0.3761305694878387	0.2020797736874663
0.0836276890158810	0.6555636223419201	0.2003089395217917
0.3406958356622886	0.1600767208780074	0.1897720514471165
0.8529456959489170	0.7300915107465625	0.2196541551072307
0.7349206291304299	0.8882981506466441	0.2368067510419062

Supplementary Dataset 43. The coordinates of the 2nd C-C cleavage step on cobalt surface. The energy of the 2nd C-C cleavage step on cobalt surface was calculated to be -437.61 eV [-407.87 eV -29.74 eV (formic acid)].

Co O C H

1.00000000000000

12.1820001601999994	0.0000000000000000	0.0000000000000000
-6.0910000800999997	10.5499216077000000	0.0000000000000000
0.0000000000000000	0.0000000000000000	21.1058006286999991

Co	O	C	H
16	33	2	22

Direct

0.2966604459732788	0.2791903389701419	0.2037994027647818
0.3095437318943509	0.8016513744283045	0.1820530489294358
0.8025027754950933	0.2938849416621401	0.1893274942268993
0.7943964176202974	0.7941035471519543	0.1879333205312941
0.2944599896797828	0.5469860556880198	0.1754794967024001
0.3357425688067401	0.0623900691820893	0.1680211294662741
0.8163497458136461	0.5449604123770465	0.1568572039125523
0.7905601401179656	0.0406686767208480	0.1903427665630320
0.5561448607605535	0.2944135368361661	0.1927386162020969
0.5463866409916542	0.7911603685955212	0.1949816039817958
0.0441135849989272	0.2915948940434925	0.1999718809947702
0.0603565311902738	0.8104456238667154	0.1779775418767967
0.5585444030291341	0.5539908709986049	0.2056611168074762
0.5597722524994790	0.0456168972959905	0.1848723567898870
0.0586195228037885	0.5589224016025606	0.1779578565152813

0.0523666268348408	0.0561452984121739	0.1841915004758834
0.3860822733851271	0.5371101482651087	0.3663599583427757
0.6905371358144634	0.5667957668164609	0.3461188720261041
0.4662537931267649	0.3831370179306458	0.1580279508064658
0.4677977403820544	0.8490784963341103	0.1356954125961748
0.9735091860149018	0.3795230083333965	0.1410739391869382
0.9648303658004062	0.8764919125694794	0.1377513164704932
0.4679282525937283	0.6250345598552773	0.1603200015341877
0.4929379732262739	0.1356051879661354	0.1343312830936209
0.9883356134870285	0.6632295187808195	0.1358640042032729
0.9556968173921657	0.1209771311235153	0.1508761846301091
0.7128833391437601	0.3691991679774340	0.1304258312793733
0.6940555658865079	0.8566996473520215	0.1414515165002876
0.2155662572364694	0.3646949859570277	0.1502438747582371
0.2269268457716216	0.8921485738601284	0.1267087353379923
0.7190854569297797	0.6237928504145696	0.1627371490440631
0.7191620642429232	0.1226811797861378	0.1459353029479922
0.2319212239921702	0.6489255622658880	0.1408583194034300
0.2160130216672148	0.1200489771668270	0.1499824963664396
0.3868161599118224	0.4438928871908778	0.2755272158327382
0.3894517756841234	0.9598265828563337	0.2211594750584124
0.8859564232279965	0.4786427006523427	0.2280571918992526

0.8734038658995985	0.9572306095127626	0.2374737041154124
0.4045337065005388	0.7409227412083704	0.2502153193682700
0.3814364344002461	0.1880582972943060	0.2447593886936409
0.8812311468476599	0.2161003183812475	0.2497969550982728
0.6178404790391140	0.4484931539015163	0.2615110662684557
0.6279389226826010	0.9571900246066314	0.2304210495901267
0.1548909689398305	0.4766487038502703	0.2409997270917233
0.0905920284070083	0.9336963594165104	0.2416682404436878
0.6296180715882883	0.7289572309471259	0.2498080021821434
0.6403813508075739	0.2199431365536631	0.2432289288574084
0.1440365696893331	0.7415878452772037	0.2296948883408057
0.1289222017581896	0.2040180538777419	0.2525627253342597
0.6821991981557446	0.4664390954640660	0.3112637270086261
0.3275553726444467	0.4498436152222750	0.3209030553945361
0.9708586873306063	0.3603711381279909	0.0963031166010477
0.6808652791410599	0.3301716206610336	0.0894172494617326
0.2130654998994005	0.8814821251396434	0.0812889532541298
0.2091977991887863	0.3372448900389606	0.1065461541716299
0.9579047095225918	0.8626445408435971	0.0921997174988494
0.6750506713837745	0.8439546097094267	0.0965329864281789
0.3237754705952322	0.5292944670284775	0.3977971879553344
0.1592121253144387	0.5311694344237089	0.2756001208819208

0.2252571552866297	0.3837998468017506	0.3266155793996786
0.8600650858056806	0.9530113323498915	0.2829019048656947
0.4344564781876762	0.8157201154557410	0.2772661083562285
0.4029632954201287	0.1660868767570164	0.2852419470588007
0.1762832939394544	0.9766298239093819	0.2591754232000933
0.8726209377439333	0.2255940492100127	0.2947790278847792
0.6184742210159007	0.7353301160806847	0.2949384465894283
0.6505055483153366	0.2400614741113307	0.2881483352412377
0.1550249388568401	0.7721632751620614	0.2731094046490071
0.1080752177286222	0.1646115252346943	0.2940513459277208
0.4632778653262580	0.3970250248777386	0.1129323290538228
0.4427522428368114	0.7966999827921307	0.0976200599290558
0.7417302792116777	0.5731325133047935	0.3828562344065563
0.8509908127090423	0.4791871119014128	0.2699071316171542

Supplementary Dataset 44. The coordinates of the 2nd C-C cleavage step on nickel/cobalt surface. The energy of the 2nd C-C cleavage step on nickel/cobalt surface was calculated to be -418.17 eV [-388.43 eV -29.74 eV (formic acid)].

Ni Co O C H

1.00000000000000

11.8928003311000001	0.0000000000000000	0.0000000000000000
-5.9265986588999997	10.3212535711999998	0.0000000000000000

0.0000000000000000 0.0000000000000000 21.1420993804999995

Ni Co O C H

8 8 33 2 22

Direct

0.7429323605168291 0.4892149447520674 0.1028477596501102

0.7441205477249546 0.9930533528592768 0.0895361132769148

0.2406997653615567 0.4877058590328270 0.0884131569235182

0.2484636209267203 0.0015734868738049 0.1064031279341227

0.4995576593702321 0.7424506908724617 0.0966284648047934

0.5048061234107580 0.2475357444812790 0.0951127723456121

0.0005403868952219 0.7506418779974052 0.1072358007874050

0.9909543407238518 0.2410445397707229 0.0887154786689610

0.7368209012102714 0.7391853537790004 0.1032186832189275

0.7504726459062084 0.2445666723137437 0.0997807342322295

0.2570846715197380 0.7613036832508031 0.0974355239228706

0.2433012282227453 0.2407176180418764 0.0823417099737330

0.4723925386846374 0.4847128446710011 0.0501851604966907

0.5080837544983148 0.9942465152101075 0.0961822428394753

0.9925586125165644 0.4886165531613054 0.0952092312432428

0.9707926880748831 0.9895363958416796 0.0555183331529115

0.9052294682898212 0.8097848006471761 0.1840128028306060

0.8995323081989774 0.2986174331769668 0.1541818875090628

0.4129779076168136	0.8140732635570244	0.1539949829886909
0.9220728506867472	0.5740117448505950	0.1498871974416557
0.9214290450979030	0.0746218391459761	0.1244640109207951
0.4217756882230136	0.5549281765865899	0.1293879546345875
0.4323962403404186	0.0801916314929381	0.1454932080146400
0.6662378658390631	0.8304098926325942	0.1508628219085204
0.6561607862893383	0.3237608484407811	0.1551477578391897
0.1668070264271665	0.8321448238512803	0.1577497534821900
0.1521520237071310	0.3153481231561157	0.1363581187837449
0.6779396257127344	0.5792955275504605	0.1672391955520575
0.6714609918860015	0.0799008067422733	0.1379385148649851
0.1606988604270744	0.5770871200478391	0.1429553884749912
0.2201297392296067	0.1288896984063943	0.1588459292183722
0.7998799887388579	0.8956832715643018	0.0392963111165148
0.8324708236644029	0.4031074398617512	0.0582087647648838
0.3401423289152467	0.9121159758529021	0.0558348528431233
0.3073729369760431	0.3990328808664440	0.0270221864913961
0.8316820437483952	0.6657342115889321	0.0594553164422448
0.8344707804358730	0.1688891811207419	0.0395222855404911
0.3287896822414195	0.6636276092098565	0.0469793249734211
0.3383104355726655	0.1584326484362445	0.0465183272277178
0.5793136328829065	0.9137043602149156	0.0517746797811846

0.5754310488270533	0.4177038858695838	0.0547831671424003
0.0726918255283330	0.9219917889109120	0.0648354923999021
0.0590226781370431	0.4041787992442776	0.0510196315464263
0.5845631189787198	0.6703289000492351	0.0370165559603515
0.5997092062566162	0.1792786818393175	0.0456926646634180
0.0835521698455705	0.6742364904189608	0.0511704361776195
0.0769643924355664	0.1694358169859957	0.0265792893388474
0.6332230099043650	0.6247688649200931	0.2605342424331700
0.9983114726912045	0.9200508566181401	0.2717467970105016
0.6624998500757037	0.5434067372364084	0.2244260695390460
0.9330780922075141	0.9042192950699770	0.2172189631157875
0.0865545443455169	0.6846701462130291	0.0056340188037927
0.1025506182098875	0.2066682463065350	0.9845849370943116
0.8494852181657419	0.1970107397098031	0.9957059275638114
0.6227376526293640	0.5910419362207170	0.3034045459113379
0.4421253589149715	0.5355770116292292	0.1710041772026211
0.9125066358093283	0.5557463910657382	0.1949066401645761
0.8659470105122996	0.2365214109244744	0.1888597085229115
0.4113527191499532	0.8158367668234984	0.1997315272598654
0.1288048570888148	0.0950094592429765	0.1682943328396918
0.6555297165356707	0.8201458393322905	0.1963678125535070
0.6529715307516825	0.3527067201659544	0.1981708627170315

0.1707084199511316	0.8307706729261807	0.2034469091005042
0.1478557258507744	0.3001176583240506	0.1815307282120598
0.8346586427232483	0.6723610966010850	0.0137392456940020
0.3323145542866502	0.6738209964762073	0.0014359922973284
0.3421776116685863	0.1439636731972462	0.0016816326421023
0.6241078541638942	0.7226680565226284	-0.0004735266365905
0.6221329843013582	0.2074531844027421	0.0021490257667473
0.1520588966693784	0.5660860221324248	0.1884572297222663
0.9100332650233225	0.9805640263625868	0.2031555626040225
0.0175599802251134	0.0024830267997915	0.2908252648800692
0.4624190954245356	0.0990587985474463	0.1889219536399674

Supplementary Dataset 45. The coordinates of the 2nd C-C cleavage step on nickel surface. The energy of the 2nd C-C cleavage step on nickel surface was calculated to be -392.00 eV [-362.26 eV -29.74 eV (formic acid)].

Ni O C H

1.00000000000000

11.7038002014000000	0.0000000000000000	0.0000000000000000
-5.8513176253000001	10.2171014480999993	0.0000000000000000
0.0000000000000000	0.0000000000000000	21.1695003509999999

Ni	O	C	H
16	33	2	22

Direct

0.7383649893188751	0.7161636928867666	0.0879518415148158
0.7290630523293204	0.2119411284228118	0.0936474937701857
0.2330159821543531	0.7192788733650701	0.1024805171420624
0.2294341311479285	0.2132158341141256	0.0844006268731225
0.7275756441165635	0.9663960660958831	0.0906610203407025
0.7258659491932045	0.4561518915280265	0.1015116040683122
0.2306860088675282	0.9608451744699396	0.1038307397329373
0.2236736166414788	0.4608889832320983	0.0947171018303254
0.9838160515028502	0.7143481633055939	0.1026151924589994
0.9761295443693276	0.2132974700703209	0.0953234781838293
0.4795955560083057	0.7181272403530261	0.0929617156171311
0.4840130252608393	0.2137641120433855	0.0938067381789707
0.9821663265898050	0.9565110918027693	0.1043093967466902
0.9769183500609322	0.4546031805750866	0.1025428002275945
0.4794164490575195	0.9657851483274630	0.0936103502018262
0.4827448417225151	0.4576693653549004	0.0910075052699112
0.8328472617113688	0.8751390763038893	0.1963184158959697
0.8088081264340777	0.3729705302809264	0.1547282358783791
0.3278911915027660	0.8906457998385755	0.1556660472861182
0.8119194373518895	0.6304813640524972	0.1463555943946047
0.8079951802110773	0.1228930493564565	0.1371908949844345

0.3132867455163681	0.6292933407643644	0.1462838465524835
0.3214482141792838	0.1390163725516992	0.1439787462058583
0.5783159762965044	0.8979098780355235	0.1456800625689156
0.5572166135250012	0.3834357350486969	0.1526425966178287
0.0675038455488041	0.8815364772131646	0.1604644775093630
0.0703641825184300	0.3776311809254234	0.1552345188840832
0.5634677223974838	0.1263887563838363	0.1368505361158749
0.0657770513402100	0.6309936318760362	0.1510914590250941
0.0613566772231047	0.1339653392906059	0.1447608525843307
0.9126655044124883	0.7967761474144771	0.0546480571680729
0.8939824728238173	0.2921374104432559	0.0519377312080858
0.3902000105782033	0.8056549272403887	0.0531939274435688
0.4051368623106866	0.2940938931534061	0.0480655337060633
0.8947790041386416	0.5424565691303372	0.0541649166544529
0.8895492728964383	0.0279817720150207	0.0497247900126228
0.3954693273295120	0.5431673371658705	0.0477447167723650
0.3898902884679917	0.0396393939702734	0.0463017236356647
0.6548151485982298	0.8055235130742766	0.0529507599407084
0.6472637566806496	0.2962808208679720	0.0515537746165722
0.1468867620768731	0.7939286160244013	0.0588430701966355
0.1372282349194687	0.2972350484323100	0.0504556092338569
0.6503740526782872	0.5467163104085967	0.0457093485836013

0.6395494955347898	0.0400365412173203	0.0384849629664650
0.1451085653424095	0.5527692856748256	0.0513128668148886
0.1420953418510983	0.0350390739135519	0.0499890827421201
0.5509970225715182	0.6269846340249698	0.1568143554402015
0.8246104783935491	0.9379612938410999	0.2947999980528589
0.5929529870267253	0.5781915205328835	0.2504334922821490
0.5222735434208052	0.6235994124343420	0.2148884763505138
0.8262535537065292	0.9542445514218710	0.2311872666134770
0.5671990649670257	0.5801887081448462	0.2939976541049094
0.3454444022700708	0.6396974699948726	0.1898794788861014
0.8933464965838286	0.5384931892372856	0.0084315449272593
0.8784135864662450	-0.0029309072482397	0.0062984398588710
0.3881212685470605	0.5303071112667350	0.0023428996083041
0.3820322487937416	0.0231620937464941	0.0011710264129237
0.6460073125717908	0.5347319757720217	0.0003142871711906
0.6322289316087081	0.0249526849470030	0.9932344914831708
0.1417793391947180	0.5540710095039073	0.0056176439216933
0.1292313090671108	0.0096487392244181	0.0059053298081946
0.8027076732965484	0.6431923702789081	0.1910506727321670
0.7902450718339342	0.3495268366301795	0.1988370125103188
0.3532777551267378	0.9146568588511453	0.1992970599710642
0.0760338446488970	0.1633076118671331	0.1882462660520330

0.5971998758709518	0.9503250410401085	0.1838500675303627
0.5713254976573680	0.4150768467384622	0.1959392075370294
0.0638614491832918	0.8881807145422220	0.2058915637551916
0.0731995835556049	0.3982899349545676	0.1996791781160667
0.0734145754410753	0.6500590450233358	0.1959704781319344
0.3443253823759965	0.1561632701084698	0.1883198114150748
0.8173087884526117	0.0087378289279340	0.3149241021149199
0.8224752360397023	0.0414504703770602	0.2129953170782680

Supplementary Dataset 46. The coordinates of the 9B. hydrogenation step on cobalt surface. The energy of the 9B. hydrogenation step on cobalt surface was calculated to be -437.84 eV [-408.10 eV -29.74 eV (formic acid)].

Co O C H

1.00000000000000

12.1820001601999994	0.0000000000000000	0.0000000000000000
-6.0910000800999997	10.5499216077000000	0.0000000000000000
0.0000000000000000	0.0000000000000000	21.1058006286999991

Co	O	C	H
16	33	2	22

Direct

0.2979237671741717	0.2770483214396255	0.2021789829784597
0.3176002663616342	0.8112637124113357	0.1835975354457732

0.8002363985948866	0.2979146345510686	0.1995559226361383
0.7983122686903129	0.7998922266311239	0.1871476761484763
0.2957568035317117	0.5516443405975737	0.1750379769545332
0.3346377024197948	0.0686677038896586	0.1681096139801833
0.8185934045082542	0.5497861289465494	0.1639154033863267
0.7988168704981062	0.0502697767471093	0.1866675715921872
0.5445538224298526	0.2742955427265460	0.1836123176187182
0.5524359704673731	0.7989785999442385	0.1903843022497318
0.0452179643002384	0.2957749893665621	0.2038784217781900
0.0644236314602224	0.8174342587967265	0.1834652122898485
0.5593234955188250	0.5598835312105117	0.2064597926542425
0.5656912688236597	0.0474290138874984	0.1717310748214001
0.0622962647954947	0.5676499082003387	0.1876916818191131
0.0610173260604250	0.0649260595489594	0.1797687385200047
0.3759751848429796	0.5242152180222527	0.3704218115802247
0.6471302959032844	0.3903183155909815	0.3720732289009516
0.4732096541864758	0.3885320781686887	0.1619621734854965
0.4777890577508708	0.8579190419337975	0.1301924775548451
0.9747985916198008	0.3889484390632040	0.1508137396280680
0.9692394796368128	0.8796911320363533	0.1385943901061642
0.4668701011472591	0.6304000856715155	0.1585302175393451
0.4968959506711013	0.1337860537251572	0.1255824266938781

0.9911358440740758	0.6674814041167487	0.1436988814996513
0.9611694896590638	0.1356118506198816	0.1502615932442635
0.7145213483622271	0.3728754311106731	0.1370066951918686
0.7011742114118694	0.8622392804179835	0.1375364524446076
0.2186494416399209	0.3708886236921904	0.1528588939764596
0.2296315266775768	0.8917346887220426	0.1317626016053660
0.7209974237742051	0.6276322070608695	0.1653523796077176
0.7312475744075301	0.1395224711640568	0.1445865137733734
0.2332774089116676	0.6549143761469679	0.1459053094148821
0.2115547693304174	0.1261324136961882	0.1385189998178047
0.3861994767735430	0.4492509231226519	0.2755671004324439
0.4080538149783702	0.9717744536723578	0.2174827518792853
0.8861736427154769	0.4855797376403409	0.2365312303514215
0.8784368778550191	0.9647975087199515	0.2340638019976852
0.4125970451418954	0.7561233071000265	0.2464862155838858
0.3773038720784438	0.1847797779362968	0.2374923477603060
0.8809849434300205	0.2190584442113563	0.2544173496472728
0.6426272542188630	0.5000139762487837	0.2886272407108605
0.6348653238701406	0.9660924105462834	0.2243223602988139
0.1634811752512206	0.4887770047496848	0.2444128204521874
0.1013116462824729	0.9503949842255395	0.2416683669759851
0.6327759018994713	0.7387294380022089	0.2479576696638086

0.6380026281543849	0.2475638806115769	0.2540650530359224
0.1474189497318659	0.7519798014641043	0.2377882965395520
0.1317090654463813	0.2069292523961439	0.2522555676481954
0.5920901102554722	0.3997907514037749	0.3180796835178225
0.3223349737911059	0.4441389909375775	0.3213151777712733
0.9686181741938364	0.3689318687617780	0.1060034560808567
0.7010343789718732	0.3373722487194697	0.0945944861286584
0.2165805141526329	0.8746000531180478	0.0866140955744213
0.2071156214434986	0.3399565230288106	0.1095178202255673
0.9635095932253951	0.8606817060100356	0.0935596582211061
0.6829351798102316	0.8461869768509624	0.0926585875694043
0.3111693596197885	0.5071672935099658	0.4022477105497937
0.1871286982870044	0.5511150974046514	0.2781065719653704
0.2208164950489150	0.3733733309313493	0.3249033338276289
0.8674196284442353	0.9664039344961476	0.2794776489716400
0.4481343794265543	0.8333333314315718	0.2722716627554431
0.5854624913862320	0.1631451896056266	0.2720218449684056
0.1882193035454996	-0.0025924109856866	0.2581447522440474
0.8707345973067698	0.2196067117962998	0.2999043250088981
0.6226389392788833	0.7555253674036165	0.2918662523170903
0.5003381430085989	0.3159152872046611	0.3050793918610060
0.1575230237215068	0.7847286313903670	0.2806846937587456

0.1105694878676215   0.1558961387613979   0.2903941558681466

0.4773425817504460   0.4096212156394881   0.1172700651135017

0.4547972705152803   0.8096989044870189   0.0909249389204269

0.6022088424819800   0.3014264657784744   0.3849752816850133

0.8663219800646467   0.5024386960665065   0.2786459934688171

Supplementary Dataset 47. The coordinates of the 9B. hydrogenation step on nickel/cobalt surface. The energy of the 9B. hydrogenation step on nickel/cobalt surface was calculated to be -418.21 eV [-388.47 eV -29.74 eV (formic acid)].

Ni Co O C H

1.00000000000000

11.8928003311000001   0.0000000000000000   0.0000000000000000

-5.9265986588999997   10.3212535711999998   0.0000000000000000

0.0000000000000000   0.0000000000000000   21.1420993804999995

Ni Co O C H

8 8 33 2 22

Direct

-0.0041736272180760   0.2487689844845327   0.0900528249853526

-0.0031786126261091   0.7479917361440442   0.1052178089482225

0.5031124296010212   0.2483182382041181   0.1056365793322204

0.5009833267441833   0.7483511366654716   0.0937721626867572

0.2427671584436857   0.0069394994767375   0.1115608277491918

0.2431194245613275	0.4990972166384319	0.0927346464974026
0.7499030565418237	-0.0005601541431389	0.0878871804109244
0.7485884541415890	0.4899840595440292	0.1067651442258584
0.9761613855079764	-0.0016586609071471	0.0543073453111309
-0.0080209826350758	0.4990696502333995	0.0956470557684485
0.5072991800608374	-0.0047403849569021	0.0928789324611167
0.4714499858707065	0.4882207824817473	0.0555366510202399
0.2420896052171144	0.2500175628744770	0.0929856384305499
0.2541265519479641	0.7689349957041904	0.0932576741876172
0.7533528234376815	0.2463531782580939	0.0980580283810809
0.7457079953339745	0.7534347335860287	0.0959823102221484
0.9662078421945479	0.9025096930562395	0.2730698920134044
0.6916927140806282	0.5420904200835084	0.3022107290778124
0.0854393424548723	0.1802125409289441	0.0298615724432388
0.0838838337193119	0.6804415691995942	0.0460932931457795
0.5900132708843103	0.1780230359281486	0.0495788969736045
0.5839903332303714	0.6730180599132378	0.0348138803479656
0.0636984204391277	0.4265743613998605	0.0533712229925867
0.0697583065974156	0.9227908907997090	0.0666637175013554
0.5735212521143918	0.4228106617353970	0.0718412510118762
0.5849317768370449	0.9217436366788319	0.0478501074975051
0.3377671056442592	0.1706906399596858	0.0558880260586327

0.3291802290118895	0.6711624822486072	0.0462604942638144
0.8315912682650421	0.1699194280606409	0.0379703845110088
0.8242393166765943	0.6609734311756825	0.0589231973943457
0.3083659946327803	0.4064832736759914	0.0314546857886570
0.3295672675864654	0.9229286408332977	0.0550879737721195
0.8293571088471478	0.4052273921238738	0.0565677956337031
0.8053091931106030	0.9048868987024321	0.0346207288507060
0.2050463078752439	0.1258795256816308	0.1662647821746788
0.1653149450038242	0.5928568487417675	0.1441385150997340
0.6798902499299159	0.0860341741691906	0.1395137636243959
0.6493263005265986	0.5691858484261868	0.2019800968252445
0.1631421045240840	0.3347472567449907	0.1432538051097072
0.1606358385431709	0.8321125262719269	0.1573891388586099
0.6645016039375811	0.3247395620565799	0.1594539408907179
0.6661180487434171	0.8296666862513188	0.1468672028598363
0.4243887485025888	0.0811734557855825	0.1535213406734122
0.4279458743532684	0.5671703535095195	0.1317398412189912
0.9209565575850102	0.0700282515838649	0.1261659601908784
0.9269494087576963	0.5729609757361648	0.1472745489449497
0.4157126828200997	0.8254301273435070	0.1493144929092633
0.8999343082016487	0.3004119882747301	0.1544713111754608
0.8895550693236041	0.7944746086214444	0.1833775699199202

0.9247750818803444	0.8956828914256337	0.2129479837652023
0.7214741739253216	0.5590152547943846	0.2396105982418723
0.4383849298297602	0.0890953578890697	0.1987995616928006
0.9979468131556464	0.9907363442241642	0.2883599617165065
0.9222081932147110	0.9811193553511377	0.1908095986922304
0.1443473710086016	0.5885095697763210	0.1887236911285803
0.5981994902265102	0.1963064705005853	0.0045821891350729
0.6155748802939650	0.7193418817652263	-0.0049343124947998
0.3408047564073511	0.1588504636472734	0.0107116847734697
0.3265704249836994	0.6691813817693103	0.0004568514510355
0.8226828903822151	0.6543372042796587	0.0131671390277707
0.1611032826639522	0.3285446649619195	0.1889159244028437
0.1582470972220133	0.8137868926530919	0.2021190161641361
0.6783589220711965	0.3048622133320805	0.2021376470756427
0.6614657293577082	0.8291103942655831	0.1925651661847186
0.1127565542008765	0.0960896129895701	0.1703304631521193
0.4204154538065633	0.8396516925790036	0.1945404626426090
0.8620366668485480	0.2287294786556656	0.1846270576149482
0.8134070236394599	0.5628401373013000	0.2258114197778378
0.4546589648046550	0.5513214829488647	0.1726010188325572
0.7619128692739212	0.5389975757289306	0.3246078014954822
0.8335685358116670	0.1838555749479891	0.9927489108364558

0.1203412891691888   0.2231942826023171   0.9897082481549522

0.0853823349369385   0.6872054716207470   0.0003778281637824

Supplementary Dataset 48. The coordinates of the 9B. hydrogenation step on nickel surface. The energy of the 9B. hydrogenation step on nickel surface was calculated to be -392.35 eV [-362.61 eV -29.74 eV (formic acid)].

Ni O C H

1.00000000000000

11.7038002014000000   0.0000000000000000   0.0000000000000000

-5.8513176253000001   10.2171014480999993   0.0000000000000000

0.0000000000000000   0.0000000000000000   21.1695003509999999

Ni O C H

16 33 2 22

Direct

0.7578638808152515   0.7158347880579399   0.0551612120134456

0.7285991465644909   0.2044759389591686   0.0957268051429736

0.2326383855739472   0.7116572710424566   0.0954106195408310

0.2374545299588505   0.2171456077575786   0.0898608311363510

0.7286799498642117   0.9560779808265396   0.0956096351818438

0.7270970704701111   0.4540344334054919   0.1009676595375387

0.2332602288093734   0.9597650985315616   0.0977617090346521

0.2243009400690265   0.4641707931104271   0.0978998300872860

0.9898034763589542	0.7140161081679630	0.0922281864328027
0.9756064568541625	0.2084542780042332	0.0963990986866948
0.4753608088427949	0.7163110623168526	0.0896715997958242
0.4875379391768020	0.2095336455137254	0.0977919399542506
0.9883282856387101	0.9557775000015879	0.0977223067146281
0.9748135289997392	0.4499197096903853	0.0977959079176894
0.4774794002654470	0.9579791669289238	0.0900259057938464
0.4904229471161855	0.4592895913095310	0.1091574500762085
0.8305572586668285	0.8711998147930281	0.1918111935377079
0.8137431717132203	0.3710878874763476	0.1536281823391243
0.3302430103772194	0.8860181581474332	0.1489390393162916
0.8173096864364341	0.6426966300145095	0.1334398553554876
0.8089776037130761	0.1169023582384019	0.1389260051867962
0.3071774461079020	0.6237235143099678	0.1412973282327000
0.3231858190616010	0.1341211463761061	0.1458976079656799
0.5864719269854368	0.8855097699000111	0.1544314701200406
0.5659586346207247	0.3776388813962795	0.1620450314390647
0.0689896898685542	0.8786095547230883	0.1538235336207064
0.0847489900780955	0.3889662404309399	0.1573678508649899
0.5626162686771654	0.1130507567364062	0.1382951790122421
0.0663510869792040	0.6291988971305083	0.1444250924711509
0.0618208799624728	0.1307144458174557	0.1452406209574241

0.9382181648763084	0.8063793636277372	0.0389349604609636
0.8922307882657938	0.2825356416503720	0.0520761678202810
0.3894698804821376	0.8003822965250927	0.0473691692893010
0.4151165323629891	0.3031710127733173	0.0568714430185753
0.8924805719547261	0.5354110292990386	0.0480304256579754
0.8891291551971024	0.0266145850862931	0.0498529827574496
0.3917181339445026	0.5283728132159902	0.0581965523800940
0.3950148450932252	0.0441128042685200	0.0436575170895527
0.6516118083866053	0.7934889559598575	0.0576958273404501
0.6466900539095944	0.2868354974845859	0.0541063410641433
0.1525739980878645	0.7902164690265620	0.0519356883426203
0.1451063318416594	0.3057973871184237	0.0564735386529970
0.6329741853116684	0.5303017271893558	0.0455471174026111
0.6373671239453602	0.0259568380948096	0.0413398351609201
0.1415035471105483	0.5412643905167375	0.0456666917774945
0.1511101645093383	0.0476667523972253	0.0468332708473890
0.5467912308455893	0.5913894862770541	0.1969347740960490
0.8404104961547405	0.9222148218006140	0.2939517345066424
0.5201491790096433	0.6595439580118925	0.2911259858277349
0.4867157325452245	0.6303667614381480	0.2294026300365859
0.8275937852720834	0.9437529199482051	0.2319470912871585
0.4628790386474463	0.6896762387832579	0.3088939278138300

0.4030865150366250	0.6406677335274978	0.2103936049441035
0.8876926309730424	0.5244195442730536	0.0025891824905013
0.8777613084507280	0.0090839657198681	0.0047521759700940
0.3860514389168955	0.4915126131209085	0.0162940700324711
0.3926694612405741	0.0409455176335512	-0.0020616365744263
0.5924381826652704	0.4734541760614579	0.0092104428733431
0.6240881098516540	0.0010197169127166	-0.0029582952442475
0.1415660831815430	0.5319908644490688	0.0001779541444255
0.1396919973076924	0.0276881668406480	0.0021284157676998
0.8149132421020576	0.6859629706432436	0.1721520503625479
0.8115186099247486	0.3597507288548825	0.1989389335025023
0.3611797244309248	0.9167295430376565	0.1914175227946408
0.0683438546131581	0.1526292562485914	0.1897089156361568
0.5930122247493667	0.9621843257213465	0.1762627173706834
0.5967089110928355	0.4093236737821027	0.2043876603140284
0.0667302041068817	0.8887615767161680	0.1990730556002653
0.1065868301085886	0.4548072340377965	0.1900863726096306
0.0764746631068159	0.6581438330081755	0.1879849536896951
0.3425176479444555	0.1466954554130500	0.1906938654841764
0.8328993353421382	0.9878322150227068	0.3190221226553907
0.8176240760758738	0.0304272864939492	0.2199332813126883

Supplementary Dataset 49. The coordinates of the 2nd formic acid desorption step on cobalt surface. The energy of the 2nd formic acid desorption step on cobalt surface was calculated to be -436.87 eV [-347.65 eV –3*(29.74 eV) (formic acid)].

Co O C H

1.00000000000000

12.1820001601999994 0.0000000000000000 0.0000000000000000

-6.0910000800999997 10.5499216077000000 0.0000000000000000

0.0000000000000000 0.0000000000000000 21.1058006286999991

Co O C H

16 33 2 22

Direct

0.2979237671741717 0.2770483214396255 0.2021789829784597

0.3176002663616342 0.8112637124113357 0.1835975354457732

0.8002363985948866 0.2979146345510686 0.1995559226361383

0.7983122686903129 0.7998922266311239 0.1871476761484763

0.2957568035317117 0.5516443405975737 0.1750379769545332

0.3346377024197948 0.0686677038896586 0.1681096139801833

0.8185934045082542 0.5497861289465494 0.1639154033863267

0.7988168704981062 0.0502697767471093 0.1866675715921872

0.5445538224298526 0.2742955427265460 0.1836123176187182

0.5524359704673731 0.7989785999442385 0.1903843022497318

0.0452179643002384 0.2957749893665621 0.2038784217781900

0.0644236314602224	0.8174342587967265	0.1834652122898485
0.5593234955188250	0.5598835312105117	0.2064597926542425
0.5656912688236597	0.0474290138874984	0.1717310748214001
0.0622962647954947	0.5676499082003387	0.1876916818191131
0.0610173260604250	0.0649260595489594	0.1797687385200047
0.3759751848429796	0.5242152180222527	0.3704218115802247
0.6471302959032844	0.3903183155909815	0.3720732289009516
0.4732096541864758	0.3885320781686887	0.1619621734854965
0.4777890577508708	0.8579190419337975	0.1301924775548451
0.9747985916198008	0.3889484390632040	0.1508137396280680
0.9692394796368128	0.8796911320363533	0.1385943901061642
0.4668701011472591	0.6304000856715155	0.1585302175393451
0.4968959506711013	0.1337860537251572	0.1255824266938781
0.9911358440740758	0.6674814041167487	0.1436988814996513
0.9611694896590638	0.1356118506198816	0.1502615932442635
0.7145213483622271	0.3728754311106731	0.1370066951918686
0.7011742114118694	0.8622392804179835	0.1375364524446076
0.2186494416399209	0.3708886236921904	0.1528588939764596
0.2296315266775768	0.8917346887220426	0.1317626016053660
0.7209974237742051	0.6276322070608695	0.1653523796077176
0.7312475744075301	0.1395224711640568	0.1445865137733734
0.2332774089116676	0.6549143761469679	0.1459053094148821

0.2115547693304174	0.1261324136961882	0.1385189998178047
0.3861994767735430	0.4492509231226519	0.2755671004324439
0.4080538149783702	0.9717744536723578	0.2174827518792853
0.8861736427154769	0.4855797376403409	0.2365312303514215
0.8784368778550191	0.9647975087199515	0.2340638019976852
0.4125970451418954	0.7561233071000265	0.2464862155838858
0.3773038720784438	0.1847797779362968	0.2374923477603060
0.8809849434300205	0.2190584442113563	0.2544173496472728
0.6426272542188630	0.5000139762487837	0.2886272407108605
0.6348653238701406	0.9660924105462834	0.2243223602988139
0.1634811752512206	0.4887770047496848	0.2444128204521874
0.1013116462824729	0.9503949842255395	0.2416683669759851
0.6327759018994713	0.7387294380022089	0.2479576696638086
0.6380026281543849	0.2475638806115769	0.2540650530359224
0.1474189497318659	0.7519798014641043	0.2377882965395520
0.1317090654463813	0.2069292523961439	0.2522555676481954
0.5920901102554722	0.3997907514037749	0.3180796835178225
0.3223349737911059	0.4441389909375775	0.3213151777712733
0.9686181741938364	0.3689318687617780	0.1060034560808567
0.7010343789718732	0.3373722487194697	0.0945944861286584
0.2165805141526329	0.8746000531180478	0.0866140955744213
0.2071156214434986	0.3399565230288106	0.1095178202255673

0.9635095932253951	0.8606817060100356	0.0935596582211061
0.6829351798102316	0.8461869768509624	0.0926585875694043
0.3111693596197885	0.5071672935099658	0.4022477105497937
0.1871286982870044	0.5511150974046514	0.2781065719653704
0.2208164950489150	0.3733733309313493	0.3249033338276289
0.8674196284442353	0.9664039344961476	0.2794776489716400
0.4481343794265543	0.8333333314315718	0.2722716627554431
0.5854624913862320	0.1631451896056266	0.2720218449684056
0.1882193035454996	-0.0025924109856866	0.2581447522440474
0.8707345973067698	0.2196067117962998	0.2999043250088981
0.6226389392788833	0.7555253674036165	0.2918662523170903
0.5003381430085989	0.3159152872046611	0.3050793918610060
0.1575230237215068	0.7847286313903670	0.2806846937587456
0.1105694878676215	0.1558961387613979	0.2903941558681466
0.4773425817504460	0.4096212156394881	0.1172700651135017
0.4547972705152803	0.8096989044870189	0.0909249389204269
0.6022088424819800	0.3014264657784744	0.3849752816850133
0.8663219800646467	0.5024386960665065	0.2786459934688171

Supplementary Dataset 50. The coordinates of the 2nd formic acid desorption step on nickel/cobalt surface. The energy of the 2nd formic acid desorption step on nickel/cobalt surface was calculated to be -417.05 eV [-327.83 eV –3*(29.74 eV) (formic acid)].

Ni Co O C H

1.00000000000000

11.8928003311000001 0.0000000000000000 0.0000000000000000

-5.9265986588999997 10.3212535711999998 0.0000000000000000

0.0000000000000000 0.0000000000000000 21.1420993804999995

Ni Co O H

8 8 29 18

Direct

0.9972901131447621 0.2514905153849657 0.0935729663331087

-0.0036691129093739 0.7487397959175959 0.1064841833977634

0.5142678579556059 0.2572418847519741 0.1195257638260305

0.5072162879381580 0.7548262371998223 0.0885399810018196

0.2457550266056066 0.0091895207958455 0.1105870133595041

0.2434941226206576 0.4999437606682354 0.1005960588992782

0.7556184208862725 0.0037466034836654 0.0782500274773487

0.7545898874123487 0.4931175408756895 0.1272613958163952

0.9858531702824287 0.0107252985611567 0.0476621400670871

0.9940401409290895 0.5039662694186058 0.1052593965669689

0.5096073365614380 -0.0014853352318274 0.0916596438280924

0.4900679469591189 0.5058899755580257 0.0742253207913196

0.2502528546631879 0.2581325388973321 0.1000863970021724

0.2549375739104823 0.7691796784706890 0.0969815195046534

0.7575477211838728	0.2476053878781510	0.1043811750630049
0.7485510038550902	0.7627784389170600	0.0437079959977338
0.0915269878423579	0.1974363297360107	0.0347692664527207
0.0844495312452744	0.6803710348260045	0.0493775172771237
0.5843676917450754	0.1813576591963794	0.0549139681690794
0.5660191113770854	0.6612359840603651	0.0174258893749985
0.0660323485297979	0.4189028873035782	0.0623677955523002
0.0659684357804385	0.9260310921014256	0.0680790322239709
0.5738671465760785	0.4371753199411201	0.1189715522596201
0.5885309087222932	0.9266957371758926	0.0425208545199189
0.3471495559021341	0.1878950717954785	0.0642063495704502
0.3253135953166525	0.6692019288100159	0.0493145464459952
0.8369736464479427	0.1899804242972246	0.0396608904162172
0.8208093876978075	0.6523805962643709	0.0595323385362539
0.3145138388985620	0.3974053149388023	0.0494724401053405
0.3277350020495903	0.9237164554770111	0.0577837232773986
0.8305841353685565	0.4117268502516804	0.0689837348054582
0.8249772265412889	0.9368010568796986	0.0120358282098798
0.1961416449924296	0.1256809699662248	0.1612827608640204
0.1664588394406795	0.5990996908203458	0.1490354674764151
0.6843262530734823	0.0781355386259254	0.1361556148730695
0.1641301402053985	0.3282703888267031	0.1484744213006992

0.1616097403218450	0.8389630275806725	0.1581267228426914
0.6800494754218130	0.3099932344586039	0.1700333100512904
0.6914202236860627	0.8197769305150209	0.1218440176730187
0.4228226303448711	0.0797689490846539	0.1559539078470026
0.4270008159691487	0.5887813940700319	0.1391673879482636
0.9222813754888738	0.0759151561205920	0.1190943406552869
0.9338520661969162	0.5886984784285892	0.1570609708784199
0.4269168232319704	0.8337372364612824	0.1475107259031282
0.9134730441008940	0.2936390587472806	0.1605465763416941
0.4261949224488535	0.0738847743888940	0.2015528354921237
0.1464345778333587	0.6004254939315746	0.1934746778797033
0.5890109475203326	0.1985964697508859	0.0098974562386477
0.5394836999866308	0.6591280971108731	-0.0260877128551723
0.3582621560491606	0.1995057839849371	0.0187280323695211
0.3107440480300169	0.6638779689245087	0.0040454290567333
0.8328334664389325	0.6062077885506865	0.0244768771963212
0.1570330316374592	0.3066193229588428	0.1930964662989828
0.1546056067910684	0.8213774231284291	0.2030961053636406
0.6893520042048499	0.2872448816593949	0.2129426794892858
0.7197175807505365	0.8257369953502645	0.1652758434744412
0.1019817696345527	0.0867002824031339	0.1656554613632490
0.4438662300047124	0.8517415799472804	0.1922451661791530

0.8752512798600647 0.2175846591807533 0.1880805632214620

0.4519307084580103 0.5870587852789440 0.1825861896683232

0.8529923142872700 0.2332186612412613 0.9990371306414377

0.1390689758787481 0.2574953854988619 0.0006339087612071

0.0899220316713775 0.6920389894025283 0.0038945683769321

Supplementary Dataset 51. The coordinates of the 2nd formic acid desorption step on nickel surface. The energy of the 2nd formic acid desorption step on nickel surface was calculated to be -390.89 eV [-301.67 eV –3*(29.74 eV) (formic acid)].

Ni O H

1.00000000000000

11.7038002014000000 0.0000000000000000 0.0000000000000000

-5.8513176253000001 10.2171014480999993 0.0000000000000000

0.0000000000000000 0.0000000000000000 21.1695003509999999

Ni O H

16 29 18

Direct

0.7611947883224410 0.7202165349082315 0.0548682096435683

0.7281562675603110 0.2064942665654190 0.0956884889170960

0.2358651021794920 0.7107877189886713 0.0986334442154301

0.2345107502405019 0.2152899766819021 0.0932089478794647

0.7288456762424116 0.9584975082905041 0.0934114976958415

0.7267406444202922	0.4557646438299546	0.1009558817796134
0.2327559266446676	0.9580639658003324	0.1004595915911392
0.2246944622396636	0.4630818678416402	0.0975985816260903
0.9917127735049290	0.7133365752543622	0.0928949457862083
0.9737093162275563	0.2076008728158496	0.0961065747981052
0.4764496585268811	0.7135143157012906	0.0936086441701702
0.4854655162341274	0.2085219235908930	0.0970158070368433
0.9898547364084227	0.9567975206145938	0.0924710753783442
0.9758744507099697	0.4507574010181077	0.0998262322347448
0.4800047815823919	0.9588596054007748	0.0950350138187897
0.4866636323888949	0.4571299112036363	0.0949588880185332
0.8114081163735907	0.3723609387234720	0.1543090249052136
0.3284617839256428	0.8834477478405633	0.1532748348259804
0.8169428137312338	0.6457189016293773	0.1339134391609472
0.8082511278150675	0.1165123580167134	0.1376141341598763
0.3096680630013387	0.6194526641718425	0.1428461835907626
0.3229537921808127	0.1343129708593911	0.1473529199832831
0.5866163866145799	0.8871768409166690	0.1507143831492745
0.5618225947089824	0.3941611973371104	0.1585213347789662
0.0619499851038111	0.8806220716169749	0.1546089824484051
0.0838363593468514	0.3891021094476768	0.1559432649976688
0.5633215096626966	0.1190250058492397	0.1391481123937966

0.0672199863586009	0.6299469905112964	0.1459549597836670
0.0520419551639896	0.1229489128836328	0.1445402759538206
0.9410680144691006	0.8036757498527551	0.0377103637279236
0.8925221987651130	0.2846482718967548	0.0525068226056168
0.3929693456488466	0.8010525778171057	0.0519262769579848
0.4081674699380837	0.2935058548808102	0.0537668047416492
0.8937308944058674	0.5357774156167165	0.0495246800234516
0.8877332874108160	0.0264940188945076	0.0461260003415079
0.3883299747068554	0.5302487749757454	0.0520196493404685
0.3942606660914831	0.0386777389908219	0.0465812112614983
0.6510600394844103	0.7930784431186678	0.0577336276477225
0.6452050070798697	0.2884347915933646	0.0541210465515870
0.1560503049636473	0.7896091868654307	0.0545045806036279
0.1439321355935676	0.3029397384139126	0.0572183021524447
0.6401371861063523	0.5335171585549371	0.0398981543684788
0.6364745388698498	0.0283417593209174	0.0414838277836149
0.1426775395733577	0.5421833700102638	0.0466521095256225
0.1565154230633963	0.0497066192985121	0.0471161585461131
0.8911202090497126	0.5253446198044059	0.0040844487701541
0.8731233258609538	0.0129424165648818	0.0008828285739168
0.3766876456683508	0.5042690044864907	0.0077778216533973
0.3896040247961869	0.0289869451222329	0.0010798815127835

0.6153745790766428	0.4860000631151155	0.0001403909773450
0.6174284012238591	0.0017393389046370	0.9975498644500613
0.1422253961575788	0.5360318233406152	0.0009962871019873
0.1546450225944439	0.0400088401652395	0.0016701284939366
0.8257712941209575	0.6834207564413347	0.1755957435152697
0.8053298671076594	0.3551554007177002	0.1992384252858188
0.3555551033742388	0.8999380116332931	0.1971605619874801
0.0370319202584388	0.1269808336992838	0.1893495813101295
0.6084162972325498	0.9438857594987756	0.1875880516836565
0.5856136756925850	0.4253223343102006	0.2015065213291507
0.0792877134604133	0.9095379337599723	0.1981462763618077
0.1120762209294470	0.4451323079472616	0.1931535568459263
0.0774133219948115	0.6550376743282186	0.1901287551359826
0.3445705978162152	0.1451630887790163	0.1919509639258125
